# Taxonomic revision of the Afrotropical hover fly genus *Senaspis* Macquart (Diptera, Syrphidae)

**DOI:** 10.3897/zookeys.1003.56557

**Published:** 2020-12-14

**Authors:** Marc De Meyer, Georg Goergen, Kurt Jordaens

**Affiliations:** 1 Royal Museum for Central Africa, Invertebrates Section and JEMU, Leuvensesteenweg 13, B3080 Tervuren, Belgium Royal Museum for Central Africa Tervuren Belgium; 2 International Institute of Tropical Agriculture, Biodiversity Centre, 08 BP 0932 Tri Postal, Cotonou, Benin International Institute of Tropical Agriculture, Biodiversity Centre Cotonou Benin

**Keywords:** Africa, DNA barcoding, Eristalinae, flower fly

## Abstract

The representatives of the Afrotropical hover fly genus *Senaspis* Macquart (Diptera) are revised. In total, ten species are recognized. *Senaspis apophysata* (Bezzi) is herewith placed as junior synonym of *S. flaviceps* Macquart, *S. livida* (Bezzi) is herewith placed as junior synonym of *S. dentipes* (Macquart) and *S. griseifacies* (Bezzi) is herewith placed as junior synonym of *S. haemorrhoa* (Gerstaecker). All species are redescribed and an identification key is provided. DNA barcoding analysis (7 species, 64 barcodes) showed that the technique can be used to unambiguously identify the species. The relationships among the different *Senaspis* species are discussed based on morphological and DNA data.

## Introduction

Hover flies (Diptera, Syrphidae) constitute a diverse family of true flies, comprising ca. 6200 species (Pape et al. 2013). The group is poorly represented in the Afrotropical Region with slightly over 600 species known (Ssymank et al. in press). However, this could be a reflection of the limited surveying and studies conducted on this group in the region (Dirickx 1998). Nevertheless, syrphids most likely play an equally important role as pollinators, predators or decomposers, in a number of ecosystem services, as they do in other biogeographical regions (Inouye et al. 2015). Reviews on the current state are provided by Dirickx (1998), Whittington (2003) and Ssymank et al. (in press) and there is a clear need to improve the general knowledge on this group of flies.

Among the elements hampering an improved knowledge of the Afrotropical syrphid diversity is the lack of identification tools for most genera (Ssymank et al. in press). Eristalines are not an exception to this with relatively recent keys (i.e., published over the last 30 years) available only for the genera *Ceriana* Rafinesque, 1815 (Thompson 2013), *Chasmomma* Bezzi, 1915 (Kassebeer 2000), *Graptomyza* Wiedemann, 1820 (Whittington 1992, 1994), *Megatrigon* Johnson, 1898 (Doczkal et al. 2016), *Syritta* Le Peletier & Audinet-Serville, 1828 (Lyneborg and Barkemeyer 2005) and *Phytomia* Guérin-Méneville, 1834 (De Meyer et al. 2020). Currently, the African fauna of a number of eristaline genera is under revision, aiming to improve our knowledge for this group. This paper presents a taxonomic revision for the Afrotropical representatives of the genus *Senaspis* Macquart, 1850.

*Senaspis* was described by Macquart in 1850 with type species *Senaspis flaviceps* Macquart, 1850. It is an exclusively Afrotropical genus, although the types of some species were labelled as originating from the Neotropical (*S. nigripennis* (Macquart, 1855), Colombia) or Oriental (*S. dentipes* (Macquart, 1842), Java) Regions (see below under comments under respective species for details). Kertész (1910) made the unjustified emendation of the genus name to *Stenaspis*. As the latter is pre-occupied by *Stenaspis* Audinet-Serville, 1834 (Coleoptera: Cerambycidae), Bezzi (1912) proposed *Protylocera* Bezzi, 1912 as new name. However, Curran (1927a) outlined that the initial change to *Stenaspis* was unnecessary and thus the name *Senaspis* is retained as valid. Loew (1860) suggested using the name *Plagiocera* Macquart, 1842 for *S. maculipennis* (Loew, 1858) and related species, which Loew did not list. The genus *Plagiocera* was proposed by Macquart (1842) for the Nearctic *Milesia cruciger* Wiedemann, 1830. However, the name is preoccupied by *Plagiocera* Klug, 1834 (Hymenoptera: Cimbicidae) (see Evenhuis et al. 2016). Hull (1949) proposed the name Triatylosus Hull, 1949 as subgenus for a single species with additional facial tubercles on either side of the medial one: *Xylota dibaphus* Walker, 1849. This subgeneric division was not followed by Smith and Vockeroth (1980) in the Catalogue of Afrotropical Diptera. The latter listed 14 *Senaspis* species, but they recorded *Eristalis cupreus* Macquart, 1842 both under *Senaspis* and under *Eristalinus* Rondani, 1845. Whittington (1993) listed it solely under the genus *Senaspis*. Dirickx (1998) listed *cupreus* Macquart under the genus *Eristalis* Latreille, 1804. The study of the type material of this species (at MNHN) corroborates that it is not a *Senaspis* species but a species of the genus *Eristalinussensu stricto* and thus, it is excluded from this revision.

Representatives of *Senaspis* are reported from wetlands and riverbanks, especially when visited by domestic or wild mammals, as well as other water bodies (Ssymank et al. in press). Several specimen labels indicate rotting stems of banana stumps or decaying coconut trunk (Harmer 1913) as larval habitat and Copeland et al. (1999) described the larvae of *S. haemorrhoa* (Gerstaecker, 1871) collected from a stump of the cycad *Encephalartos tegulaneus* Melville (Zamiaceae). Furthermore, larvae of *Senaspis* species have been reported from sewage ponds and pig slurry (Curtis and Hawkins 1982; Ssymank et al. in press). Though adult Syrphidae are likely among the most important pollinators within the Diptera (Doyle et al. 2020), literature records of flower visiting *Senaspis* are still scanty (Nilsson et al. 1986) probably due to the unavailability of comprehensive identification keys for the genus.

Recent expeditions by staff members of the Royal Museum for Central Africa and the International Institute of Tropical Agriculture, as well as specimens collected independently by other research groups, have expanded the material available, including specimens suitable for DNA extraction. The main objectives of this manuscript are, therefore, to provide a taxonomic revision of all representatives of the genus *Senaspis*, to present an identification key and to discuss the interrelationships based on morphological and DNA data.

## Materials and methods

Material for study was obtained from the following institutions:

**AMGS**Albany Museum, Grahamstown, South Africa;

**AMNH**American Museum of Natural History, New York, USA;

**BMSA**Bloemfontein Museum, Bloemfontein, South Africa;

**CDFA**California Department of Food and Agriculture, Sacramento, USA;

**CNC**Canadian National Collection of Insects, Arachnids and Nematodes, Ottawa, Canada;

**ICIPE** International Centre of Insect Physiology and Ecology, Nairobi, Kenya;

**IITA**International Institute of Tropical Agriculture, Cotonou, Benin;

**KBIN** Koninklijk Belgisch Instituut voor Natuurwetenschappen, Brussels, Belgium;

**KMMA**Koninklijk Museum voor Midden-Afrika, Tervuren, Belgium;

**MCSNG** Museo Civico di Storia Naturale “Giacomo Doria”, Genoa Italy;

**MNB** Museum für Naturkunde, Berlin, Germany;

**MNHN** Muséum national d’Histoire naturelle, Paris, France;

**NHMUK**Natural History Museum, London, UK;

**NMSA**KwaZulu-Natal Museum, Pietermaritzburg, South Africa;

**NRMS**Naturhistoriska Riksmuseet, Stockholm, Sweden;

**MZH**Finnish Museum of Natural History, Zoology unit, Helsinki, Finland;

**OBPE** Office Burundais pour la Protection de l’Environnement, Bujumbura, Burundi;

**OXUM** Oxford University Museum, Oxford, UK;

**ZFMK**Zoologisches Forschungsmuseum Alexander Koenig, Bonn, Germany.

Morphological terminology largely followed Thompson (1999). Morphological observations were made with a Leica MZ8 stereomicroscope. Digitial images were obtained using the set-up as outlined in Brecko et al. (2014). Stacking was done using the software Zerene Stacker (zerenesystems.com/cms/home). Male genitalia were dissected using forceps and soaked 24–48 hours (depending on time required for sufficient clearance) in a cold 10% KOH solution after which they were transferred to acetic acid for 24 hours. Once sufficiently cleared, they were transferred to glycerine. Digital images were taken with a Leica MZ16 microscope and mounted Leica DFC500 digital camera using Leica Application Suite (LAS) automontage software (version 3.8). Measurements of wing and body lengths were taken by use of a calibrated ocular through the stereomicroscope, and are based on ten specimens (whenever available) randomly chosen, from which minimum and maximum length were selected. Body measurements were taken between the anterior margin of the frons and the posterior end of tergum IV; wing measurements between the tegula and the apex of the wing. For type material, text on identification and location labels is given ad verbatim. Text is indicated in quotation marks (“ ”) and each line on the label is separated by a double forward slash (//). Text not given on labels (i.e., collection depository) is given in square brackets ([]).

Literature references are given for original taxon descriptions under each species. For full bibliographic references, we refer to Dirickx (1998).

Procedures for DNA barcoding followed Jordaens et al. (2015). Briefly, genomic DNA was extracted from a single leg using the NucleoSpin Tissue Kit (Macherey-Nagel, Düren), following the manufacturer’s instructions. PCR reactions were undertaken in 25 µl reaction volumes that contained 1.5 mM MgCl_2_ in 1 × PCR buffer (Invitrogen), 0.2 mM of each dNTP, 0.2 µM of each primer and 0.5 units of Taq polymerase (Invitrogen). The DNA barcode fragment of the mitochondrial cytochrome *c* oxidase subunit I (COI) gene was amplified using primer pair LCO1490 and HCO2198 (Folmer et al. 1994). The PCR profile was an initial denaturation step of 5 min at 95 °C, followed by 35 cycles of 45 s at 95 °C, 45 s at an annealing temperature of 45 °C and 1.5 min at 72 °C, and ending with a final extension step of 5 min at 72 °C. PCR products were purified using the GFX PCR DNA Purification Kit (GE Healthcare) and diluted in 15 µl of sterile water or using the ExoSap protocol (Invitrogen) following the manufacturer’s instructions. PCR-products were bidirectionally sequenced using the ABI PRISM BigDye Terminator v3.1 Cycle Sequencing Kit and run on an ABI3130xl Genetic Analyzer. Sequences were assembled in SeqScape v2.5 (Life Technologies) and inconsistencies were checked by eye on the chromatogram.

In total, we obtained 20 new DNA barcodes and complemented this dataset with 21 DNA barcodes of Jordaens et al. (2015) (GenBank accession numbers: KR831198–KR831218), 19 unpublished barcodes obtained by CNC, and four unpublished barcodes from MZH (GenBank Accession numbers MW066957–MW066999, see Suppl. material 2: Table S1). Hence, the total *Senaspis* DNA barcode dataset comprised 64 sequences. *Eristalis tenax* (Linnaeus, 1758) was used to root the topologies. No DNA barcodes could be obtained for *S. melanthysana* (Speiser, 1913), *S. pennata* (Hervé-Bazin, 1914), or *S. xanthorrhoea* (Bezzi, 1912).

A Neighbor-Joining (NJ) tree (Saitou and Nei 1987) was constructed using the K2P model in MEGA v7 (Kumar et al. 2016) (see Suppl. material 1: Fig. S1), and pairwise p-distances (i.e., the proportion of sites at which two sequences differ) within and among species were calculated. Moreover, a NJ and a Maximum Likelihood (ML) topology (Guindon and Gascuel 2003) were constructed after removing identical sequences with DAMBE v.7 (Xia 2018) (Fig. 114). Branch support in the NJ analysis was evaluated using 1,000 bootstrap replicates. For the ML analysis, the dataset was partitioned according to codon position and the Akaike Information criterion in jModelTest v.2 (Guindon and Gascuel 2003; Darriba et al. 2012) was used to select the most appropriate model of evolution. These were the F81+I+G (first position), GTR+I+G (second position), and GTR+G (third position) model, respectively. Then, Garli v.2.01 (Zwickl 2006) was used to perform the ML analysis (two replicates; 500 bootstrap pseudoreplicates) taken into account the most appropriate models of evolution for each of the three codon positions. In each analysis, *Eristalis tenax* was constrained as root. Bootstrap values were considered to be meaningful if ≥ 70% (Hillis and Bull 1993). Specific results of the DNA barcode analysis are discussed in the Comments parts under each taxon of the Taxonomy and systematics section when relevant and in the Discussion.

## Taxonomy and systematics

### Senaspis

Taxon classificationAnimaliaDipteraSyrphidae

Macquart, 1850: 437.

F00A8037-CA11-5B34-A88E-8512612B3608

Senaspis Macquart, 1850: 437. Type species: Senaspis flaviceps Macquart, 1850 (by monotypy).
Protylocera Bezzi, 1912: 414 [unnecessary replacement name].
Triatylosus Hull, 1949: 398 (as subgenus). Type species: Xylota dibaphus Walker, 1849 (by original designation).

#### Generic diagnosis.

*Senaspis* species are morphologically characterized by the combination of the following characters: eyes bare and maculate (less conspicuous in dried specimens), dorsal facets in male usually only slightly larger than ventral facets, more pronounced so in *S. dibapha* (Walker, 1849) and *S. nigrita* (Bigot, 1859); antennal arista bare; frons in lateral view with distinct protuberance; wing with cell r_1_ usually closed and petiolate, or narrowly open at most (see Fig. 69) (if distinctly open then hyaline, without dark markings; see *Senaspis pennata*; Fig. 79); vein R_4+5_ strongly sinuate; thorax with triangular (dorsomedial) part of anepimeron bare, katepimeron bare or pilose at least in dorsal part; metatibia with conspicuous pile on at least apical half ventrally (except in *S. flaviceps*).

### Key to the Afrotropical species of *Senaspis*

**Table:** 

1	Wing hyaline (Fig. 79), without dark markings; cell r_1_ open for a distance equal to height of cell. Scutellum not marginate apically (Fig. 63). Metatibia with long (equal in length to width of tibia) and very dense pile on ventral and dorsal margins along its entire length (Fig. 90)	***S. pennata* (Hervé-Bazin)**
–	Wing largely dark (Figs 65, 68) or at least with some darker markings (Figs 64, 72–78); cell r_1_ usually closed (if open then for distance shorter than height of cell). Scutellum marginate apically (Figs 52–60). Metatibia with short pile along ventral and dorsal margins (Figs 83–84), at most with longer pile (but never as long as width of tibia) on apical half to two-thirds (Figs 80–82, 85–89)	**2**
2	Wing hyaline with a distinct dark brown macula on medial part (Figs 64, 73, 78)	**3**
–	Wing either largely dark (Figs 65, 68) or fumose with darker but less defined areas (Figs 72, 74–77)	**5**
3	Postabdomen (terga posterior to tergum IV) and at least part of tergum IV conspicuously orange to orange-red, with pale orange pile (Fig. 95), sometimes all abdominal terga largely yellow to yellow-orange (Fig. 100). Metafemur with two ventral swellings on apical part, the proximal swelling less developed (Fig. 85)	**4**
–	All abdominal terga dark and with dark pile, sometimes postabdomen reddish but then without pale orange pile (Fig. 91). Metafemur ventrally with a single swelling on apical part (Fig. 80)	***S. dentipes* (Macquart)**
4	All abdominal terga dark yellow to yellow-orange, with pale orange pile except dark pile medially on posterior half of terga II and III (Fig. 100)	***S. xanthorrhoea* (Bezzi)**
–	Abdomen darker, orange colour and pale pile restricted to, at most, posterior margin of tergum III, whole of tergum IV and postabdomen (Fig. 95)	***S. haemorrhoa* (Gerstaecker)**
5	Scutellum colour pale yellow, strongly contrasting with dark scutum (Fig. 59). Metafemur ventrally with two distinct swellings in apical part (Fig. 87). Wing veins yellowish basally, slightly darkened in apical fourth (Fig. 75)	***S. nigrita* (Bigot)**
–	Scutellum colour largely concolourous with scutum, at least along the basal half (Figs 53–56, 58, 60–61). Metafemur ventrally with a single swelling in apical part (Figs 84, 86, 88, 89), if there is a weak second swelling basally of major one, then former without dark setae (Fig. 81). Wing veins yellowish or dark (Figs 65, 68, 74, 76)	**6**
6	Wing largely dark brown, hyaline area restricted to narrow band along posterior margin or paler area in apical part, distal third of cell br and medial part of cell bm concolourous (Figs 65, 68); lower calypter white to yellow, with pale fringe	**7**
–	Wing yellow to pale brown infuscated, with darker area at most restricted to anterior half of wing, distal third of cell br darker coloured than medial part of cell bm (Figs 72, 74, 76); lower calypter black to pale brownish, with dark or pale fringe	**8**
7	Scutum with conspicuous white to yellow long pile (Figs 54, 55), contrasting with dark pile on pleura (Fig. 3). Metafemur thickened, black (Fig. 82). Face dark, with a single medial tubercle (Figs 20, 22)	***S. elliotii* Austen**
–	Scutum without conspicuous pale pile, with conspicuous black pollinosity along transverse suture (Fig. 53). Metafemur slender, reddish (Fig. 81). Face usually red, rarely brownish, with medial and lateral tubercles (Figs 16, 18)	***S. dibapha* (Walker)**
8	Face and antennae yellow to red (Figs 24–27). Wing lower calypter black with white fringe (Fig. 72). Male metafemur with distinct basoventral tubercle (Fig. 83)	***S. flaviceps* Macquart**
–	Face and antennae dark (Figs 32–35, 40–45). Wing lower calypter pale brown to brown, with pale or dark fringe (Figs 74, 76, 77). Male metafemur without basal tubercle (Figs 86, 88)	**9**
9	All abdominal sterna with long dark pile (Fig. 6). Metafemur moderately thickened, with ventral margin straight or slightly convex (Fig. 86). Wing lower calypter with dark fringe (Fig. 74)	***S. melanthysana* (Speiser)**
–	Abdominal sterna I–III with long pale pile (Fig. 10), darker in other sterna. Metafemur strongly thickened, with ventral margin concave (Fig. 88). Wing lower calypter with pale fringe (Figs 76, 77)	**10**
10	Abdominal terga with short pale pile on lateral margins (Fig. 98). Scutellum with pale pile	***S. umbrifera* (Walker)** (holotype)
–	Abdominal terga with short dark pile or intermixed pale and dark pile on lateral margins (Fig. 99). Scutellum usually with dark pile on disc (rarely completely pale pilose)	**S. near umbrifera (Walker)** (see comments under *umbrifera* for discussion on the identity of this taxon)

### Taxonomic treatments

### Senaspis dentipes

Taxon classificationAnimaliaDipteraSyrphidae

(Macquart, 1842)

5FE515FA-B574-50EA-8BA4-EAC3B32B9FB0

Figs 1 , 12–15 , 52 , 64 , 80 , 91 , 102

Eristalis dentipes Macquart, 1842: 97.
Helophilus aesacus Walker, 1849: 609. Syn. by Smith and Vockeroth (1980).
Plagiocera maculipennis Loew, 1858: 381. Syn. by Austen (1909).
Eristalis latevittatus Bigot, 1858: 365. Syn. by Kertész (1910).
Protylocera aesacus var. livida Bezzi, 1912: 421. Syn. nov.

#### Differential diagnosis.

**Figures 1, 2.**
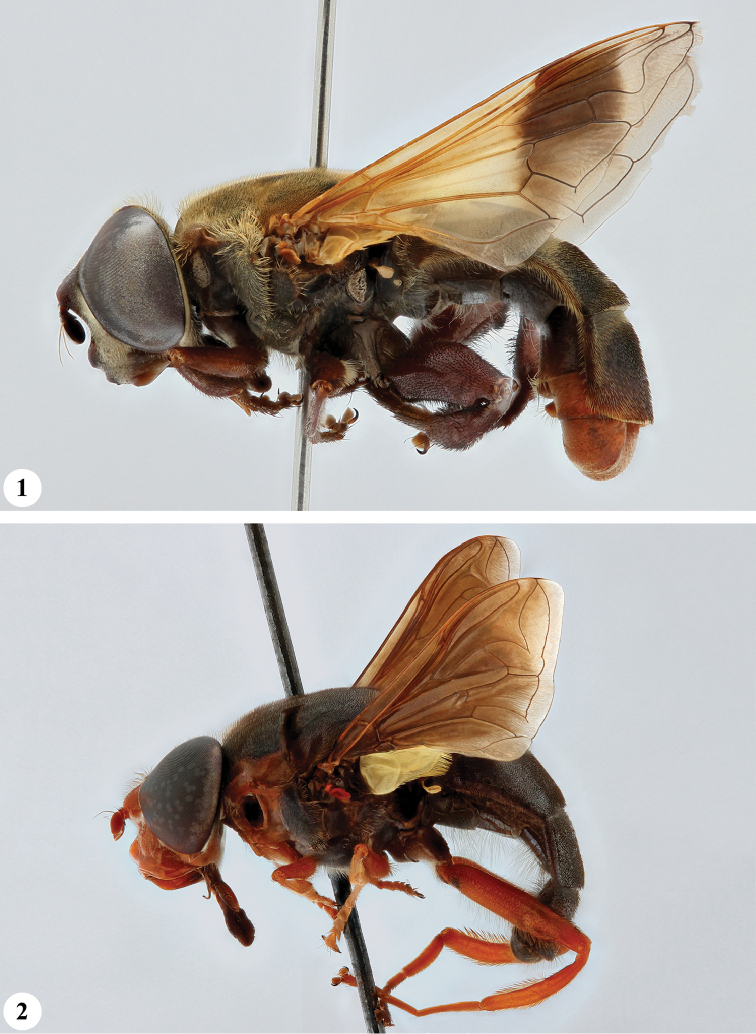
*Senaspis* species, habitus, lateral view **1***S. dentipes* (Macquart) (♂) **2***S. dibapha* (Walker) (♂).

A predominantly subshiny brownish species (Fig. 1), without distinct pilosity. The wing (Fig. 64) is largely hyaline but with a distinct dark brown medial macula. It can be differentiated from other *Senaspis* species with a distinct wing macula (*S. haemorrhoa*, *S. xanthorrhoea*) by the absence of orange pile on the abdominal terga and the presence of a single distinct ventral swelling in the apical fifth of the metafemur (Fig. 80) (two swellings in *S. haemorrhoa* and *S. xanthorrhoea*).

#### Examined material.

*Eristalis dentipes* Macquart: Lectotype (hereby formally designated and published; see comments), male, “N° 1177. // Eristalis // dentipes.” “272 // 97” “70” “MNHN, Paris // ED6399” “LECTOTYPE” “TYPE // Vockeroth ‘69” “LECTOTYPE // Eristalis // dentipes Macq. // Desig. Thompson 1977” [MNHN].

*Helophilus aesacus* Walker: Holotype, male, “Holo- // type” “Type” “Helophilus // aesacus // Wlk.” “Sierra Leone” [NHMUK].

*Eristalis latevittatus* Bigot: Holotype, female, “2423 // 83” “TYPE” “Eristalis // latevittatus ♀. // n.sp. J. Bigot. // Gabon. // Coll. Thomson.” [MNHN].

Protylocera aesacus var. livida Bezzi: Lectotype (hereby designated), male “Is. Fernando Poo // Basile // 400–600 m.s.m. // VIII–IX.1901. L. Fea leg.;” “Typus” “SYNTYPUS // Protylocera // aesacus var. // livida Bezzi, 1912” “Protylocera // aesacus Walker // v. livida // det. Bezzi, 1912” “Museo Cvico // di Genova” “Protylocera // aesacus // var. lívida n.” “LECTOTYPUS” [MCSNG]. Paralectotype, female, “Is. Fernando Poo // Musola // 500–800 m.s.m. // I III.1902. L. Fea leg.;” “SYNTYPUS // Protylocera // aesacus // var. lívida” “Protylocera // aesacus Walker // v. livida // det. Bezzi, 1912” “Museo Civico // di Genova” “PARA- // LECTOTYPUS” [MCSNG].

**Figures 3, 4:**
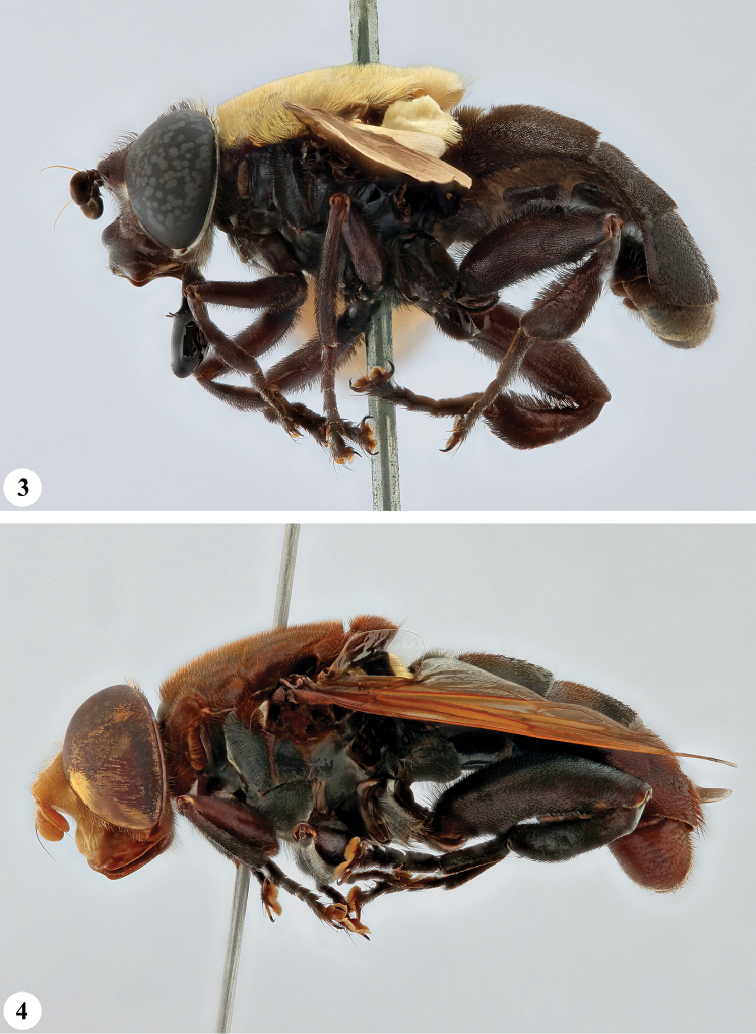
. *Senaspis* species, habitus, lateral view **3***S. elliotii* Austen (♂) **4***S. flaviceps* Macquart (♂).

#### Other material.

Angola • 1♀; Congulu, Apr. 1934; K. Jordan leg.; NHMUK • 1♂; 7 mi W Gabela; 16–18 Mar. 1972; NHMUK. Benin • 1♀; Ahozon; Jun. 2005; G. Goergen leg.; IITA • 1♂; Calavi, IITA Station; 4 Apr. 1994; G. Goergen leg.; IITA • 1♀; same collection data as for preceding; 4 Aug. 1995 • 1♀; same collection data as for preceding; 5 Mar. 1997 • 1♂ 1♀; same collection data as for preceding; Jun. 2006 • 1♂; same collection data as for preceding; 18 Apr. 2007 • 4♂♂ 2♀♀; same collection data as for preceding; Jun. 2007 • 1♂; same collection data as for preceding; 16 Nov. 2011; K. Jordaens leg.; KMMA • 11♂♂ 3♀♀; same collection data as for preceding; 8 Dec. 2013 • 1♂; same collection data as for preceding; 16 Dec. 2013 • 3♀♀; Cotonou; 3 Jun. 1914; W.A. Lamborn leg.; NHMUK • 1♂; Cotonou; 24 Sep. 2002; G. Goergen leg.; IITA • 1♀; same collection data as for preceding; May 2006 • 1♀; same collection data as for preceding; 25 Apr. 2012 • 1♀; Dassa; Aug. 2007; G. Goergen leg.; IITA • 1♂; Lama forest; 26 Jul. 1995; G. Goergen leg.; IITA • 1♂; Lokoli; Feb. 2006; G. Goergen leg.; IITA • 1♂; Lokossa; Jan. 2005; G. Goergen leg.; IITA • 2♀♀; same collection data as for preceding; Oct. 2005 • 2♂♂; same collection data as for preceding; Jan. 2006 • 2♂♂; same collection data as for preceding; Mar. 2006 • 1♀; Niaouli; Jun. 2003; G. Goergen leg.; IITA • 1♀; Ouidah; Nov. 2004; G. Goergen leg.; IITA • 2♀♀; same collection data as for preceding; May 2006 • 1♂ 1♀; same collection data as for preceding; 23 Apr. 2009 • 1♂; Pénéssoulou; Jun. 2004; G. Goergen leg.; IITA • 1♂; Porto Novo; Aug. 2006; G. Goergen leg.; IITA • 1♂; Porto Novo; 27 Jan. 2016; K. Jordaens leg.; KMMA • 1♂; Tanougou Waterfalls; 20 Jan. 2020; G. Goergen leg.; IITA • 1♂; Togbin; Dec. 2005; G. Goergen leg.; IITA. Burundi • 1♂; Busoni, Kisenyi; 17 Dec. 1950; F. François leg.; KBIN • 2♂♂ 1♀; Kibira NP, Rwegura; 31 May 2018; E. Sinzinkayo leg.; OBPE • 1♀; same collection data as for preceding; 28 Nov. 2019 • 1♀; Kisenyi; May 1955; F.J. François leg.; KBIN • 1♀; Kitega; 22 Oct. 1950; F. François leg.; KBIN • 1♂; Lake Nyanza; 7 [?] 2013; L. Ndayikeza leg.; OPBE • 1♂; same collection data as for preceding; 23 [?] 2013 • 1♂; Nyakibande, Mumirwa; 27 Jan. 2018; E. Sinzinkayo leg.; OBPE • 1♀; same collection data as for preceding; 16 May 2018 • 1♂; same collection data as for preceding; 24 Apr. 2019 • 2♂♂ 1♀; same collection data as for preceding; 14 Jul. 2019 • 2♀♀; Nyamurembe, Bururi; 7 Mar. 1953; P. Basilewsky leg.; KMMA • 1♀; Rumonge; 1934; A. Lestrade leg.; KMMA • 1♀; Rumonge; 19–20 Jun. 1948; F. François leg.; KBIN • 1♀; same collection data as for preceding; 25 Nov. 1948 • 1♀; same collection data as for preceding; 25 Feb. 1949 • 1♀; same collection data as for preceding; 19 May 1952 • 1♀; Rumonge; 7 Mar. 1953; P. Basilewsky leg.; KMMA • 1♀ Usumbura [= Bujumbura]; G. Pierrard leg.; KMMA. Cameroon • 2♀♀; Bafut Forest, Nguenba; 29 Nov. 1967; de Miré leg.; MHNH • 1♂; Batanga; 24 Jun. 1911; A.I. Good leg.; CNC • 1♀; Ebogo; 9 Jul. 1999; G. Goergen leg.; IITA • 1♀; Kumba-Victoria Rd boundary post; 9 Nov. 1949; H. Oldroyd leg.; NHMUK • 1♀; Lolodorf; 28 Aug. 1915; A.I. Good leg.; CNC • 2♂♂; Mbalmayo; 3 Jul. 1998; G. Goergen leg.; IITA • 1♂; same collection data as for preceding; 2 Aug. 2000 • 1♀; Victoria [= Limbe]; Jun. –Aug. 1917; F.H. Fitz Roy leg.; NHMUK. Democratic republic of the Congo • 1♀; Abok; 1927; Ch. Scops leg.; KMMA • 2♀♀; Albertville [= Kalemie]; 14 Aug. 1953; J. Verbeke leg.; KBIN • 1♂; Bambesa; 30 Oct. 1933; J.V. Leroy leg.; KMMA • 1♂; Bambesa; 6 Jul. 1937; J. Vrydagh leg.; CNC • 1♀; same collection data as for preceding; 2 Oct. 1937; KMMA • 1♀; same collection data as for preceding; 24 Mar. 1939; KBIN • 1♀; same collection data as for preceding; 3 Jun. 1939; KBIN • 1♀; same collection data as for preceding; 11 Nov. 1940; KMMA • 1♀; Barumbu; Aug. 1925; J. Ghesquière leg.; KMMA • 1♂; Bafwasende; Nov. 1945; F. François leg.; KMMA • 1♂; Bezali; 3 Oct. 1968; P.M. Elsen leg.; KMMA • 1♀; Boma; R.F. Achille leg.; KMMA • 1♀; Boma; 17 Jun. 1915; Lang and Chapin leg.; KMMA • 1♀; Bomputu, Salonga; Jun. 1936; J. Ghesquière leg.; KBIN • 1♂; Bukama; 2 Apr. 1911; Bequaert leg.; KMMA • 1♀; Bumba; Dec. 1939–Jan. 1940; H. De Saeger leg.; KMMA • 1♀; Coquilhatville [= Mbandaka]; 1946; Ch. Scops leg.; KMMA • 1♂; between Deti and Ibambi; 5 Jul. 1931; F.R. Swift leg.; NHMUK • 1♀; Eala; Mar. 1935; A. Corbisier leg.; KMMA • 1♀; Eala; Mar. 1935; J. Ghesquière leg.; KBIN • 1♀; same collection data as for preceding; 12 Aug. 1935 • 1♀; same collection data as for preceding; 19 Nov. 1935 • 1♂; same collection data as for preceding; 12 Nov. 1936 • 1♀; Elisabethville [= Lubumbashi]; Nov. 1932; C.V. Hirschberg leg.; KBIN • 1♀; Elisabethville [= Lubumbashi]; Nov. 1933; M. Bequaert leg.; KBIN • 1♂; same collection data as for preceding; 22 Apr. 1934 • 1♀; same collection data as for preceding; 3 Mar. 1935 • 1♂; Elisabethville [= Lubumbashi]; 1–6 Sep. 1932; de Loose leg.; KMMA • 1♀; same collection data as for preceding; 24 Nov. 1932 • 1♀; Faradje; 30 Mar. 1912; Lang and Chapin leg.; KMMA • 1♀; same collection data as for preceding; Nov. 1912 • 1♀; Idjwi Island, Kivu; 8 Nov. 1952; J. Verbeke leg.; KBIN • 1♀; Jadotville [= Likasi]; J. Muller leg.; KBIN • 3♂♂; Kadjudju; Feb. 1932; G. Babault leg.; MHNH • 4♂♂ 4♀♀; same collection data as for preceding; May–Jun. 1932 • 2♂♂ 8♀♀; same collection data as for preceding; Aug. 1932 • 2♂♂; same collection data as for preceding; Sep. 1932 • 1♀; Kapanga; Nov. 1932; F.G. Overlaet leg.; KMMA • 2♂♂ 1♀; same collection data as for preceding; Dec. 1932 • 1♀; same collection data as for preceding; Mar. 1933 • 1♀; Kasongo; 10 Sep. 1959; P.L.G. Benoit leg.; KMMA • 1♀; Katoko-Kobe; 10–20 Jan. 1958; Segers leg.; KBIN • 1♀; Katompe; 12 Dec. 1923; M. Bequaert leg.; KMMA • 1♀; Kimuenza; Sep. 1962; M.J. Deheegher leg.; KMMA • 1♀; Kisantu; 1927; P. Vanderyst leg.; KMMA • 1♀; Komi, Sankuru; Dec. 1930; J. Ghesquière leg.; KMMA • 1♀; same collection data as for preceding; CNC • 2♂♂; Kwango; Panzi; 12 Feb. 1939; Mrs Bequaert leg.; KMMA • 1♀; Lubutu; 22 Jan. 1915; J. Bequaert leg.; KMMA • 1♀; Lukolela; 3 Jan. 1931; .P. Chapin leg.; AMNH • 1♂; same collection data as for preceding; 7 Jan. 1931 • 1♀; Luputa, Lomami; Jan. 1935; Bouvier leg.; KMMA • 1♀; same collection data as for preceding; Mar. 1935 • 1♂; same collection data as for preceding; May 1935 • 1♀; Mbandaka; 1 Feb. 1962; A.B. Stam leg.; KMMA • 1♀; Mboga; Jul. 1914; J. Bequaert leg.; KMMA • 1♀; Medje, Ituri; 9–15 Jul. 1910; Lang and Chapin leg.; AMNH • 1♂; Medje, Ituri; 1–7 Sep. 1910; Lang and Chapin leg.; KMMA • 1♂ 2♀♀; Moma, Equateur; Jun. 1925; J. Ghesquière leg.; KMMA • 1♀; N’Guli; 6–9 Oct. 1913; Rodhain leg.; KMMA • 1♀; Niangara, Uelé; Nov. 1910; Lang and Chapin leg.; KMMA • 1♀; Nzali, Ubangi; 3 Feb. 1932; H.J. Brédo leg.; KMMA • 2♀♀; Pawa, Uelé; 1938; A. Dubois leg.; KMMA • 1♀; Poko, Uelé; Aug. 1913; Lang and Chapin leg.; AMNH • 1♀; same collection data as for preceding; KMMA • 1♂; Pweto, Katanga; Oct. 1926; A. Bayet leg.; KMMA • 3♂♂ 1♀; Stanleyville [= Kisangani]; Mar. 1915; Lang and Chapin leg.; AMNH • 1♂; same collection data as for preceding; CNC • 3♂♂ 1♀; same collection data as for preceding; KMMA • 1♀; same collection data as for preceding; 6 Apr. 1915; CNC • 1♀; same collection data as for preceding; 7 Apr. 1915; KMMA • 1♀; same collection data as for preceding; 14 Apr. 1915 • 1♂; same collection data as for preceding; Apr. 1915 • 1♂; same collection data as for preceding; KBIN • 1♀; Tshibamba, Lulua; Mar. 1932; F.G. Overlaet leg.; KMMA • 4♀♀; Uvira, Kivu; 25 Aug. 1957; P. Basilewsky leg.; KMMA. Equatorial Guinea • 1♂; Fernando Po [= Bioko Island], Basile; Aug. 1901; L. Fea leg.; MCSNG • 1♂; Musola; Jan. 1902 ; L. Fea leg.; MCSNG • 1♀; same collection data as for preceding; Mar. 1902 • 1♀; Punta Frailes; Oct.–Nov. 1901; L. Fea leg.; MCSNG • 1♂ 1♀; San Benito [= Mbini]; 1885; Guiral leg.; MHNH. Ethiopia • 1♂; Arba-Minch, Lake Chano; 19 Oct. 2012; A. Pauly leg.; KMMA. Gabon • 1♂; Ipassa, route de Makokou; 2–17 May 1974; M. Donskoff and J. Le Breton leg.; MHNH. Ghana • 1♀; Accra; 1–20 Jul. 1988; M. Hauser leg.; CDFA • 1♀; Buroburo, Kumasi; 4 Jun. 1976; A.B. Stam leg.; KMMA • 1♀; same collection data as for preceding; 12 Jul. 1976 • 1♀; same collection data as for preceding; 17 Jul. 1976 • 1♀; same collection data as for preceding; 28 Jul. 1976 • 1♀; Cape Coast; Dec. 2004; G. Goergen leg.; IITA • 1♂; Obuasi, Ashanti; 8 Aug. 1906; W.M. Graham leg.; NHMUK • 2♂♂ 1♀; same collection data as for preceding; 25 Aug. 1907 • 1♀; Takoradi; 2–6 Jun. 1957; G.H. Heinrich leg.; CNC • 1♂; Tsito; 26 Jan. 1969; O.W. Richards leg.; NHMUK • 2♂♂ 1♀; Wati Waterfalls; Feb. 2003; G. Goergen leg.; IITA • 1♀; Worawora; Mar. 2005; G. Goergen leg.; IITA. Guinea Bissau • 1♂ 2♀♀; Bolama; Jun.–Dec. 1899; L. Fea leg.; MCSNG. ‘GUINEA’ • 1♀; Westermann leg.; MNB [labeled as type of *maculipennis* considered to originate from Guinea Bissau according Smith and Vockeroth (1980); cf. comments]. Ivory Coast • 1♀; Adiopodoumé; 4 Jul. 1948; Jouer leg.; MHNH • 1♂; Banco National Park; 23 Apr. 1989; J. Londt leg.; NMSA. Kenya • 1♀; Itieni Forest, Nyambene Hills; 16–30 Oct. 2011; R.S. Copeland leg.; ICIPE • 1♂; Kaimosi; Feb. 1949; V.G.L. van Someren leg.; NHMUK • 1♀; Lukusi River, Kakamega Forest; 5 Apr.–26 Jun. 2017; R.S. Copeland leg.; ICIPE • 1♂; Taveta Forest; Aug. 1947; V.G.L. van Someren leg.; NHMUK. Liberia • 1♂; Bendu, Robertsport; 11 Apr. 1943; F.M. Snyder leg.; AMNH • 1♀; Du River camp 3; 1926; J. Bequaert leg.; KMMA • 1♀; Kpaine; 17 Aug. 1953; W. Peters leg.; NHMUK • 1♂; Monrovia; 24 May 1957; G.H. Heinrich leg.; CNC. Malawi • 1♂; Mt Mlanje; 9 Oct. 1913; S.A. Neave leg.; NHMUK. Mozambique • 1♀; East of Mt Mlanje; 21 Nov. 1913; S.A. Neave leg.; NHMUK • 1♂; Kola Valley; 5 Apr. 1913; S.A. Neave leg.; NHMUK • 1♂; Rikatla; H.A. Junod leg.; NMSA. Nigeria • 1♂ 1♀; Afikpo; 2 Jun. 1910; J.J. Simpson leg.; NHMUK • 1♂; Gadau; Jul. 1938; Buxton and Lewis leg.; NHMUK • 2♂♂; Ibadan; 24 Jul. 1947; J.L. Gregory leg.; NHMUK • 1♀; same collection data as for preceding; Jun. 1951; CNC • 1♂; Ibadan, IITA station; 24 Jan. 1997; G. Goergen leg.; IITA • 1♀; same collection data as for preceding; 26 Jan. 1998 • 1♂ 1♀; Ile-Ife; 30 Oct. 1969; J.T. Medler leg.; CNC • 1♀; Jos; Oct.–Dec. 1965; E. Bot Gwong leg.; KMMA • 1♀; Kaduna; Aug. 1952; Nash leg.; NMSA • 2♀♀; Kaduna; 19 Sep. 1962; M.W. Service leg.; NHMUK • 1♀; Olokemeji, Ibadan; AMNH. Republic of the Congo • 1♀; Fernand-Vaz; Sep.–Oct. 1902; L. Fea leg.; MCSNG • 1♂; Haut Ivindu, Ogooué; 1906; J. Gravot leg.; MHNH • 1♂; Lambaréné, Ogooué; 1913; E. Ellenberger leg.; MHNH. Sierra Leone • 1♀; Bomaru; 8 Aug. 1912; J.J. Simpson leg.; NHMUK • 1♀; Daru; 31 Jul. 1912; J.J. Simpson leg.; NHMUK • 1♀; same collection data as for preceding; 18 Aug. 1912 • 1♀; Ka-Yima; 23 Jun. 1912; J.J. Simpson leg.; NHMUK • 1♀; Lungi; 14 Jul. 1959; C.P. Hoyt leg.; NHMUK. South Africa • 1♂; Dukuduku, between St Lucia and Matubatuba; 7–8 Apr. 1960; B. Stuckenberg leg.; NMSA • 1♀; Empangeni; 15 Apr. 2003; P. Reavell leg.; NMSA • 1♀; False Bay Park Reserve, Mpophomeni Trail area; 2 Jan. 1996; J. and A. Londt leg.; NMSA • 1♂ 1♀; Hazyview, Kruger Park Lodge area; 1–7 Oct. 2010; J. and A. Londt leg.; NMSA • 1♀; Middlekop; 11 Mar. 1979; NMSA • 1♂; Mpenjati Nature Reserve; 20 Mar. 2010; J. and A. Londt leg.; NMSA • 2♂♂ 1♀; same collection data as for preceding; 22–23 Jan. 2011 • 1♂; San Lameer Resort area; 11 Feb. 2012; J. and A. Londt leg.; NMSA. Tanzania • 1♀; Amani; G. Pringle leg.; NHMUK • 1♂; Kibonoto, Kilimanjaro; 29 Apr. 1906; Sjöstedt leg.; MNB • 1♀; Kigoma; 1924; C.R. Steel leg.; NHMUK • 1♂; Lembene, Usambara; 7 Jun. 1916; T.J. Anderson leg.; NHMUK • 3♀♀; Morogoro; J.G. Halcrow leg.; NHMUK • 1♂ 1♀; Morogoro; 16 May 1925; A.H. Ritchie leg.; NHMUK • 1♂ 1♀; same collection data as for preceding; 18 May 1925 • 1♂ 2♀♀; same collection data as for preceding; 20 May 1925 • 2♂♂ 1♀; same collection data as for preceding; 28 Jun. 1925 • 1♀; same collection data as for preceding; 10 Jun. 1925 • 1♀; Morogoro, Uluguru; A.G. Wilkins leg.; NHMUK • 1♀; Moschi; 1904; Alluaud leg.; MHNH. Togo • 1♀; Dzogbégan; 3 Jul. 1997; G. Goergen leg.; IITA • 1♂; Dzogbégan; 24–25 Jan. 2016; K. Jordaens leg.; KMMA • 1♂ 1♀; Kloto; 5 May 1996; G. Goergen leg.; IITA • 1♀; same collection data as for preceding; 3 Jul. 1997 • 1♂; same collection data as for preceding; Mar. 2000 • 6♂♂ 1♀; same collection data as for preceding; Aug. 2003 • 13♂♂ 5♀♀; same collection data as for preceding; Jan. 2004 • 3♂♂; same collection data as for preceding; Feb. 2004 • 31♂♂ 3♀♀; same collection data as for preceding; Mar. 2004 • 5♂♂ 1♀; same collection data as for preceding; Apr. 2004 • 1♂ 1♀; same collection data as for preceding; Jun. 2004 • 2♂♂; same collection data as for preceding; Nov. 2004 • 1♂ 1♀; same collection data as for preceding; Jan. 2005 • 4♂♂; same collection data as for preceding; Feb. 2005 • 3♂♂; same collection data as for preceding; Mar. 2005 • 1♀; same collection data as for preceding; Sep. 2005 • 1♀; same collection data as for preceding; Nov. 2005 • 1♂; same collection data as for preceding; Dec. 2005 • 1♂; same collection data as for preceding; Feb. 2006 • 1♀; same collection data as for preceding; May 2007 • 1♂; same collection data as for preceding; Jun. 2007 • 2♂♂; same collection data as for preceding; Oct. 2007 • 1♂; same collection data as for preceding; Jan. 2008 • 1♀; same collection data as for preceding; Jul. 2008 • 1♂; same collection data as for preceding; Jul. 2015 • 1♀; Kloto Forest; Jun. 2004; G. Goergen leg.; IITA • 1♂; Kuma-Tokpli; 21–24 Jan. 2016; K. Jordaens leg.; KMMA • 1♂; Lokossa; Jun. 2006; G. Goergen leg.; IITA • 1♀; Obé, near Akloa; 22 Apr. 2008; A. Ssymank leg.; MZH. Uganda • 1♀; Ankole; H.B. Johnston leg.; NHMUK • 1♂; Southeast Ankole; 4–8 Oct. 1911; S.A. Neave leg.; NHMUK • 1♂; Western Ankole; 10–14 Oct. 1911; S.A. Neave leg.; NHMUK • 1♀; between Jinja and Busia or Mbwago, E. Busoga; 28 Jul.–1 Aug. 1911; S.A. Neave leg.; NHMUK • 2♀♀; between Lake Kioga SE shore and Kakindu; 22–23 Aug. 1911; S.A. Neave leg.; NHMUK • 1♂; Buamba Forest, Semliki Valley; 3–7 Nov. 1911; S.A. Neave leg.; NHMUK • 3♂♂ 4♀♀; Budongo Forest, nr Lake Albert; Apr. 1972; E. Babyetagara leg.; CNC • 1♂ 1♀; Busoga; 1903; D. Bruce leg.; NHMUK • 2♂♂; Busoga; Mar. 1906; A. Hodges leg.; NHMUK • 1♂; Entebbe; 12–20 Jan. 1912; S.A. Neave leg.; NHMUK • 3♀♀; Entebbe; 14 Nov. 1912; C.C. Gowdey leg.; NHMUK • 1♂ 1♀; same collection data as for preceding; 18–20 Nov. 1912 • 1♀; same collection data as for preceding; 3–4 Dec. 1912 • 1♂; same collection data as for preceding; 12–13 Dec. 1912 • 1♀; Entebbe; 1 Feb. 1972; H. Falke leg.; CNC • 1♂; same collection data as for preceding; 10 Jun. 1972 • 1♂; same collection data as for preceding; 23–31 Jan. 1973 • 3♂♂; same collection data as for preceding; 25–28 Jan. 1973 • 1♀; same collection data as for preceding; Mar. 1973 • 1♀; Entebbe; 1 May 1973; M.K. Paulus leg.; CNC • 1♂; nr Entebbe; 23–31 Jan. 1973; H. Falke leg.; CNC • 1♂ 1♀; same collection data as for preceding; 1–14 Feb. 1973 • 1♀; south side Lake George; 17–19 Oct. 1911; S.A. Neave leg.; NHMUK • 4♂♂; Ibanda; 23–28 Dec. 1972; H. Falke leg.; CNC • 1♀; Jinja; Sep. 1928; V.G.L. van Someren leg.; NHMUK • 1♀; near Kakindu, Nile banks; 24 Aug. 1911; S.A. Neave leg.; NHMUK • 3♂♂ 2♀♀; Kayonza Forest, Kigezi; Sep. 1972; H. Falke leg.; CNC • 1♂; Kigaloma, Katwe; 2 Feb. 1912; H.B. Owen leg.; NHMUK • 1♀; Kilembe, Ruwenzori Range; Dec. 1934–Jan. 1935; F.W. Edwards leg.; NHMUK • 1♀; Magyo, Buvuma Island, Lake Victoria; Mar. 1968; E. Vertriest leg.; KMMA • 1♀; Masaka, Katera Forest; Nov. 1955; V.G.L. van Someren leg.; NHMUK • 1♀; Mbale-Kimu Road; 15–17 Aug. 1911; S.A. Neave leg.; NHMUK • 1♀; Mt Kokanjero; 7–9 Aug. 1911; S.A. Neave leg.; NHMUK • 1♀; W. Nile; 13 Nov. 1958 NHMUK • 2♂♂; Nkokonjera; 23 Dec. 1910; C.C. Gowdey leg.; NHMUK • 1♂; Nyabushozi, Nyrbthozi County; 21 Jan. 1975; P. Mugabi leg.; CNC • 1♀; Salt Lake to Wawamba Country; 1894; G.F. Scott Elliot leg.; NHMUK • 1♂ 1♀; Siroko River; 12–14 Aug. 1911; S.A. Neave leg.; NHMUK • 4♂♂ Tero Forest, Southeast Buddu; 26–30 Sep. 1911; S.A. Neave leg.; NHMUK • 1♂; Unyoro, Kafu River Valley; 23–28 Dec. 1911; S.A. Neave leg.; NHMUK • 1♂; Wasa River; 11–14 Apr. 1914; R.E. McConnell leg.; NHMUK. Zimbabwe • 1♀; Bomponi; 6 May 1963; D. Cookson leg.; NMSA.

**Figures 5, 6.**
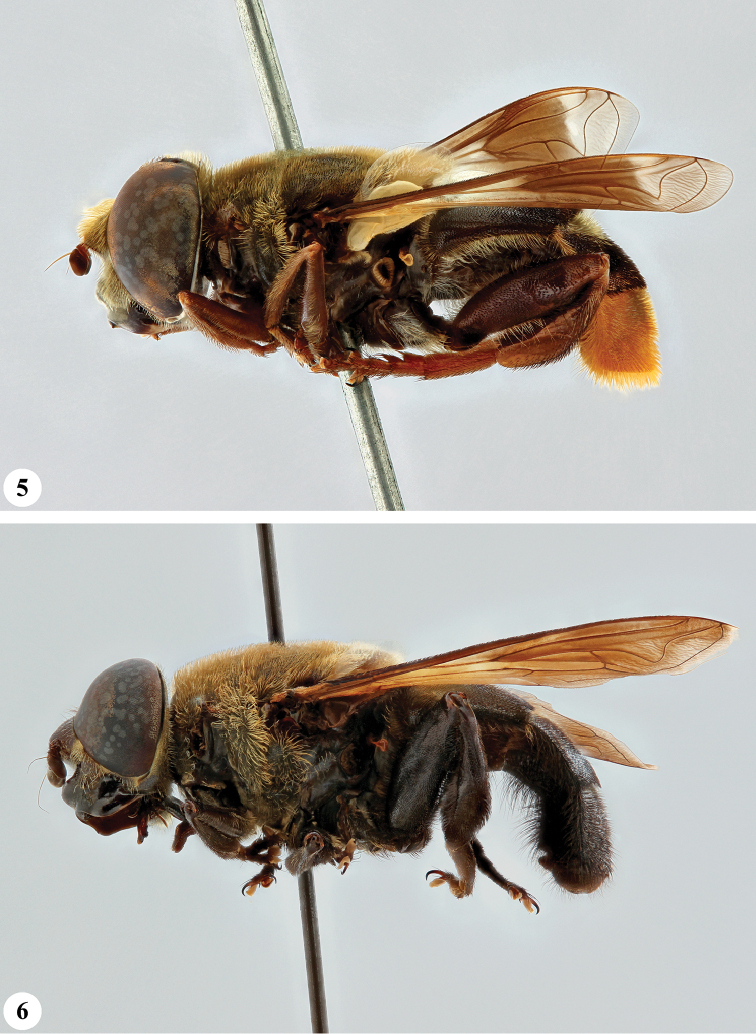
*Senaspis* species, habitus, lateral view **5***S. haemorrhoa* (Gerstaecker) (♂) **6***S. melanthysana* (Speiser) (♂).

#### Description.

Body length: 10.3–15.9 mm. Wing length: 7.9–12.0 mm.

**Figures 7, 8.**
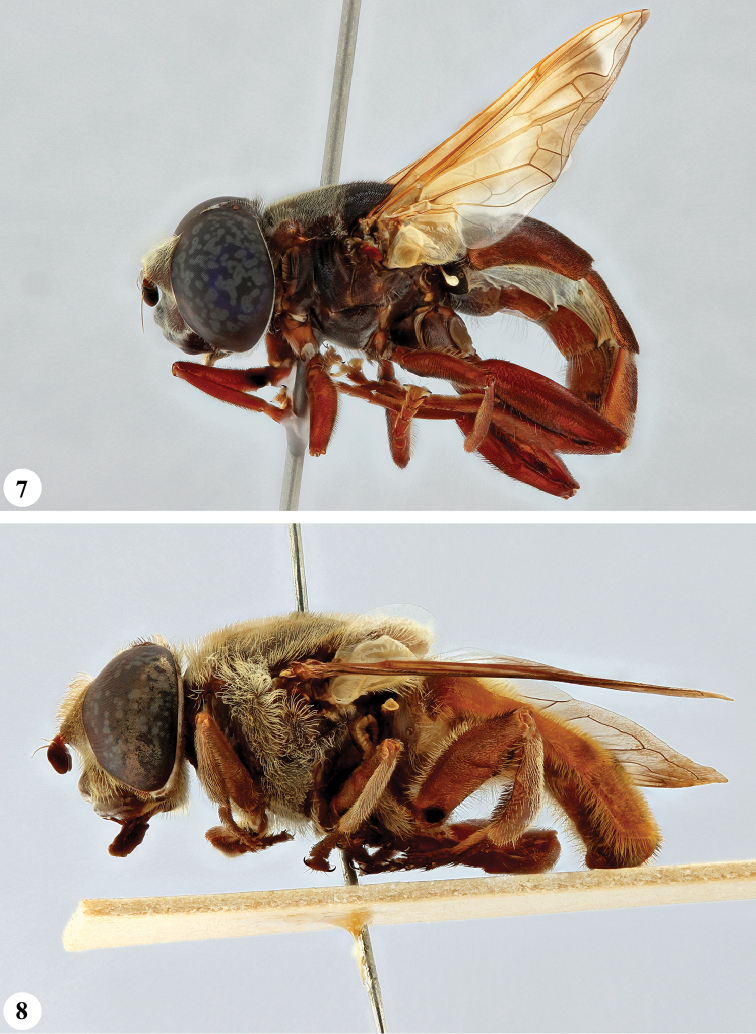
*Senaspis* species, habitus, lateral view **7***S. nigrita* (Bigot) (♂) **8***S. xanthorrhoea* (Bezzi) (♂).

**Male** (Fig. 1). Head (Figs 12, 13). Eye bare; holoptic, eye contiguity for distance equal to length of ocellar triangle, facets dorsally slightly larger, at most twice as large in diameter as ventral ones. Frons black to black-brown; largely subshiny, with greyish brown pollinosity in dorsal fifth and along eye margins; with very dispersed, short pale pile. Face black to black-brown; subshiny with pale brownish to greyish pollinosity, in parts more densely so, medial part and ventral margins largely devoid of pollinosity; in parts with dispersed long pale pile; facial tubercle strongly pronounced. Gena colour and pollinosity as ventral lateral margins of face, with short to long pale pile. Occiput black-brown, covered with dull grey pollinosity; with dispersed pale pile. Antennal segments black-brown, basoflagellomere apically slightly paler; arista pale to dark yellow.

**Figures 9, 10.**
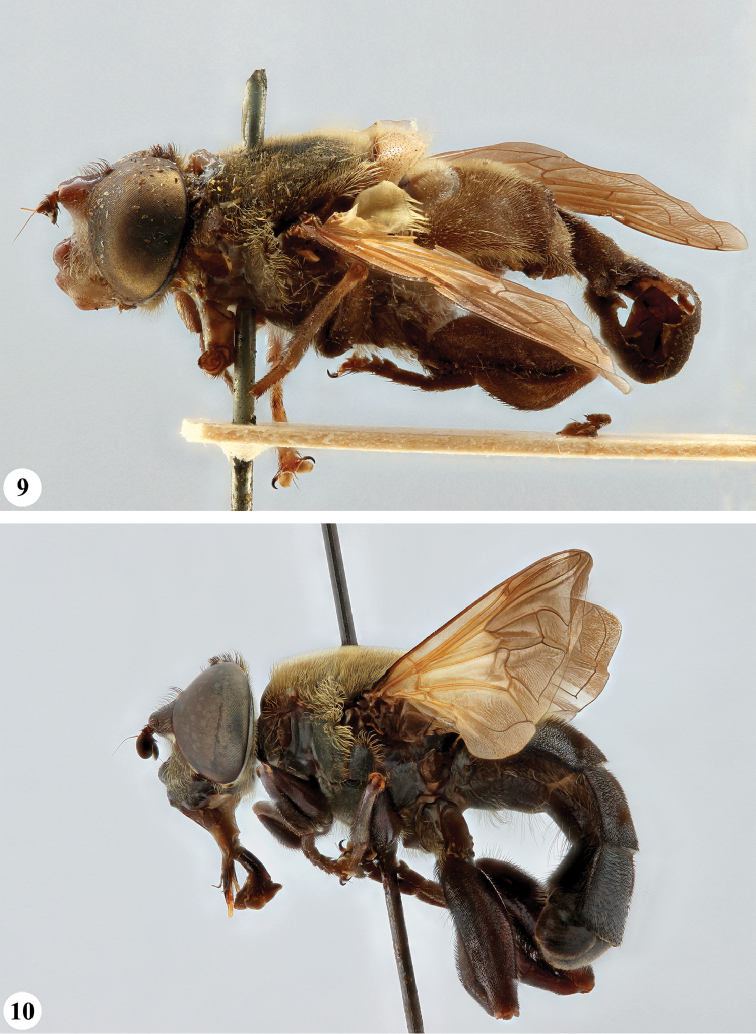
*Senaspis* species, habitus, lateral view **9***S. umbrifera* (Walker) (♂ holotype) **10**S. nr umbrifera (Walker) (♂).

***Thorax*** (Fig. 52). Scutum subshiny black; grey pollinose, except on anterior part of scutellum, at transverse suture and along lateral margins where more densely orange-brown pollinose; with short pale pile, anteriorly and on notopleuron somewhat longer. Scutellum pale brownish to orange-brown, anterior margin narrowly darker; with short pale pile. Pleura ground colour black-brown, sparsely greyish pollinose; covered with dispersed long pale pile, anterior anepimeron sometimes partially black pilose; pilosity absent on meron, dorsomedial anepimeron, anterior part of katepisternum and anterior anepisternum. Scutellum apical margin weakly rounded, distinctly marginated, 2.5 times as wide as long.

***Legs***. Brown to black-brown; with short black pile, along posterior margin of pro- and mesofemora with longer pale pile. Metafemur (Fig. 80) distinctly thickened, with one distinct ventral swelling in apical fifth, with dense short stout setae on swelling; metatibia thickened and slightly curved, with pile along ventral margin not distinctly more dense.

***Wing*** (Fig. 64). Faint yellowish brown tinge, towards posterior margin and apex more greyish; with distinct dark brown macula running from anterior margin and covering most of stigma, parts of cell r_1_ and r_2+3_, distal part of cell br and basal part of r_4+5_, decreasing in colour on anterior part of cell dm; distally of brown macula more hyaline patch. Calypters yellow-white, in medial part more pale brownish; with fringe of yellow-white pile. Cell r_1_ variable, usually closed with petiole at most equal to height of base of stigma but usually shorter, sometimes closed but petiole missing; vein R_4+5_ sinuate, usually not appendiculate, rarely with short appendix.

***Abdomen*** (Fig. 91). Subshiny brown to black-brown, postabdomen more reddish brown; with greyish to greyish brown pollinosity, except for anteromedial macula on terga I and II, pair of sublateral maculae on terga I–III and posterior part of tergum IV where more shining; with short dark pile, except tergum I, anterior margin tergum II, and lateral margins of all terga where pale pile. Sterna orange-brown to black-brown, with short dispersed pale pile. Male genitalia as in Fig. 102.

**Female.** As male except for the following character states: Eye (Figs 14, 15) dichoptic, facets equal to subequal in size. Frons subshiny black to black-brown in ventral protruding part, dorsally with greyish to greyish brown pollinosity. Postabdominal terga often less reddish brown; subshiny maculae sometimes more extensive and more pronounced.

#### Distribution.

Angola, Benin, Burundi, Cameroon, Democratic Republic of the Congo, Equatorial Guinea, Ethiopia, Gabon, Ghana, Guinea Bissau, Ivory Coast, Kenya, Liberia, Malawi, Mozambique, Nigeria, Republic of the Congo, Sierra Leone, South Africa, Tanzania, Togo, Uganda, Zimbabwe. ‘Java’ as type locality is probably an error.

#### Comments.

The orginal description of *dentipes* Macquart mentioned the geographic origin of the type as “de Java”. No label on the lectotype pin indicates a locality or region, and the locality is probably an error. The type specimen of *dentipes* bears a lectotype label with designation by F.C. Thompson in 1977. The original description did not indicate the number of specimens examined by Macquart but only made reference to the fact that the description is based on male only and that material is housed in the ‘Muséum’ (referring to MNHN). No additional specimens could be traced in the MNHN collection. The lectotype designation (made in accordance with recommendation 73♀ of the ICZN (Thompson pers. comm.)) has not been published elsewhere.

The type of *maculipennis* Loew could not be traced. According to the original description (Loew 1858) as well as the detailed redescription by Loew (1860), this is a male specimen from ‘Guinea’. One female specimen in MNB collection (Guinea, Westerm., additional label ‘843’) is indicated as type but this contradicts with the above, and thus appears not be part of the type series. This specimen was also mentioned by Karsch (1887). According to Smith and Vockeroth (1980) ‘Guinea’ refers to Guinea Bissau.

The taxon *livida* Bezzi was described as a variety of *aesacus* Walker by Bezzi (1912), based on two specimens from Fernando Po [= Bioko Island], which were among a series of specimens identified as *aesacus*. He pointed out the few morphological differences and suggested that they could be just teneral specimens, which did not attain their full colours and wing markings. Smith and Vockeroth (1980) elevated *livida* to species rank. Examination of the type material, however, indicates that Bezzi was correct in considering these as teneral specimens and that there is no morphological evidence for considering *livida* as a distinct species, different from *dentipes*. We, therefore, synonymize *livida* with *dentipes*.

**Figure 11.**
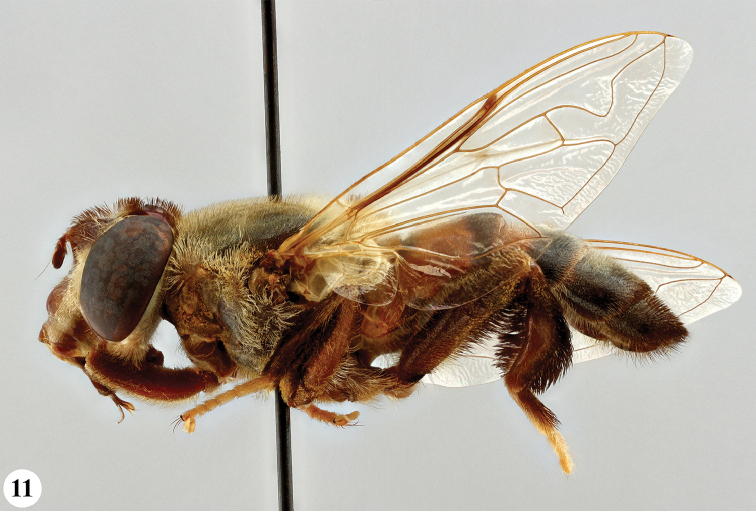
Habitus, lateral view *S. pennata* (Hervé-Bazin) (♀).

### Senaspis dibapha

Taxon classificationAnimaliaDipteraSyrphidae

(Walker, 1849)

1FDCC411-F702-54A6-9CB0-DD66DD81AE4F

Figs 2 , 16–19 , 53 , 65–67 , 81 , 92 , 103

Xylota dibaphus Walker, 1849: 560.
Eristalis nigripennis Macquart, 1855: 108. Syn. nov.
Eristalomyia rufonasuta Bigot, 1891: 375. Syn. by Bezzi (1912).
Eristalis (Stenaspis) gypseisquama Speiser, 1910: 123. Syn. by Bezzi (1912).
Eristalis (Stenapsis) gypseisquama var. sulfurata Speiser, 1911: 240. Syn. by Smith and Vockeroth (1980).

#### Differential diagnosis.

The only *Senaspis* species with a pair of lateral facial tubercles in addition to the medial one (Fig. 16) (only single medial tubercle in all other *Senaspis* species). Legs predominantly orange to rufous with slender metafemur (Fig. 81) (thickened in all other *Senaspis* species). The wing is largely brownish without distinct medial macula (Fig. 65).

**Figures 12–15.**
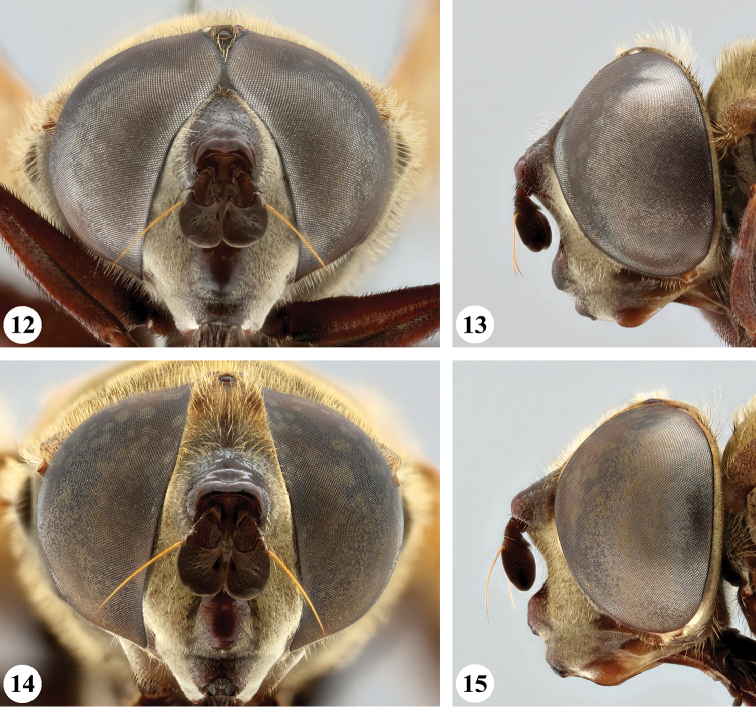
Head, frontal and lateral view *S. dentipes* (Macquart) (**12, 13** ♂ **14, 15** ♀).

#### Examined material.

*Xylota dibaphus* Walker: Lectotype (hereby formally designated and published; see comments), female, “Lecto- // type” “Xylota // Type // dibaphus // Walk.” “Locality?” “LECTOTYPE of // Xylota // dibaphus Walker // desig. F.C. Thompson 1987” [NHMUK].

**Figures 16–19.**
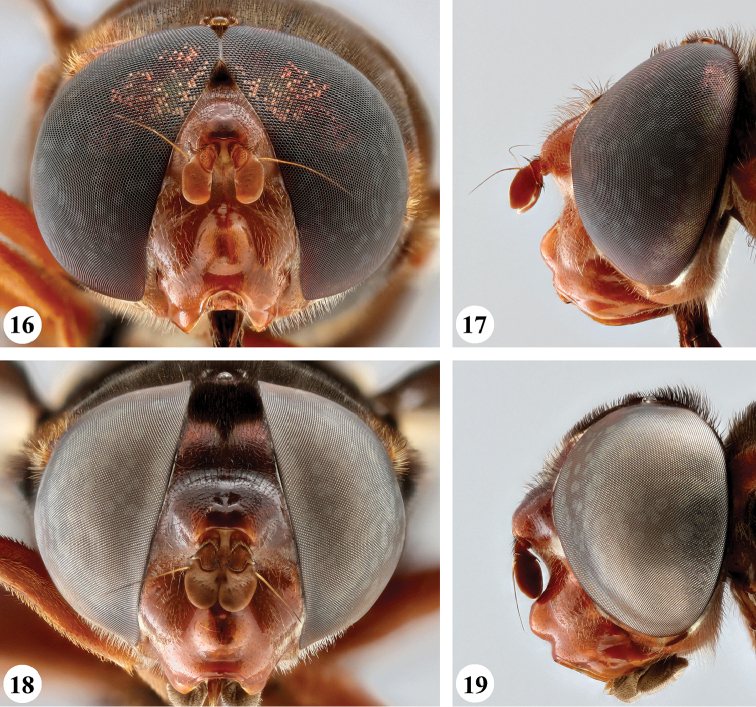
Head, frontal and lateral view *S. dibapha* (Walker) (**16, 17** ♂ **18, 19** ♀).

*Eristalis nigripennis* Macquart: Lectotype (hereby designated), male, “SYN- // TYPE” “ex. coll. Bigot. // Prs. by // G.H. Verrall. // B.M.1901–14.” “E. nigripennis ♂. // Columbia Macq.” “NHMUK010369880” “LECTOTYPUS” [NHMUK]. Paralectotype, male, “SYN- // TYPE” “COLUMBIA” “ex. coll. Bigot. // Prs. by // G.H. Verrall. // B.M.1901–14.” “Eristalis // nigripennis // ♂. Macq.” “NHMUK010369879” “PARA- // LECTOTYPUS” [NHMUK].

*Eristalomyia rufonasuta* Bigot: Holotype, female, “Holo- // type” “Assinie // Afrique oc” “Type // J. Bigot” “Eristalomyia // rufonasuta // ♀” “Assinie, // W. Africa. // Ex coll. // Bigot. // Pres by // G.H. Verrall. // 94.234.” “BMNH(E) # // 230784” [NHMUK].

Eristalis (Stenaspis) gypseisquama Speiser: Holotype, female, “Kilimandj. // Sjöstedt” “Kibonoto // kulturz.” “maj” “Eristalis (Dolichom.) // gÿpseisquama // P. Speiser det. // Tÿpe!” “NHRS-BYWS // 000002768” “Loan // 575/99” [NRMS, examined through images].

Eristalis (Stenapsis) gypseisquama var. sulfurata Speiser: Lectotype (hereby designated), male, “Typus” “Kamerun // Barombi-Stat. // Preuss S.” “Juli–Oktob. // 1890 Preuss S.” “Zool. Mus // Berlin” “LECTOTYPUS” [MNB]. Paralectotype, female, “Typus” “Kamerun // Barombi-Stat. // Preuss S.” “Zool. Mus // Berlin” “PARA- // LECTOTYPUS” [MNB]. Paralectotype, female, “Typus” “Kamerun // Barombi-Stat. // Preuss S.” “Zool. Mus // Berlin” “PARA- // LECTOTYPUS” [MNB]. Paralectotype, male, “Typus” “S. Kamerun. // Bipindi //IV. 97. //G. Zenker. S°.” “Buschwald. // IV. 97.” “Eristalis (Stenasp) // gypseisquama m. // P. Speiser det.” “Zool. Mus. // Berlin ” “PARA- // LECTOTYPUS ” [MNB]. Possibly paralectotype (see comments), female: “Sierra Leone // Dr Staudinger V. ” “Sierra // Leone // Staudinger” “Zool. Mus. // Berlin ” [MNB].

**Figures 20–23.**
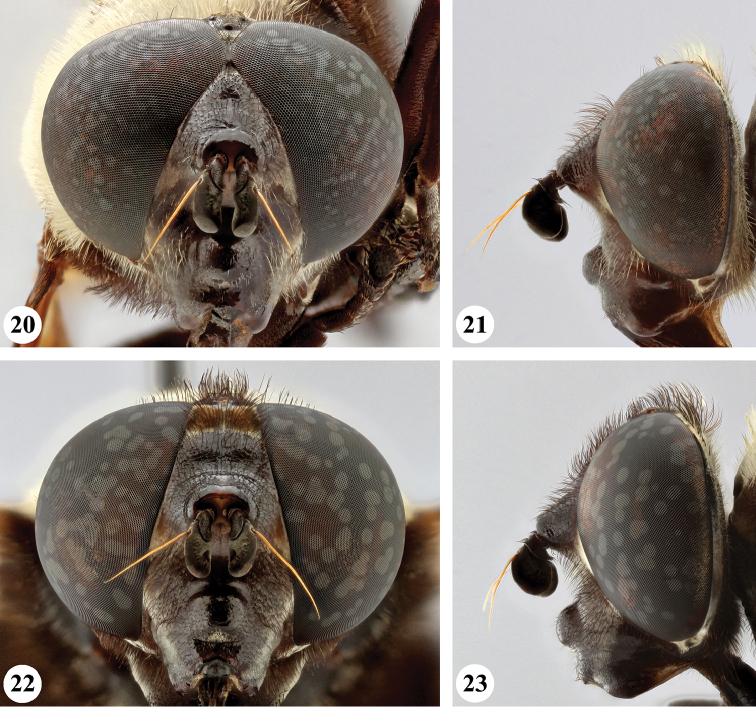
Head, frontal and lateral view *S. elliotii* Austen (**20, 21** ♂ **22, 23** ♀).

#### Other material.

Angola • 1♂; Camissombo; 4–13 Feb. 1958; G.H. Heinrich leg.; CNC • 1♀; Landana; 1875; Klein leg.; MNHN • 2♂♂ 1♀; 30 km N of Quiculungo; Sep.–Oct. 1957; CNC • 1♀; Texeira de Sousa [= Luau]; Feb. 1965; C.A. Green leg.; NMSA. Benin • 1♀; Bonou; 24 Nov. 2011; G. Goergen leg.; IITA • 3♂♂ 3♀♀; Niaouli; 21 Nov. 2011; G. Goergen leg.; IITA • 1♂; Niaouli; 10 Dec. 2013; K. Jordaens and G. Goergen leg.; KMMA • 3♀♀; Pénéssoulou; Jun. 2014; G. Goergen leg.; IITA • 1♀; Pobè; 27 Jan. 2016; G. Goergen leg.; IITA • 1♂ 9♀♀; Porto Novo; Aug. 2006; G. Goergen leg.; IITA • 1♂; Sérou; Nov. 2016; G. Goergen leg.; IITA. Burundi • 1♀; Bubanza, Gihanga Hill; 20 Nov. 195[?]; F.J. François leg.; KBIN • 1♀; Kisenyi; 26 Dec. 1952; KBIN • 1♀; Mugina; 21 Jun. 1949; F.J. François leg.; KBIN • 3♂♂ 2♀♀; Murmirwa, Nyakibande; 24 Apr. 2019; E. Sinzinkayo leg.; OBPE • 3♂♂ 1♀; same collection data as for preceding; 14 Jul. 2019 • 1♂; Murmirwa, Nyambuye; 18 Jun. 2019; E. Sinzinkayo leg.; OBPE • 3♀♀; same collection data as for preceding; 18 Sep. 2019 • 4♂♂ 6♀♀; Rwegura, Kibira NP; 31 May 2018; E. Sinzinkayo leg.; OBPE • 1♀; Teza, Kibira NP; 28 May 2018; E. Sinzinkayo leg.; OBPE. Cameroon • 2♀♀; Abong M’bang; 1–30 Apr. 1936; F.G. Merfield leg.; NHMUK • 1♂ 1♀; D’Ja Posten; 1–30 Jul. 1936; F.G. Merfield leg.; NHMUK • 1♂; Lolodorf; 5 Nov. 1913; A.I. Good leg.; CNC • 3♀♀; Mbalmayo; 3 Jul. 1998; G. Goergen leg.; IITA • 2♀♀; same collection data as for preceding; 7 Jul. 1998 • 5♀♀; same collection data as for preceding; 8 Jul. 1998 • 3♀♀; same collection data as for preceding; Sep. 1998 • 1♀; same collection data as for preceding; Nov. 1998 • 1♂; same collection data as for preceding; May 1999 • 1♀; same collection data as for preceding; 9 Jul. 1999 • 1♀; Metet; 15 Aug. 1919; A.I. Good leg.; CNC • 1♂; N’Tem; 1907; Cottes leg.; MNHN • 1♀; Tokélé; 16 Oct. 1965; B. de Miré leg.; MNHN • 1♀; Yaoundé-Ototomo; 23 Jul. 1965; L. Matile leg.; MNHN • 1♂; Yaoundé-Nkolbisson; 17–21 Oct. 1967; L. Tsacas leg.; MNHN. Central African Republic • 1♂; Bagandou, Lobaye; 14 Sep. 1967; L. Matile leg.; MNHN. Democratic republic of the Congo • 1♀; Abok; Oct. 1935; Ch. Scops leg.; KMMA • 5♂♂ 12♀♀; Akenge, Stanleyville [= Kisangani]; Lang and Chapin leg.; KMMA • 1♀; Akunimbagi; 22 Nov. 1932; H.J. Brédo leg.; KMMA • 1♀; Bafwasende, Stanleyville [= Kisangani]; Nov. 1945; F. François leg.; KMMA • 1♀; same collection data as for preceding; 3 Mar. 1946 • 1♂ 1♀; Bafuwasikuli; 11 Sep. 1912; Christy leg.; KMMA • 1♀; Bamania, Equateur; 1958; P. Hulstaert leg.; KMMA • 1♀; Bamanya, Tshuapa; 1968; P. Hulstaert leg.; KMMA • 1♂ 1♀; Bambesa; 21 Mar. 1938; J. Vrydagh leg.; KBIN • 1♂; same collection data as for preceding; 6–9 Nov. 1938 • 1♂; Bambesa; 10 Aug. 1933; J.V. Leroy leg.; KMMA • 1♀; same collection data as for preceding; 10 Oct. 1933 • 1♀; same collection data as for preceding; 20 Oct. 1933 • 1♂; same collection data as for preceding; Dec. 1934 • 3♂♂ 1♀; same collection data as for preceding; 15 Oct. 1933; H.J. Brédo leg. • 1♂; same collection data as for preceding; 1 Oct. 1938; J. Vrydagh leg. • 6♂♂ 5♀♀; same collection data as for preceding; Jul. 1943; J. Vrydagh leg. • 1♂; Bangala, Kutu; 18 Jun. 1935; G. Settembrino leg.; KBIN • 1♂; Barumbu; Feb. 1921; J. Ghesquière leg.; KMMA • 1♀; Barumbu; 22 Jun. 1921; Tihou; KMMA • 1♂; Bas Congo; KMMA • 1♀; Basoko; 1911; Bequaert leg.; KMMA • 1♀; Basoko; Oct. 1948; P.L.G. Benoit leg.; KMMA • 1♀; Beni Bendi, Sankuru; Jan. 1895; L. Claetens leg.; KBIN • 1♀; Benzali, Kinshasa; 3 Oct. 1968; P.M. Elsen leg.; KMMA • 1♀; Bokuma, Tshuapa; Jul. 1952; P. Lootens leg.; KMMA • 1♀; same collection data as for preceding; 6 Feb. 1954 • 1♂; Bolingo, Busira River; 23 Jun. 1936; J. Ghesquière leg.; KBIN • 1♀; same collection data as for preceding; 24 Jun. 1936 • 1♀; Bombutu, Salonga; Jun. 1936; J. Ghesquière leg.; KBIN • 1♀; south of Bukavu, Tanganyika; 28 Aug. 1931; NHMUK • 2♀♀; Bumba; Dec. 1939–Jan. 1940; H. De Saeger leg.; KMMA • 1♀; Coquilhatville [= Mbandaka]; 12 May 1927; E. Mestdagh leg.; KBIN • 1♂; Costermansville [= Bukavu]; Jan.–Jun. 1951; H. Bomans leg.; KMMA • 1♀; Dekese, Itunda; Aug. 1959; F.J. François leg.; KBIN • 1♂ 1♀; same collection data as for preceding; Nov. 1959 • 1♀; same collection data as for preceding; Dec. 1959 • 1♂; same collection data as for preceding; 10 Feb. 1960 • 1♂; same collection data as for preceding; 23 Feb. 1960 • 1♀; Dingila, Uelé; 15 Jul. 1933; J.V. Leroy leg.; KMMA • 1♂; Djambi; 23 Dec. 1913; CNC • 1♂; Eala; Apr. 1932; H.J. Brédo leg.; KMMA • 1♂; Eala; 27 Mar. 1935; J. Ghesquière leg.; KBIN • 1♂; same collection data as for preceding; 7 May 1935 • 1♂; same collection data as for preceding; 17 May 1935 • 1♀; same collection data as for preceding; 25 Jun. 1935 • 1♀; same collection data as for preceding; 12 Aug. 1935 • 1♂; same collection data as for preceding; Aug. 1935 • 1♀; same collection data as for preceding; 25 Sep. 1935 • 2♂♂ 1♀; same collection data as for preceding; Sep. 1935 • 1♀; same collection data as for preceding; 25 Oct. 1935 • 1♀; same collection data as for preceding; Oct. 1935 • 1♀; same collection data as for preceding; 20 Nov. 1935 • 1♀; same collection data as for preceding; Nov. 1935 • 1♂; same collection data as for preceding; Jan. 1936 • 1♀; same collection data as for preceding; 6 Feb. 1936 • 2♂♂ 4♀♀; same collection data as for preceding; Mar. 1936 • 1♀; same collection data as for preceding; 19 Aug. 1936 • 1♂; same collection data as for preceding; 28 Sep. 1936 • 1♂ 1♀; same collection data as for preceding; Sep. 1936 • 1♂; same collection data as for preceding; 7 Oct. 1936 • 1♂; same collection data as for preceding; 22 Oct. 1936 • 1♂; same collection data as for preceding; 23 Oct. 1936 • 1♂; same collection data as for preceding; 26 Oct. 1936 • 1♀; same collection data as for preceding; 27 Oct. 1936 • 1♀; same collection data as for preceding; 6 Nov. 1936 • 1♂; same collection data as for preceding; 10 Nov. 1936 • 1♂; same collection data as for preceding; 24 Nov. 1936 • 1♂; Elisabethville [= Lubumbashi]; 26 Feb. 1939; H.J. Brédo leg.; KMMA • 1♂; Etata, Tshuapa; Sep–Oct. 1969; J. Hauwaerts leg.; KMMA • 1♀; Flandria [= Boteka], Tshuapa; Sep. 1946–Aug. 1947; P. Hulstaert leg.; KMMA • 1♀; Gamangui, Ituri; Feb. 1910; Lang and Chapin leg.; KMMA • 1♂; Gandjo, P.N. Albert; 1934; G.F. de Witte; KMMA • 1♀; Goma, Kivu; 10–15 May 1953; J. Verbeke leg.; KBIN • 1♀; Gombari, Uelé; D.J. Rodhain leg.; KMMA • 1♀; Inongo; Aug. 1913; J. Maes leg.; KMMA • 1♀; Irangi, Kivu; Feb. 1986; E. Heiss leg.; CDFA • 1♀; Isangi; May 1949; R.P. Camps leg.; KMMA • 2♂♂ 1♀; Ituri Forest; Jul. 1976; E. Babyetagara leg.; CNC • 1♀; Jenge; Dec. 1931; Putnam leg.; KMMA • 1♀; Jumbi, Mayumbe; 5 May 1926; A. Collart leg.; KMMA • 2♀♀; Kabinda; 1914; J. Schwetz leg.; NHMUK • 3♂♂ 2♀♀; Kadjudju; May–Jun. 1932; G. Babault leg.; MNHN • 4♂♂ 2♀♀; same collection data as for preceding; Aug. 1932 • 1♂ 1♀; Kakanou, Lomami; Jun. 1918; J. Schwetz leg.; KBIN • 1♀; Kalenge, Lulua; Aug. 1932; G.F. Overlaet leg.; KMMA • 3♀♀; same collection data as for preceding; Feb. 1934 • 1♀; Kalonge, P.N. Albert; 23–31 Jul. 1952; P. Vanschuytbroeck and J.Kekenbosch leg.; KMMA • 1♀; same collection data as for preceding; 27–30 Jul. 1952 • 1♀; Kapanga, Lulua; Nov. 1928; Walker leg.; KMMA • 1♀; Kapanga, Lulua; Aug. 1932; G.F. Overlaet leg.; KMMA • 2♀♀; same collection data as for preceding; Nov. 1932 • 1♂; same collection data as for preceding; Jan. 1933 • 1♂ 4♀♀; same collection data as for preceding; Mar. 1933 • 1♂ 1♀; same collection data as for preceding; Nov. 1933 • 1♂; same collection data as for preceding; Nov. 1933; CNC • 1♂; Kelzongo, Ubangi; 1 Feb. 1932; H.J. Brédo leg.; KMMA • 1♀; Keshero, Kivu; 27 Aug. 1953; J. Verbeke leg.; KBIN • 1♀; Kibali, Yindi, Ituri; May 1949; A.E. Bertrand leg.; KMMA • 1♂; Kibombo; 31 Oct. 1910; Bequaert leg.; KMMA • 1♀; Kisantu; 1932; De Wulf leg.; KMMA • 1♀; Kikyo, nr Kalonge, P.N. Albert; 10 Aug. 1952; R.P. Lootens leg.; KMMA • 1♀; Kissenyi, Kivu; 25 Apr. 1923; Van Saceghem leg.; KMMA • 1♀; same collection data as for preceding; 1924 • 1♀; Komi, Sankuru; Dec. 1930; J. Ghesquière leg.; CNC • 1♂; same collection data as for preceding; 8 Apr. 1930; KMMA • 3♀♀; same collection data as for preceding; May 1930 • 2♂♂; same collection data as for preceding; Jun. 1930 • 1♀; same collection data as for preceding; Dec. 1930 • 1♀; Kona; 16 May 2010; P. Grootaert leg.; KMMA • 1♂ 1♀; Kondue, Sankuru, E. Luja leg.; KMMA • 1♂; Lemba, Mayumbe; 1–10 Dec. 1915; R.Mayné leg.; KMMA • 1♀; Leverville [= Lusanga]; 1♂; Jan. 1914; P. Vanderijst leg.; KMMA • 1♀; 1928; J. Tinant leg.; KMMA • 1♂; Lokandu, Biawa Island; Jul. 1939; Vissers leg.; KMMA • 1♂ 1♀; Lubutu; 22 Jan. 1915; J. Bequaert leg.; KMMA • 1♂; Lueba near Baraka, Lake Taganyika; 16 Apr. 1927; R. Bois leg.; NHMUK • 1♂; Luebo; 30 Aug. 1921; H. Schouteden leg.; KMMA • 1♂; Lukolela; Nov. 1934; Ledoux leg.; KMMA • 1♀; Luputa, Lomami; Sep. 1934; Bouvier leg.; KMMA • 3♀♀; same collection data as for preceding; May 1935 • 1♂; Lusambo; 1938; V. Lagae leg.; KMMA • 1♂; Luvungi; Feb. 1916; Rodhain leg.; KMMA • 1♀; Mayumbe; 1917; R. Mayné leg.; KMMA • 1♀; Mbau, North Kivu; 8–21 Dec. 1971; H. Falke leg.; CNC • 1♀; Moanda; KMMA • 1♀; Mobeka, Ubangi; 11 Aug. 1947; M. Poll, leg.; KMMA • 2♀♀; Moma, Equateur; Jun. 1925; J. Ghesquière and Prince Leopold leg.; KMMA • 2♂♂; Mongbwalu, Kilo; 1937; Scheitz leg.; KMMA • 1♀; Mulungu, Kivu; 5 Apr. 1937; H.J. Brédo leg.; KMMA • 1♀; Mulungu, Kivu; Nov. 1951; P. Lefèvre leg.; KMMA • 1♂; Niangara, Uelé; Nov. 1910; Lang and Chapin leg.; KMMA • 1♂; Ouellé, Bouta; A. Dubois leg.; MNHN • 1♀; Poko; Uelé; Aug. 1913; Lang and Chapin leg.; CNC • 13♂♂ 18♀♀; same collection data as for preceding; KMMA • 1♂; Rumangabo, Nyakibanda, P.N. Albert; 11–13 Apr. 1945; G.F. de Witte leg.; KMMA • 1♀; Rutshuru; Jan. 1928; Ch. Seydel leg.; KMMA • 2♀♀; Rutshuru; 24 Nov. 1952; J. Verbeke leg.; KBIN • 1♀; Sake, nr Mutayo River, Kivu; 21 Mar. 1953; J. Verbeke leg.; KBIN • 1♂; Sankuru; 1910; Abrassart leg.; KMMA • 1♂; Shakakombe; 20 Apr. 1959; F.J. François leg.; KBIN • 1♀; Stanleyville [= Kisangani]; Lang and Chapin leg.; CNC • 1♂; same collection data as for preceding; Mar. 1915 • 2♂♂ 3♀♀; same collection data as for preceding; NHMUK • 1♂ 2♀♀; same collection data as for preceding; KMMA • 1♂; Tshibinda, Kivu; Dec. 1927; Ch. Seydel leg.; KMMA • 1♀; Uelé River; Rodhain leg.; KMMA • 2♂♂; Uruwu to Beni; Dec. 1935; F. François leg.; KMMA • 1♀; Uvira; Aug.–Dec. 1949; G. Marlier leg.; KMMA • 1♀; Wamba; 1936; Degotte leg.; KMMA • 1♀; Yahuma; Dec. 1948; P.L.G. Benoit leg.; KMMA • 1♀; Yangangu, Libenge; Oct.–Nov. 1945; C. Henrard leg.; KMMA. Equatorial Guinea • 1♀; Nkolentangan; 17 Nov. 1907; S.G. Tessmann leg.; MNB • 2♂♂ 3♀♀; San Benito [= Mbini]; 1885; Guiral leg.; MNHN. Gabon • 1♀; Lambaréné; 1921; E. Le Moult leg.; MNHN • 1♀; Lastourville; G. Le Testu leg.; MNHN • 2♀♀; Libreville; Aug. 1892; MNB • 1♂; Libreville road, 50 km from Makokou; 8 May 1974; M. Donskoff and J. Le Breton leg.; MNHN • 1♀; Mabilé range; 14 Apr. 2016; G. Goergen leg.; IITA • 1♂; Makokou; Jul. 1970; J. David leg.; MNHN • 1♂; Muni, Mts de Cristal; 15–31 Oct. 1969; A. Villiers leg.; MNHN. Ghana • 1♀; Aburi; 1912–1913; W.H. Patterson leg.; NHMUK • 1♀; Ayona; May 2008; G. Goergen leg.; IITA • 1♀; Buro-Buro, Kumasi; 24 May 1976; A.B. Stam leg.; KMMA • 1♀; Cape Coast; Dec. 2004; G. Goergen leg.; IITA • 1♀; Kade; 3 Apr. 1964; C. Dudley leg.; NMSA • 1♀; Nadia; 1913; A.E. Evans leg.; NHMUK • 1♂; Nyabo, Ashanti; 29 Mar. 1947; J. Bowden leg.; NHMUK • 1♀; Obuasi; 20 Feb. 1906; W.M. Graham leg.; NHMUK • 1♀; same collection data as for preceding; 11 Aug. 1906 • 1♀; same collection data as for preceding; 24 Jul. 1907 • 1♀; same collection data as for preceding; 4 Aug. 1907 • 1♀; same collection data as for preceding; [no date] • 12♂♂ 7♀♀; Wati Waterfalls; Feb. 2003; G. Goergen leg.; IITA. Guinea Conakry • 3♀♀; Tamara Island, Iles de Los; Jul. 1913; J. Serand leg.; MNHN • 1♀; Yanlé; Jun. 1942; M. Lamotte leg.; MNHN. Ivory Coast • 1♀; Daloa; Dec. 1930–Apr. 1931; Ch. Alluaud and P.A. Chappuis leg.; MNHN • 1♀; Danané; Jan. 1939; L. Chopard leg.; MNHN • 1♀; Duékué; Dec. 1930–Apr. 1931; Ch. Alluaud and P.A. Chappuis leg.; MNHN • 1♀; Koun-Abronso; Sep. 1961; J. Decelle leg.; KMMA • 1♀; Odienne; 1 May 1973; V. Vittard leg.; MNHN. Kenya • 1♀; Bura, Taita; Mar. 1912; Alluaud and Jeannel leg.; MNHN • 1♀; Bwamba; Jul.–Aug. 1946; V.G.L. van Someren leg.; NHMUK • 2♀♀; Ilala, 14 mi E Mumias; 18–21 Jun. 1911; S.A. Neave leg.; NHMUK • 1♀; Kaimosi; Mar.–Apr. 1932; V.G.L. van Someren leg.; NHMUK • 1♀; same collection data as for preceding; Feb. 1949 • 1♂; Kakamega Forest; 18–22 Jan. 1972; C.F. Huggins leg.; NHMUK • 1♂; Mawakota; Jul. 1929; V.G.L. van Someren leg.; NHMUK • 1♀; Nyangnori, West Nandi; Oct. 1904; Alluaud leg.; MNHN • 1♀; Taveta; Aug. 1947; V.G.L. van Someren leg.; NHMUK • 1♂; Yala River, S edge Kakamega Forest; 21–28 May 1911; S.A. Neave leg.; NHMUK. Liberia • 1♀; House Voinjana; 3 Sep. 1959; NHMUK • 1♀; river camp 3; 1926; J. Bequaert leg.; KMMA. Malawi • 1♂; Chintheche; Dec. 1977; R. Jocqué leg.; KMMA • 1♀; Mt Mlanje; 26 Oct. 1912; S.A. Neave leg.; NHMUK • 1♂; same collection data as for preceding; 6 Apr. 1913 • 1♀; Mt Mlanje; Jan.–Feb. 1914; J.B. Davey leg.; NHMUK • 1♂; 10 km W Nkhata Bay; 5–6 Mar. 1987; J. and A. Londt leg.; NMSA. Mozambique • 1♀; Tumbine Mountains, Milange; Apr. 1958; B. Stuckenberg leg.; NMSA. Nigeria • 1♂ 1♀; Aiyangba, N Nigeria; 11 Jan. 1911; T.J. Simpson leg.; NHMUK • 1♂; Ibadan, IITA Centre; Oct. 1997; G. Goergen leg.; IITA • 2♂♂; same collection data as for preceding; Nov. 2001 • 2♀♀; Ile-Ife; 30–31 Dec. 1969; J.T. Medler leg.; CNC • 1♂; S. Nigeria; T.J. Simpson leg.; NHMUK. Republic Of The Congo • 2♀♀; Lambaréné, Ogooué; E. Ellenberger leg.; MNHN • 1♀; same collection data as for preceding; 1911 • 2♀♀; same collection data as for preceding; 1912 • 3♀♀; same collection data as for preceding; 1913 • 2♀♀; N’Kogo, Ogooué; 1901; J. Bouyssou leg.; MNHN • 2♂♂; N’Goko-Sanga Basin, Ouesso; 1906; J. Gravot leg.; MNHN • 1♀; Ubokasanga; 1906; D. Gnavorod leg.; MNHN. Rwanda • 1♀; Ninda, P.N. Albert; 26 Sep. 1934; G.F. de Witte leg.; KBIN • 1♀; Rubona; 17 Jun. 1955; P. Elsen leg.; KMMA • 1♀; Shangugu; 23 Oct.–15 Nov. 1948; F.J. François leg.; KBIN • 1♀; Shangugu; 6 Apr. 1953; P. Basilewsky leg.; KMMA. Tanzania • 1♀; Bukoba; 10 Jun. 1912; C.C Gowdey leg.; NHMUK • 1♀; Amani; G. Pringle leg.; NHMUK • 1♂; Amani; 10 Dec. 1960; CNC • 1♂ 1♀; Morogoro; 6 May 1925; A.H. Ritchie leg.; NHMUK • 1♂ 2♀♀; same collection data as for preceding; 10 May 1925 • 2♂♂; same collection data as for preceding; 16 May 1925. Togo • 2♀♀; Dzogbégan; 9 Jun. 1999; G. Goergen leg.; IITA • 1♂; Kloto; Aug. 2003; G. Goergen leg.; IITA • 1♀; same collection data as for preceding; Dec. 2003 • 30♂♂ 20♀♀; same collection data as for preceding; Jan. 2004 • 35♂♂ 24♀♀; same collection data as for preceding; Feb. 2004 • 7♂♂ 4♀♀; same collection data as for preceding; Mar. 2004 • 3♂♂ 2♀♀; same collection data as for preceding; Apr. 2004 • 1♂; same collection data as for preceding; Jan. 2005 • 12♀; same collection data as for preceding; Oct. 2005 • 1♂ 1♀; same collection data as for preceding; Nov. 2005 • 1♂; same collection data as for preceding; Feb. 2006 • 4♂♂; same collection data as for preceding; Feb. 2007 • 1♂; same collection data as for preceding; Jan. 2008 • 1♂; same collection data as for preceding; Nov. 2015 • 1♀; same collection data as for preceding; 21–24 Jan. 2016 • 1♀; Kloto Forest; Oct. 2005; G. Goergen leg.; IITA • 2♂♂ 1♀; Kuma-Tokpli; 21–24 Jan. 2016; G. Goergen leg.; KMMA. Uganda • 1♀; Ankole, West Uganda; 30 Dec. 1975; M.K. Paulus leg.; CNC • 1♀; Ankole-Toro border, E Lake George; 20–21 Oct. 1911; S.A. Neave leg.; NHMUK • 2♀♀; Buamba Forest, Semliki Valley; 3–7 Nov. 1911; S.A. Neave leg.; NHMUK • 1♂; Budongo Forest, Unyoro; 11–15 Dec. 1911; S.A. Neave leg.; NHMUK • 1♂; Buramba Forest, Toro District; 22 Oct. 1913; R.E. McConnell leg.; NHMUK • 1♂; East Busoga, between Jinja and Busia or Mbwago; 28 Jul–1 Aug. 1911; S.A. Neave leg.; NHMUK • 1♀; Bwamba; 1 Jul. 1976; E. Babyetagara leg.; CNC • 1♂; Bwamba Valley; Jul. 1945; V.G.L. van Someren leg.; CNC • 1♀; Doro or Durro Forest, Toro; 25–29 Oct. 1911; S.A. Neave leg.; NHMUK • 1♂ 1♀; Mount Elgon; 4–8 Dec. 1972; H. Falke leg.; CNC • 1♀; Entebbe; 1–11 Sep. 1911; S.A. Neave leg. • 1♂ 3♀♀; same collection data as for preceding; 18–20 Nov. 1912; C.C. Gowdey leg. • 1♂; same collection data as for preceding; 3–4 Dec. 1912 • 2♂♂; same collection data as for preceding; 12–13 Dec. 1912 • 1♂; same collection data as for preceding; 1959; P.S. Corbet leg. • 1♀; Entebbe; 16 Oct. 1971; H. Falke leg.; CNC • 1♀; same collection data as for preceding; 17 Jun. 1972 • 1♂; same collection data as for preceding; 26 Oct. 1971; ZFMK • 9♀♀; near Entebbe; 1–14 Feb. 1973; H. Falke leg.; CNC • 2♀♀; Gayaza, Ankole; 17 Mar. 1936; H.B. Johnston leg.; NHMUK • 1♂ 3♀♀; Lake George; 17–19 Oct. 1911; S.A. Neave leg.; NHMUK • 1♀; Hoima-Kampala Road; 3 Jan. 1912; S.A. Neave leg.; NHMUK • 1♂ 1♀; Ibanda; 23–28 Dec. 1972; H. Falke leg.; CNC • 2♂♂; same collection data as for preceding; 19–24 Apr. 1973 • 1♂ 1♀; Impenetrable Forest, Ruhiza, Kigezi; 1–10 Jun. 1972; E. Babyetagara leg.; CNC • 1♂; Jinja; Sep. 1928; V.G.L. van Someren leg.; NHMUK • 1♂; same collection data as for preceding; Oct. 1930 • 1♂; Katera Forest, South of Maseka; May 1972; E. Babyetagara leg.; CNC • 2♂♂ 2♀♀; Kayonza Forest, Kigezi; May 1972; E. Babyetagara leg.; CNC • 2♂♂; Mabira Forest, Chagwe; 16–25 Jul. 1911; S.A. Neave leg.; NHMUK • 1♂; Maramagambo Forest, Lutoto, on Lake Edward Road; 1 Feb. 1912; H.B. Edwards leg.; NHMUK • 1♀; Masinda; Feb. 1912; R. Fyffe leg.; NHMUK • 1♀; Mbali; 19 Aug. 1912; C.H. Marshall leg.; NHMUK • 1♀; Mbarara, Ankole; Sep. 1966; M.G. Jefferies leg.; NHMUK • 1♂; Mbuma; 22 May 1910; C.A. Wiggins leg.; NHMUK • 1♀; Mpanga Forest, Toro; 13–23 Nov. 1911; S.A. Neave leg.; NHMUK • 1♂; Nakasongola; 28 Jul. 1911; C.C. Gowdey leg.; NHMUK • 1♂; same collection data as for preceding; 28 Aug. 1912 • 2♀♀; Nkokonjera; 23 Dec. 1910; C.C. Gowdey leg.; NHMUK • 1♀; Nyanza Victoria Northwest shores; 12–15 Sep. 1911; S.A. Neave leg.; NHMUK • 1♂; Nyrbthozi County, Nyabushozi; 21 Jan. 1975; P. Mugabi leg.; CNC • 1♂; Ruwenzori Foothills; 3–8 Jan. 1972; H. Falke leg.; CNC • 1♂; Rumi River; [no date]; J. Fraser leg.; NHMUK • 1♀; same collection data as for preceding; 12 Feb. 1912 • 1♀; Semliki Forest; 18 Feb. 1912; J. Fraser leg. NHMUK. Zimbabwe • 1♀; Bomponi; 16 May 1965; D. Cookson leg.; NMSA • 1♂; same collection data as for preceding; 24 May 1965 • 1♂; N. Vumba; 7 Apr. 1966; D. Cookson leg.; NMSA • 1♀; Zonwi River bridge; 17 Feb. 1963; A. van Bruggen leg.; NMSA.

**Figures 24–27.**
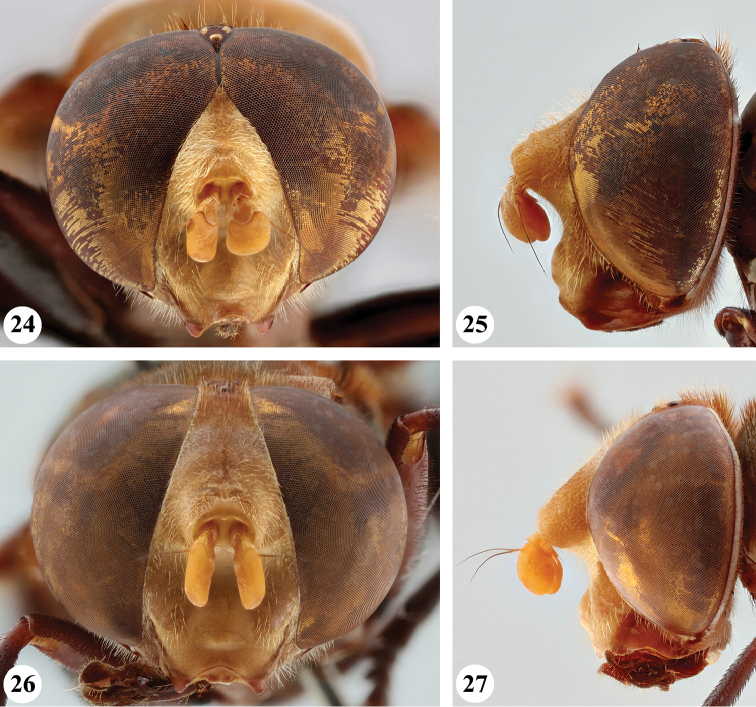
Head, frontal and lateral view *S. flaviceps* Macquart (**24, 25** ♂ **26, 27** ♀).

#### Description.

Body length: 13.5–18.2 mm. Wing length: 11.0–12.7 mm.

**Male** (Fig. 2). Head (Figs 16, 17). Eye bare, holoptic; eye contiguity for distance at most 1.5 times length of ocellar triangle, facets slightly enlarged in dorsal half, ca. three times as large in diameter as ventral facets. Frons dark rufous to black-brown; largely subshiny with black pollinosity in dorsal fifth only; dispersed short dark pile, dorsally somewhat longer. Face ground colour rufous (very rarely more black-brown); mainly shining with weak grey pollinosity only below antenna, along eye margin and below lateral facial tubercle; in parts with dispersed short pale pile; facial tubercle strongly pronounced, distinct lateral tubercle present. Gena colour and pollinosity as face; with short to long pale pile. Occiput black-brown, along eye margin more orange, with dispersed pale pile. Antennal segments and arista rufous.

***Thorax*** (Fig. 53). Scutum subshiny black; with very short black pile; postpronotum and notopleuron more orange to rufous and with longer pale or dark pile; transverse suture with fascia of black pollinosity. Scutellum clearly marginated, distinctly rounded and at most twice as wide as long; rufous, sometimes darker; with short dark pile, along margins paler pile. Pleura ground colour black to black-brown, along anterior spiracle more orange to rufous, covered with dispersed long dark pile except on meron, dorsomedial anepimeron, ventral part of katepimeron, anterior part of katepisternum and anterior anepisternum.

***Legs***. Orange to rufous (very rarely more brown), metatarsomeres 3–5 darker; with short pale orange pile, along posterior margin of pro- and mesofemora and ventral margin of metafemur with longer pile, sometimes pile partially dark red. Metaleg (Fig. 81), femur slender, with slight ventral swelling in apical fifth, pile at swelling black and more dense; tibia slightly thickened and curved, pile along ventral margin in apical half to two-thirds longer and more dense.

***Wing*** (Fig. 65). Brownish, except alula and along posterior margin where hyaline; hyaline area not distinctly demarcated. Calypters dull chalk-white to yellow with fringe of silvery white to yellow pile (Figs 66, 67). Cell r_1_ closed, petiole usually shorter than height of base of stigma, at most as long as base. Vein R_4+5_ sinuate and short appendiculate, sometimes appendix missing.

***Abdomen*** (Fig. 92). Uniformly subshiny black to black-brown, rarely posteriorly more rufous; with short dark pile except tergum I where silver-grey. Sterna rufous to black-brown; with dispersed long pale pile except for sternum IV and postabdomen where dark. Male genitalia as in Fig. 103.

**Female.** As male except for the following character states: Eye dichoptic (Figs 18, 19), dorsal facets slightly larger than ventral ones. Frons with rufous to black pollinosity in dorsal part for length equal to ocellar triangle; with short dark pile except dorsal of antennal implant where intermixed with greyish pile. Scutellum usually darker, almost concolourous with medial part of scutum, sometimes more than twice as wide as long.

#### Distribution.

Angola, Benin, Burundi, Cameroon, Central African Republic, Democratic Republic of the Congo, Equatorial Guinea, Gabon, Ghana, Guinea Conakry, Ivory Coast, Kenya, Liberia, Malawi, Mozambique, Nigeria, Republic of the Congo, Rwanda, Sierra Leone, Tanzania, Togo, Uganda, Zimbabwe. Record from Colombia for the type specimen of *nigripennis* is probably an error as the species is not reported from the Neotropical Region (see Comments).

**Figures 28–31.**
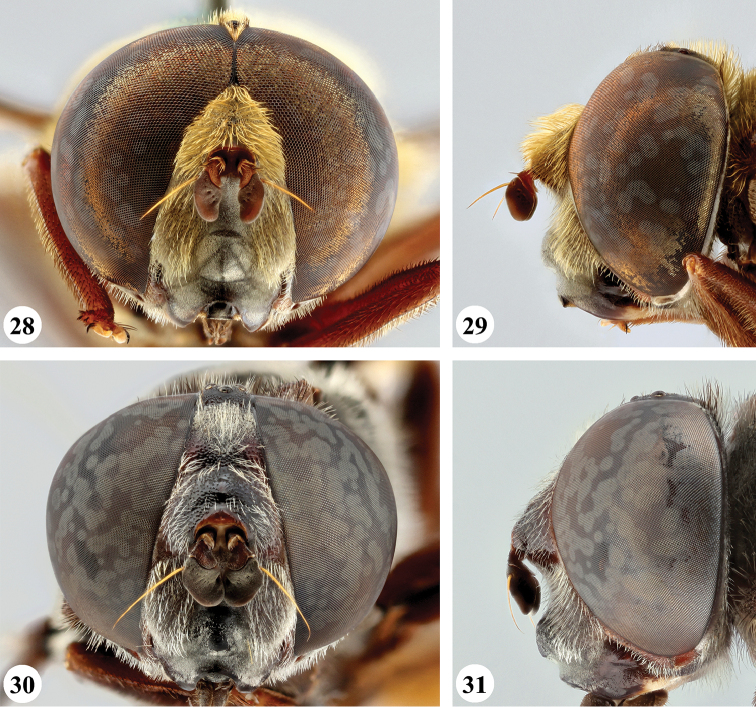
Head, frontal and lateral view *S. haemorrhoa* (Gerstaecker) (**28, 29** ♂ **30, 31** ♀).

#### Comments.

This species has been described under several names but most of them were synonymized subsequently. No distinct character states could be discerned to confirm the specific status of these taxa, so the different synonymies are confirmed. The description of *S. nigripennis* deviates in some aspects from the morphological character states observed in *S. dibapha*: “face d’un noir brunâtre luisant” [face shiny brownish black]; “Thorax et abdomen… à légers reflets verts” [Thorax and abdomen with light greenish reflections]; “jambes postérieures…. ciliées de noir” [posterior legs… black ciliated]. Other character states correspond with *S. dibapha*. However, the two syntypes of *S. nigripennis* in the NHMUK collection do not correspond with the above mentioned deviations, although one syntype (partially covered in mould) does have a darker facial colour than usually observed and the pilosity of the metatibia is dark reddish (as observed in other specimens). Speiser (1913) also pointed out a slightly different shape of the scutellum in *S. nigripennis*, compared to *S. dibapha*, but this seems to fall within the variability observed in the material examined. In all other aspects they are identical to other material of *S. dibapha* studied, including the type. They are labelled as originating from Colombia, as stated in the original description by Macquart: “De la Colombie”. Speiser (1913) already suggested the synonymy, but he did not formally designate it based on the geographical issue. Thompson et al. (1976) in the Neotropical catalogue listed this under the genus *Palpada* Macquart, 1834 as a new combination and indicated Oxford museum as type depository. However, no type specimen could be traced in the OXUM collection (Z. Simmons, pers. comm.). Montoya (2016) did not list *nigripennis* anymore in his catalogue of Syrphidae from Colombia. The Systema Dipterorum website (Evenhuis and Pape 2020) listed it as junior synonym of *S. dibapha*. We consider the type material at NHM as being representative and hence *S. nigripennis* as being synonymous with *S. dibapha*. The type of *S. dibapha* bears a lectotype label by F.C. Thompson, dated 1987, but this lectotype designation does not seem to have been published. Here we thus formally place *nigripennis* Macquart as synonym of *dibapha* Walker.

Speiser (1910) described *gypseisquama* from a number of specimens originating from West (Cameroon and Sierra Leone) and East (Tanzania, Uganda) Africa. Afterwards, Speiser (1911) considered the material from West Africa as a separate variety (*sulfurata*), based on the coloration of the calypters. Of the specimens from West Africa, a number could be traced in the MNB collection (see above type material examined). The material from Uganda (for *gypseisquama*) and part of the material from Barombi, Cameroon and the male specimen from Kondué, Democratic Republic of the Congo (for var. sulfurata) could, however, not be traced. The female specimen from Sierra Leone in the MNB collection (apparently received or purchased from Staudinger) could be the type referred to as ‘ein angekauftes F aus Sierra Leone’ in Speiser (1910). Although the latter is not explicitly mentioned in Speiser (1911) as type of var. sulfurata, it is implied under “Alle diese Exemplare haben hell citronengelbe statt weisser Schüppchen, sind aber sonst völlig gleich mit den Ostafrikanern” [All of these specimens have bright lemon yellow instead of white calypters, but are otherwise identical to the East Africans] (in Speiser 1910) and “westafrikanische Form als besondere Varietät mit einem Namen zu belegen” [to assign a name to the West African form as a special variety] (in Speiser 1911).

Smith and Vockeroth (1980) listed *sulfurata* as a distinct species but as junior synonym of *dibapha*. As indicated the colour of the lower calypter is variable from yellowish to bright white. Speiser (1911) suggested that there is a geographical separation between *S. gypseisquamasensu stricto* found in eastern Africa, and the variety *sulfurata* found in West and Central Africa. However, we have studied long series throughout the full geographical range and do not confirm this pattern. Both colour forms were found in close by or same locations (Figs 66, 67, material from same locality in the Democratic Republic of the Congo: Dekese, Itunda). DNA barcodes of specimens from West (i.e., Benin, Ghana, Togo), Central (D.R. Congo) and East (Uganda) Africa do not show any separation (Suppl. material 1: Fig. S1). No other correlated characters states could be linked to the color of the lower calypter. Therefore, the character is considered to be variable, not related to geographic distribution and of no taxonomic importance.

**Figures 32–35.**
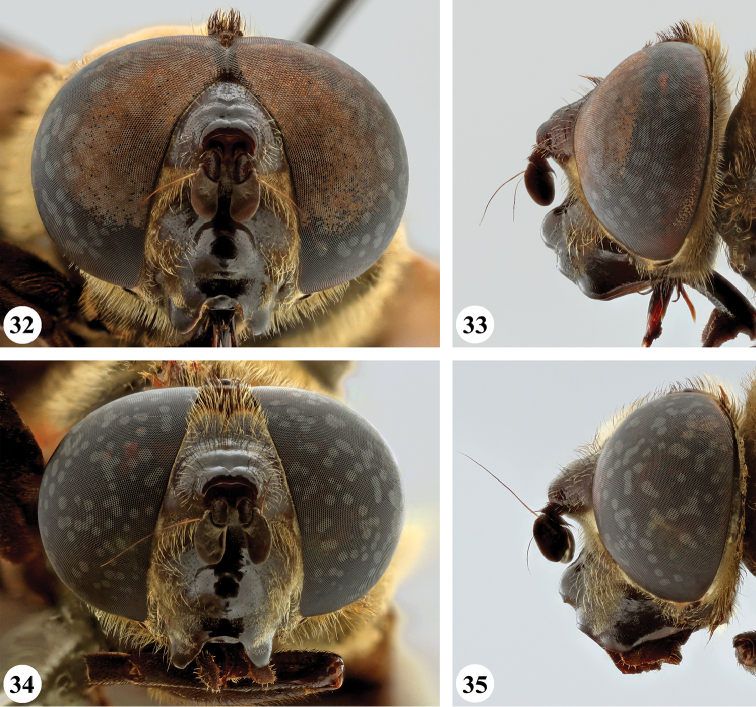
Head, frontal and lateral view *S. melanthysana* (Speiser) (**32, 33** ♂ **34, 35** ♀).

### Senaspis elliotii

Taxon classificationAnimaliaDipteraSyrphidae

Austen, 1909

FD90378F-6CF7-55C2-91F1-F39E3D1A385F

Figs 3 , 20–23 , 54 , 55 , 68–71 , 82 , 93 , 104

Senaspis elliotii Austen, 1909: 90.
Eristalis (Stenaspis) ellioti var. claricella Speiser, 1910: 123.

#### Differential diagnosis.

Species differentiated from all other *Senaspis* species by the conspicuous dense white to yellow pile on scutum, contrasting with the black pile on pleura (Fig. 3) (other species with dense pale pilosity on the scutum like *S. melanthysana* and *S. umbrifera*, have the pale pile continued on pleura (Figs 6, 9)). The wing is predominantly dark, except along posterior margin and at the apex where hyaline (Fig. 68).

#### Examined material.

*Senaspis elliotii* Austen: Lectotype (hereby designated), male, “Syn-//type” “Senaspis // Type // elliotti // Austen, ♂” “Ruwenzori, // Scott Elliot.5. // 7–8,000 ft. // 95–41.” “LECTOTYPUS” [NHMUK]. Paralectotype, female, “Syn-//type” “Makumbu, // B.E. Africa. // C.S. Betton. // 1900.35 // 6.II–8.III.99.” “PARA- // LECTOTYPUS” [NMHUK]. Paralectotype, female, “Syn-//type” “Senaspis // Type // elliotti // Austen, ♀” “Between Salt Lake//& Wawamba Country // Ruwenzori district, // Uganda // 1894. // G.F. Scott Elliot // 95.41” “PARA- // LECTOTYPUS” [NHMUK].

**Figures 36–39.**
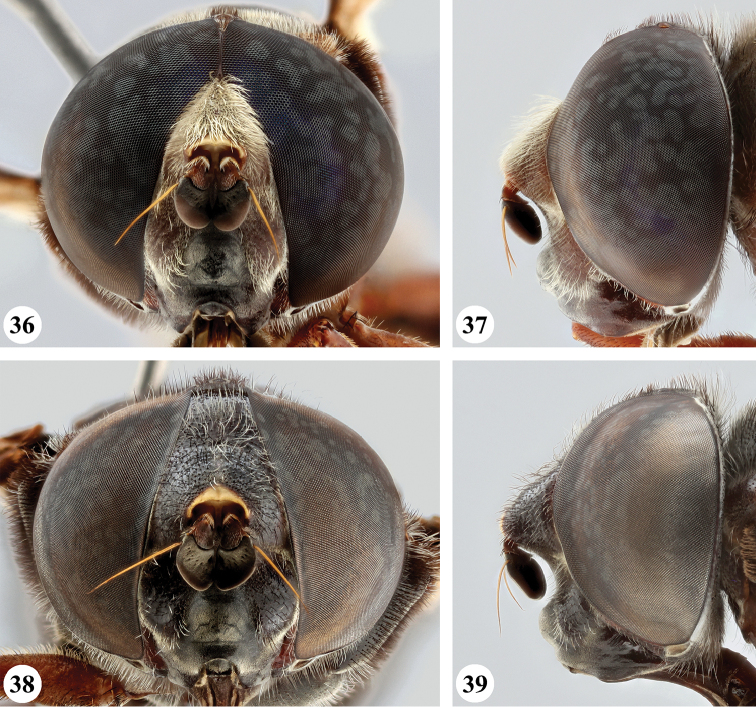
Head, frontal and lateral view *S. nigrita* (Bigot) (**36, 37** ♂ **38, 39** ♀).

Eristalis (Stenaspis) ellioti var. claricella Speiser: Holotype, male, “Kilimandj. // Sjöstedt” “13 dec.” “Obstgarten- // Steppe” “Eristalis (Stenaspis) // ellioti Aust. var. claricella // P. Speiser det. // Typ. der Var.” “NHRS-BYWS // 000002772” “Loan // 575/99” [NRMS, examined through images].

#### Other material.

Angola • 1♀; Nova Gaia; 14–21 Dec. 1957; G.H. Heinrich leg.; CNC • 1♀; 30 km N Quiculungo; Sep.–Oct. 1957; CNC. Burundi • 1♂; Bururi; Jun. 1948; F.J. François leg.; KBIN • 1♀; Bururi; May 1960; R.P. Giraudin leg.; MNHN • 1♀; Bututsi, Mugamba; Jul.–Aug. 1948; F.J. François leg.; KBIN • 1♂; Cankuzo, Ruvubu Park; Jun. 2010; F. Ngendakuriyo leg.; OBPE • 1♀; Kibira Tinyabakwe; 4 Nov. 2010; OBPE • 2♂♂; Makamba; 28–29 May 1948; F.J. François leg.; KBIN • 1♂; same collection data as for preceding; 19 May 1949 • 1♀; Mt Bururi; May 1948; F.J. François leg.; KBIN • 1♂; Muhinga, Michinga Hill, Butezana; 11 May 1952; F.J. François leg.; KBIN • 1♂; Mukungu; 26 [?] 2013; L. Ndayikeza leg.; OBPE • 1♀; Murmera-Umushiha; 2011; OBPE • 1♀; Nyakibande, Mumirwa; 27 Jan. 2018; E. Sinzinkayo leg.; OBPE • 1♀; same collection data as for preceding; 24 Apr. 2019. Democratic Republic Of The Congo • 1♀; Bitale, Kalehe, Kivu; 23 May 1950; G. Marlier leg.; KMMA • 1♀; Boswenda; 22 Oct. 1914; J. Bequaert leg.; KMMA • 1♀; Bulira; Nov. 1932; L. Burgeon leg.; CNC • 2♀♀; same collection data as for preceding; KMMA • 1♀; Elisabethville [= Lubumbashi]; 25 Apr. 1927; M. Bequaert leg.; KMMA • 1♂; same collection data as for preceding; [no date]; KBIN • 1♀; same collection data as for preceding; 7 Sep. 1927 • 1♀; same collection data as for preceding; 10 Feb. 1928 • 1♀; same collection data as for preceding; 30 Sep. 1929 • 1♀; same collection data as for preceding; Mar. 1930; L. Courtois leg.; KMMA • 1♀; 16 Sep. 1932; same collection data as for preceding; De Loose leg. • 1♂ 2♀♀; from Beni to Lesse; Jul. 1911; Murtula leg.; KMMA • 1♀; Ibanda, Ruwenzori Mts; 199; Ch. Alluaud leg.; MNHN • 1♀; Kabare, Shabunda; R. Mayné leg.; KMMA • 5♂♂ 5♀♀; Kadjudju, Kivu; May–Jun. 1932; G. Babault leg.; MNHN • 7♂♂ 11♀♀; same collection data as for preceding; Aug. 1932 • 1♂ 1♀; same collection data as for preceding; Sep. 1932 • 1♂; Kalembelembe, Baraka; Jul. 1918; R. Mayné leg.; KMMA • 2♀♀; Kalimabenge River, Uvira; 23 Mar. 1949; G. Marlier leg.; KMMA • 1♀; Kansenia; Nov.–Dec. 1929; R.P. de Montpellier leg.; KMMA • 1♀; Kapanga; Oct. 1932; F.G. Overlaet leg.; KMMA • 1♂; same collection data as for preceding; Nov. 1933 • 1♂; Kitembo; 1927; G. Babault leg.; MNHN • 1♀; Lubudi; 1947; R. Claire leg.; KMMA • 1♀; Lubumbashi; 23 Jul. 1970; A.B. Stam leg.; KMMA • 1♂ 1♀; Lulenga, Kivu; Dec. 1927; Ch. Seydel leg.; KMMA • 1♀; Mulungu; 19–26 Nov. 1932; L. Burgeon leg.; KMMA • 1♀; Rutshuru, Parc National Albert, Kivu; 15–25 Sep. 1933; G.F. de Witte leg.; KMMA • 1♀; Ruwenzori Mt.; 26 Apr. 1914; AMNH • 1♀; Stanleyville [= Kisangani]; Mar. 1915,; KMMA; Lang and Chapin leg.; KMMA • 1♀; Tshamugussa, Parc National Albert; 8–15 Aug. 1934; G.F. de Witte leg.; KBIN • 1♀; Tshaya; 1932; G. Babault leg.; MNHN • 1♂ 1♀; Tshibinda; Kivu; Nov. 1927; Ch. Seydel leg.; KMMA • 2♀♀; same collection data as for preceding; Dec. 1927. Ethiopia • 2♀♀; Maraquo; 3 May 1914; O. Kovacs leg.; NHMUK • 1♀; Mulata Mts, Harar; 22–25 Oct. 1920; AMNH. Kenya • 1♀; Gatamayu; 5 Feb. 1942; V.G.L. van Someren leg.; NHMUK • 1♀; Ilala, 14 mi E Mumias; 18–21 Jun. 1911; S.A. Neave leg.; NHMUK • 2♀♀; Jomboni Hills; May 1947; V.G.L. van Someren leg.; NHMUK • 1♂ 2♀♀; Kaimosi; Apr. 1922; NHMUK • 1♀; Kijabe; Feb. 1932; V.G.L. van Someren leg.; NHMUK • 1♂ 1♀; Mawakota; Apr. 1925; V.G.L. van Someren leg.; NHMUK • 1♀; same collection data as for preceding; Jul. 1929 • 1♂; Mombasa; Jun. 1926; V.G.L. van Someren leg.; NHMUK • 1♂; same collection data as for preceding; Apr. 1928 • 1♀; Nairobi; Aug. 1904; Ch. Alluaud leg.; MNHN • 2♂♂; Nairobi; Apr. 1927; V.G.L. van Someren leg.; NHMUK • 1♀; same collection data as for preceding; Nov. 1928 • 1♀; same collection data as for preceding; [no date]; F.J. Jackson leg. • 1♂; Ngong; 26 Oct. 1919; A. Loveridge leg.; NHMUK • 1♀; Ngong; Apr. 1934; V.G.L. van Someren leg.; NHMUK • 1♀; Ngong; Feb. 1941; V.G.L. van Someren leg.; NHMUK • 1♂ 1♀; Nyeri; Dec. 1948; V.G.L. van Someren leg.; NHMUK • 1♂; Taita Hills; Aug. 1947; V.G.L. van Someren leg.; NHMUK • 1♀; Tumutumu; Jan. 1939; V.G.L. van Someren leg.; NHMUK • 1♂; same collection data as for preceding; Apr. 1939 • 2♂♂; Yala River, S edge Kakumega Forest; 21–28 May 1911; S.A. Neave leg.; NHMUK. Malawi • 1♀; Blantyre; 1914; J.B. Davey leg.; NHMUK • 2♂♂ 1♀; Cholo; R.C. Wood leg.; NHMUK • 1♀; Fort Anderson [= Mlanje]; 24 Jul. 1908; R. Newstead leg.; NHMUK • 1♀; Fort Johnston [= Mangochi]; Jan. 1910; H.N. Tate leg.; NHMUK • 1♂; Limbe; 25 Sep. 1916; R.C. Wood leg.; NHMUK • 1♀; Maiwale; 12 m E. of Fort Johnston [= Mangochi]; 20 Sep. 1926; W.A. Lamborn leg.; OXUM [image examined only] • 1♂; Mlanje; 7 Dec. 1912; S.A. Neave leg.; NHMUK • 1♀; same collection data as for preceding; 20 Jan. 1913 • 1♀; same collection data as for preceding; 12 Feb. 1913 • 2♀♀; same collection data as for preceding; 7 May 1913 • 1♀; Zomba; H.S. Stannus leg.; CNC • 4♀♀; Zomba; H.S. Stannus leg.; NHMUK . Mozambique • 1♀; Revoué Valley, env. Andrada; Mar. 1905; G. Vasse leg.; MNHN. Rwanda • 1♂; Kibungu; Oct.–Dec. 1937; R. Verhulst leg.; KMMA • 1♀; Kisenyi; 14 Feb. 1952; A.E. Bertrand leg.; KMMA • 1♀; Rubona; 5 Apr. 1963; G. Pierrard leg.; KMMA • 1♂; Shangugu; 23 Oct.–15 Nov. 1948; F.J. François leg.; KBIN. South Africa • 1♂; Entabeni Forest Station, Zoutpansberg; Jan. 1975; B. Stuckenberg leg.; NMSA • 1♀; Schroutzich, Zoutpansberg District; 29 Mar. 1922; J. Gowdey leg.; NMSA. Tanzania • 1♀; Bunduki; 30 Apr.–2 May 1957; P. Basilewsky and N. Leleup leg.; KMMA • 1♀; Kibonoto; 4 May 1906; Sjöstedt leg.; MNHN • 1♀; Kibonoto; 2 May 1906; Sjöstedt leg.; MNB • 2♀♀; Kilema; Mar. 1912; Alluaud and Jeannel leg.; MNHN • 1♀; Tshihinda; 21–27 Aug. 1931; J. Ogilvie leg.; CNC • 1♀; Ukami; MNHN. Uganda • 1♂; SE Ankole; 4–8 Oct. 1911; S.A. Neave leg.; NHMUK • 2♂♂ 2♀♀; W Ankole; 10–14 Oct. 1911; S.A. Neave leg.; NHMUK • 1♂; Buamba Forest, Semliki Valley; 3–7 Nov. 1911; S.A. Neave leg.; NHMUK • 2♀♀; Budongo Forest, nr Lake Albert; Apr. 1972; E.B. Babyetagara leg.; CNC • 2♂♂; Diamond Trail, Ruwenzori; 6 Dec. 2018; K. Jordaens leg.; KMMA • 1♂ 1♀; Entebbe; 7–9 May 1912; C.C. Gowdey leg.; NHMUK • 1♀; Fort Portal; Jan. 1909; Ch. Alluaud leg.; MNHN • 1♀; Ruhiza, Impenetrable Forest, Kigezi; 1–10 Jun. 1972; E.B. Babyetagara leg.; CNC • 1♀; Jinja; Aug. 1928; V.G.L. van Someren leg.; NHMUK • 1♀; same collection data as for preceding; Sep. 1928 • 1♀; Kayonza Forest, Kigezi District; May 1972; H. Falke leg.; CNC • 2♂♂ 4♀♀; same collection data as for preceding; E.B. Bayetagara leg. • 3♂♂ 4♀♀; Lower Ruwenzori Mountains; 14 Apr. 1973; H. Falke leg.; CNC • 1♀; Masinde; Feb. 1912; R. Fyffe leg.; NHMUK • 1♀; Mpanga Forest; 26 Feb. 1912; J. Fraser leg.; NHMUK • 4♂♂ 5♀♀; Mt Elgon; 4–8 Dec. 1972; H. Falke leg.; CNC • 11♂♂ 17♀♀; Mt Kokanjera, SW of Elgon; 7–9 Aug. 1911; S.A. Neave leg.; NHMUK • 1♀; Namwamba Valley; Dec. 1934–Jan. 1935; F.W. Edwards leg.; NHMUK • 2♀♀; Nkokonjera; 23 Dec. 1910; C.C. Gowdey leg.; NHMUK • 1♂; NW shores Vic. Nyanza; 12–15 Sep. 1911; S.A. Neave leg.; NHMUK • 1♀; Nyabushozi, North Ankole; 21 Jan. 1975; P. Mugabi leg.; CNC • 1♀; North Ruwenzori; 1–2 Nov. 1911; S.A. Neave leg.; NHMUK • 2♂♂ 6♀♀; Ruwenzori Foothills; 3–8 Jan. 1972; H. Falke leg.; CNC • 1♂ 3♀♀; Tero Forest, SE Buddu; 26–30 Sep. 1911; S.A. Neave leg.; NHMUK • 1♂ 1♀; Usuka; 7 Dec. 1910; C.C. Gowdey leg.; NHMUK. Zambia • 1♀; Abercorn; 23 Apr. 1951; F.O. Albrecht leg.; NHMUK. Zimbabwe • 1♂; Bomponi, Vumba; 16 May 1965; D. Cookson leg.; NMSA • 1♂; Cross Kopie, Umtali; 13 Feb. 1964; D. Cookson leg.; NMSA • 1♀; Mt Selinda; 8 Feb. 1954; N.J. Myers leg.; NMSA • 1♀; Salisbury [= Harare]; 1 Jan. 1952; J.M. Brown leg.; NMSA • 1♀; Umtali; 21 Jan. 1952; N.J. Myers leg.; NMSA • 1♂; N. Vumba; 6 Nov. 1965; D. Cookson leg.; NMSA.

**Figures 40–43.**
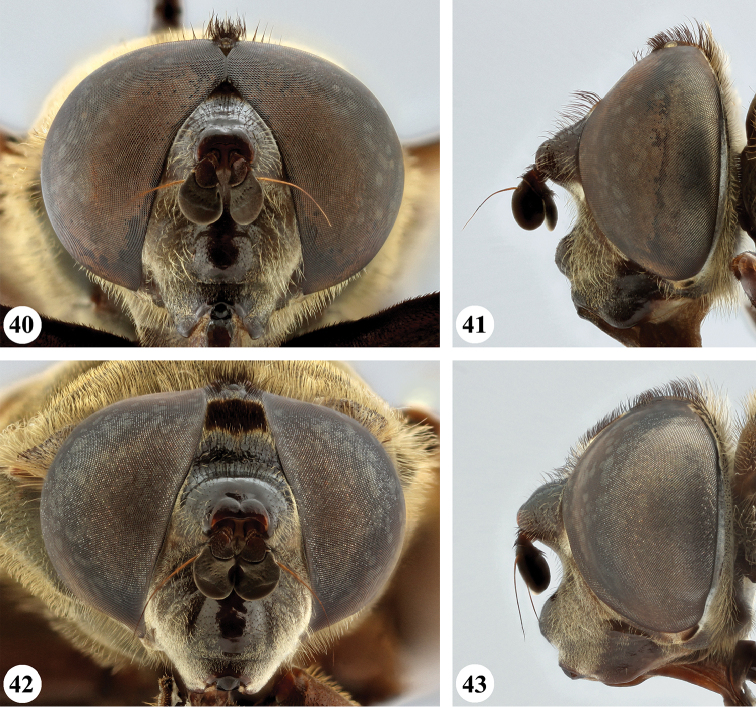
Head, frontal and lateral view S. nr umbrifera (Walker) (**40, 41** ♂ **42, 43** ♀).

#### Description.

Body length: 11.9–19.0 mm. Wing length: 8.7–13.5 mm.

**Male** (Fig. 3). Head (Figs 20, 21). Eye bare; narrowly dichoptic, eyes separated for width at most equal to one facet, narrow separation for distance shorter than length of ocellar triangle; facets dorsally slightly larger, at most twice as large in diameter as ventral ones. Frons black-brown; largely subshiny with weak greyish pollinosity along dorsal and lateral margins; dispersed short dark pile, dorsally somewhat longer, sometimes pile more pale. Face ground colour black-brown; covered with dense silvery pollinosity except facial tubercle which is bare; in parts with dispersed short pale pile; facial tubercle strongly pronounced. Gena colour and pollinosity as face; with short to long pale pile. Occiput black-brown, covered with dull grey pollinosity; with dispersed pale pile except dorsally where sometimes darker yellow. Antennal segments black-brown to black, arista orange-brown.

**Figures 44–47.**
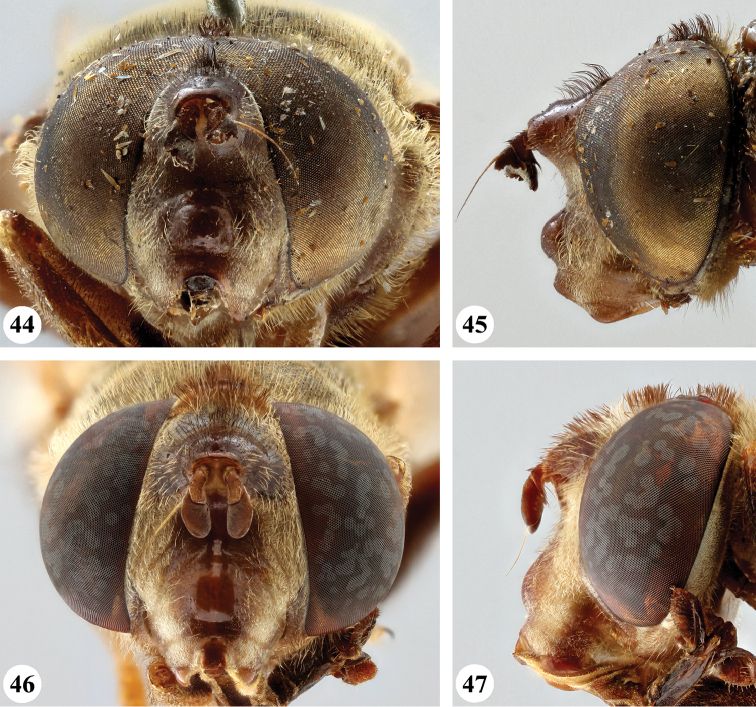
Head, frontal and lateral view **44, 45***S. umbrifera* (Walker) (♂ holotype) **46, 47***S. pennata* (Hervé-Bazin) (♀).

***Thorax*** (Figs 54, 55). Scutum and scutellum completely covered by dense pale to orange-yellow pollinosity; furthermore with dense short pile of same colour, pile longer along margins. Scutellum marginated; weakly rounded, 2.5 times wider than long. Pleura ground colour black-brown, covered with dispersed long predominantly dark pile except on meron, dorsomedial anepimeron, ventral part of katepimeron, anterior part of katepisternum and anterior anepisternum.

**Figures 48–51.**
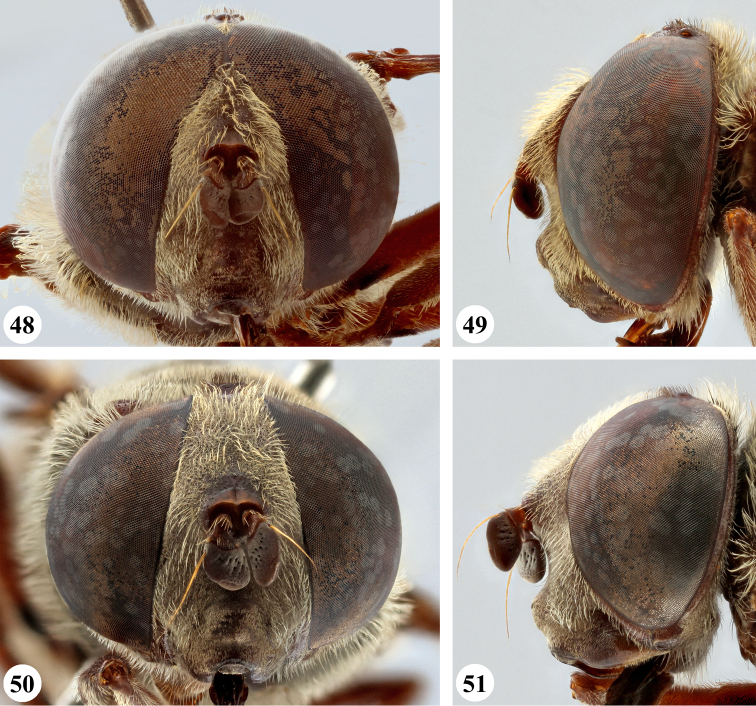
Head, frontal and lateral view *S. xanthorrhoea* (Bezzi) (**48, 49** ♂ **50, 51** ♀).

***Legs***. Black-brown, sometimes femora more red-brown; with short black pile, along posterior margin of pro- and mesofemora and ventral margin of metafemur with longer pile. Metaleg (Fig. 82), femur thickened, with slight ventral swelling in apical fifth, pile more dense where swollen; tibia thickened and curved, pile along ventral margin in apical half to two-thirds longer and more dense.

***Wing*** (Fig. 68). Brownish throughout, sometimes with purplish shine when viewed from angle; alula hyaline and posterior margin narrowly and apex broadly paler; sometimes slightly paler maculae in centre of wing cells, paler areas not distinctly demarcated and sometimes absent; if present then usually in at least cells cup, dm, r_4+5_, and r_2+3_ (Figs 69–71) (in type of var. claricella wing largely hyaline with slight fumose areas surrounding veins and cross-veins). Calypters dull chalk-white with fringe of silvery white pile; sometimes calypters and fringe more yellow. Cell r_1_ open (Fig. 69) or closed, if closed usually not distinctly petiolate (Fig. 70), rarely with very short petiole less than one third of height of stigma (Fig. 71). In type of var. claricella narrowly open. Vein R_4+5_ sinuate and usually distinctly short appendiculate, rarely without appendix.

***Abdomen*** (Fig. 93). Uniformly subshiny black to black-brown, postabdomen weakly brownish pollinose; with short dark pile except tergum 1 and postabdomen where pale pile. Sterna with long black pile. Male genitalia as in Fig. 104.

**Female.** As male except for the following character states: Eye dichoptic (Figs 22, 23), facets equal to subequal in size. Frons with brownish pollinose fascia in dorsal part for length equal to ocellar triangle, along dorsal and ventral margin of fascia narrowly greyish pollinose; frons usually with dispersed pale pile throughout, sometimes mixed with darker pile or completely black pile. Wing coloration variable, sometimes more uniformly brownish throughout but with more distinct hyaline maculae in several cells, rarely hyaline margins posteriorly broader. Cell r_1_ rarely narrowly open.

#### Distribution.

Angola, Burundi, Democratic Republic of the Congo, Ethiopia, Kenya, Malawi, Mozambique, Rwanda, South Africa, Tanzania, Uganda, Zambia, Zimbabwe.

#### Comments.

The type of the variety *claricella* corresponds with the typical *S. elliotii* in all aspects except for the wing characteristics as described above. The narrowly open cell *r_1_* was observed in other material. No other specimens with the hyaline maculae were observed in the material examined, although the extension and distinctiveness of hyaline maculae in the wing cells was observed to be variable. We suspect that it concerns a teneral specimen of *S. elliotii*.

Several authors have spelled the name of this species in variant ways, i.e., as *elliottii* (e.g., Smith and Vockeroth 1980), *ellioti* (e.g., Speiser 1910) or *elliotti* (e.g., Curran 1927). In the original description Austen (1909) specifically stated that the species is named after Mr. G.H. Scott Elliot but indicated in the text that the specimens were collected by G.F. Scott Elliot (a Scottish botanist who collected in several African countries). In both cases the name was spelled as Elliot (with single ‘t’) and Austen gave the species name as *elliotii*. This should be considered as the correct original spelling and alternative spellings should be considered as incorrect subsequent spellings according to article 33.4 of the ICZN.

### Senaspis flaviceps

Taxon classificationAnimaliaDipteraSyrphidae

Macquart, 1850

B16BD780-3811-5BCF-880F-BF78A84F3A06

Figs 4 , 24–27 , 56 , 72 , 83 , 84 , 94 , 105

Senaspis flaviceps Macquart, 1850: 438.
Protylocera apophysata Bezzi, 1915: 64. Syn. nov.

#### Differential diagnosis.

A large brown to reddish brown species (Fig. 4) differentiated from all other *Senaspis* species by conspicuous yellow face and frons (Fig. 24) (dark in all other species), and a bare katepimeron (pilose in all other species). The male metafemur bears a prominent basoventral tubercle (Fig. 83) (absent in all other species).

#### Examined material.

*Senaspis flaviceps* Macquart: ***Holotype***, male, “MUSEUM PARIS // MADAGASCAR // GOUDOT 1839” “86 // 39” “Senaspis // flaviceps. ♂. // Macq. n. g., n. sp.” “TYPE” “TYPE // Vockeroth ‘69” “HOLOTYPE” “MNHN, Paris // ED6772” [MNHN].

*Protylocera apophysata* Bezzi: ***Holotype***, male, “Protylocera // Type // apophysata // Bezzi.” “Holo- // type” “Betsileo, // Madagascar // Revd. D. Cowan. // 82.30.” “Protylocera // apophysata // n. sp.” “NHMUK010369877” [NHMUK].

#### Other material.

Madagascar • 1♀; Ahitsitondrona; Dec. 1949; J. Vadon leg.; KMMA • 1♀; Ambatosoratra, Sambava; Nov. 1960 • 2♀♀; Ambodivoangy; Aug. 1961; J. Vadon leg.; KMMA • 1♀; Atsimo, Atsinanana, 50 km S Farafangana; 24 Feb.–3 Mar. 2007; M.E. Irwin and R. Harin’hala leg.; CDFA • 1♀; Fampanambo; Jun. 1960; J. Vadon leg.; KMMA • 1♀; same collection data as for preceding; Jan. 1961 • 1♀; same collection data as for preceding; Mar. 1961 • 2♂♂ 1♀; same collection data as for preceding; 1962 • 1♂ 1♀; Fort Dauphin [= Taolagnara], Mandena; 14–18 Apr. 1968; K.M.G. and P.D. leg.; NHMUK • 1♀; Foulpointe [= Mahavelona]; Jan. 1995; A. Pauly leg.; KMMA • 1♂; same collection data as for preceding; May 1995 • 1♀; same collection data as for preceding; 25 Oct. 1995 • 1♂; Horombe, Vohibasia National Park, Adrefana; Atsimo; 9–15 Feb. 2013; M. Irwin and Rin’ha leg.; CDFA • 1♀; Ivolaina; Feb. 1960; Sigwalt leg.; MNHN • 1♀; same collection data as for preceding; Oct. 1961 • 1♂; Ivondro; Dec. 1938; A. Seyrig leg.; MNHN • 3♂♂; Mananara; Oct. 1963; J. Vadon leg.; KMMA • 1♀; Maroantsetra; J. Vadon leg.; MNHN • 1♀; Miarinarivo; 1919; E. Séguy leg.; MNHN • 1♂; Mus Pragense; CNC • 1♀; Oriental Forest, Moramanga; Jan.–May 1937; C. Lamberton leg.; CNC • 1♂ 1♀; same collection data as for preceding; May–Sep. 1938 • 1♀; Oubanghi-Chari, Fort Sibut; 1968; Breuning leg.; KMMA • 1♀; Ranomafana National Park; 1–10 Dec. 2009; M.E. Irwin and R. Harin’hala leg.; CDFA • 1♀; Ranomafana National Park; 13 Jan. 2014; M. Hauser leg.; CDFA • 1♀; Ranomafana National Park; Rogez leg.; CNC • 2♂♂; Ranomafana National Park; 21 Dec. 2017; Schmid-Egger leg.; CDFA • 1♀; Sorulirano; A. Seyrig leg.; MNHN • 1♂; Tsivory; 1906; Fauchère leg.; MNHN • 1♂ 1♀; no locality; 1937; A. Seyrig leg.; MNHN.

**Figures 52–55.**
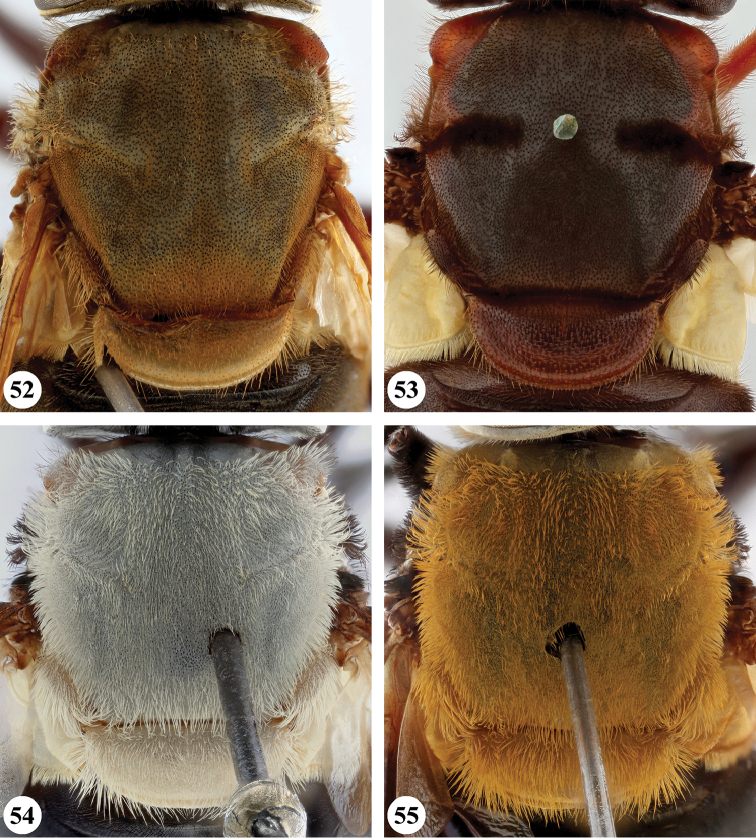
*Senaspis* species, thorax, dorsal view **52***S. dentipes* (Macquart) (♂) **53***S. dibapha* (Walker) (♂) **54, 55***S. elliotii* Austen (♂).

#### Description.

Body length: 17.5–23.8 mm. Wing length: 12.7–17.5 mm.

**Male** (Fig. 4). Head (Figs 24, 25). Eye bare; holoptic, eye contiguity for distance equal to length of ocellar triangle, facets dorsally only slightly larger, at most twice as large in diameter as ventral facets. Frons yellow; weakly subshiny, largely with yellow pollinosity; dispersed yellow pile, dorsally somewhat longer. Face yellow; subshiny with yellow pollinosity, ventral part mainly yellow-orange subshiny; in parts with dispersed long pale pile; facial tubercle moderately pronounced. Gena colour and pollinosity as ventral lateral margins of face; with short to long pale pile. Occiput orange-brown, covered with orange-brown pollinosity; with dispersed pale pile except dorsally where sometimes more rufous. Antennal segments yellow; arista yellow at base, brown apically.

**Figures 56–59.**
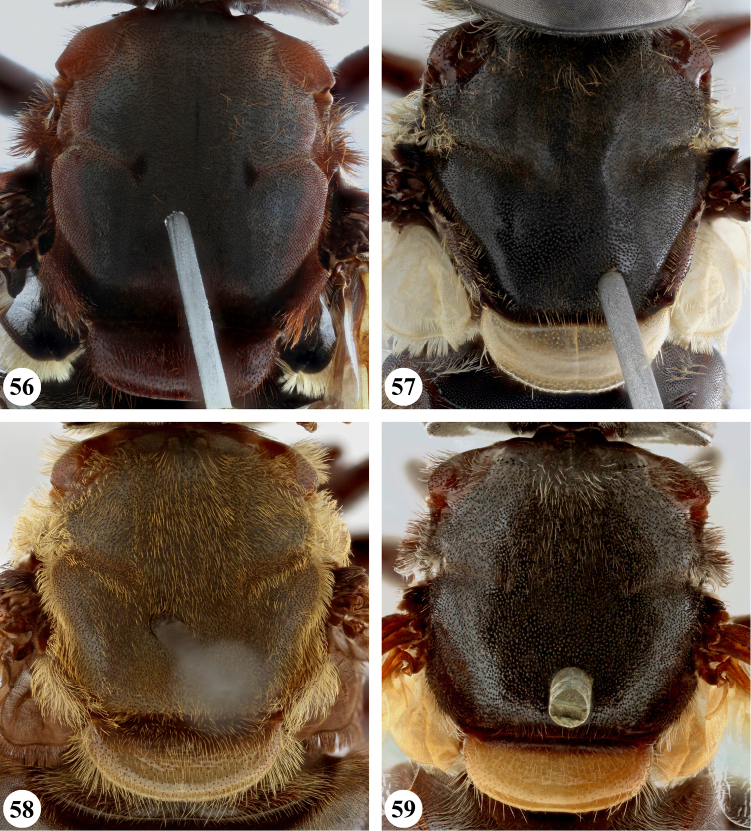
*Senaspis* species, thorax, dorsal view **56***S. flaviceps* Macquart (♂) **57***S. haemorrhoa* (Gerstaecker) (♂) **58***S. melanthysana* (Speiser) (♂) **59***S. nigrita* (Bigot) (♂).

***Thorax*** (Fig. 56). Scutum subshiny black-brown, postpronotum and lateral margin broadly rufous, narrowly along transverse suture and anterior of scutellum also rufous; with weak grey pollinosity and short rufous pile. Scutellum near to three times as wide as long, apical margin straight, not rounded; rufous; with short rufous pile. Pleura ground colour black-brown, with grey pollinosity, covered with dispersed long dark (more rufous at dorsal margin of posterior anepisternum and anterior anepimeron) pile except katepimeron, meron, dorsomedial and posterior anepimeron, anterior part of katepisternum and anterior anepisternum.

***Legs***. Brown to black-brown, femora partly more reddish brown ventrally; with short black pile, along posterior margin of pro- and mesofemora with longer pile. Metaleg (Fig. 83), femur distinctly thickened, with one distinct ventral tubercle in basal fourth to fifth, tubercle with distinct short dark pile; tibia thickened, distinctly bent at base, and only slightly curved, no longer pile along ventral margin.

***Wing*** (Fig. 72). Yellowish brown tinge especially in anterior half, in parts more black-brown; towards posterior margin and apex more hyaline. Calypters black with fringe of silvery white pile. Cell r_1_ closed, petiole at least as long as height of base of stigma. Vein R_4+5_ sinuate but not appendiculate.

***Abdomen*** (Fig. 94). Uniformly subshiny brown to reddish brown, terga IV and V and postabdomen often more rufous; with short dark pile, except tergum I where grey, and postabdomen where more rufous. Sterna dark; with long pale pile. Male genitalia as in Fig. 105.

**Female.** As male except for the following character states: Eye dichoptic (Figs 26, 27), facets of equal size. Thorax pilosity darker medially. Metafemur (Fig. 84) more slender, without ventral tubercle. Abdominal sterna IV–V with shorter dark pile.

#### Distribution.

Species described from Madagascar. Keiser (1971) also reported the species from the Comoros (Grande Comore).

#### Comments.

The type material of *S. flaviceps* and *S. apophysata* was compared and considered to be conspecific. *Senaspis apophysata* is thus considered as a junior synonym of *S. flaviceps*. This synonymy was already suggested (Ssymank et al. in press, F.C. Thompson pers. comm.) but not formally published. The original description of *S. flaviceps* mentions the type locality as “De l’Afrique. Cap?” but the type specimen bears a label indicating “Madagascar” // “Goudot 1839”. All studied material originates from Madagascar and no specimens collected on the African mainland were found. We, therefore, consider the current distribution of this species to be limited to Madagascar (and possibly Comoros; see Keiser 1971). *Senaspis flaviceps* differs from all other species within the genus in some aspects, such as the bare katepimeron (pilose in the rest, albeit weakly so), the presence of a basal tubercle in the male metafemur (absent in all others), the relatively longer petiole (at least as long as height of base of stigma, shorter in all others), and the larger body size (larger than 17 mm and up to 23 mm).

### Senaspis haemorrhoa

Taxon classificationAnimaliaDipteraSyrphidae

(Gerstaecker, 1871)

15AB87D9-805D-50F1-AAB8-4C2372E70F27

Figs 5 , 28–31 , 57 , 73 , 85 , 95 , 106

Plagiocera haemorrhoa Gerstaecker, 1871: 363.
Dolichomerus griseifacies Bezzi, 1908: 381. Syn. nov.

#### Differential diagnosis.

A species with a distinct medial dark brown macula on the wing (Fig. 73). It can be differentiated from other *Senaspis* species with a distinct wing macula by the coloration of the abdomen: largely brown with the posterior terga conspicuously orange to orange-red and with red pile (Fig. 95) (without red pile in *S. dentipes* (Fig. 91); red coloration and pile more extensive in *S. xanthorrhoea* (Fig. 100)).

#### Type.

*Plagiocera haemorrhoa* Gerstaecker: Syntypes, male, female [number unknown], TANZANIA, Wanga [not examined, institutional depository unknown].

#### Examined material.

*Dolichomerus griseifacies* Bezzi: ***Lectotype*** (hereby designated), male, “Senaspis // haemorrhoa / det. C. Kassebeer 1996” “Dolichomerus // griseifacies // ♂ n.sp.” “Cf. Ann. Soc. Ent. Belg // Vol. 52 (1908) p. 381” “Ex-typis” “M. Bezzi det., 1908 : // Dolichomerus // griseifascies Bezzi” “Moliro // Mars–Mai 95 //J. Duvivier” “♂” ”LECTOTYPUS” [KBIN]. Paralectotype, male, “Cf. Ann. Soc. Ent. Belg // Vol. 52 (1908) p. 381” “Ex-typis” “M. Bezzi det., 1908: // Dolichomerus // griseifascies Bezzi” “Moliro // Mars–Mai 95 //J. Duvivier” “♂” “PARA- // LECTOTYPUS” [KBIN]. Paralectotype, male, “Cf. Ann. Soc. Ent. Belg // Vol. 52 (1908) p. 381” “Ex-typis” “M. Bezzi det., 1908: // Dolichomerus // griseifascies Bezzi” “Moliro // Mars–Mai 95 //J. Duvivier” “♂” “PARA- // LECTOTYPUS” [KBIN].

**Figures 60–63.**
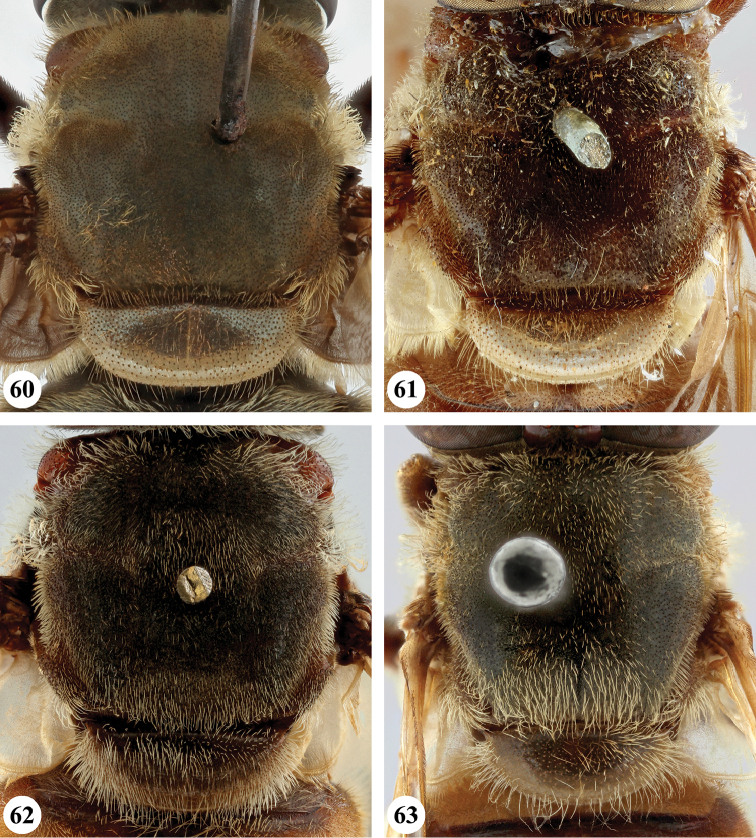
*Senaspis* species, thorax, dorsal view **60**S. nr umbrifera (Walker) (♂) **61***S. umbrifera* (Walker) (♂ holotype) **62***S. xanthorrhoea* (Bezzi) (♂) **63***S. pennata* (Hervé-Bazin) (♀).

#### Other material.

Angola • 2♂♂; 12 mi SW Luimbale; 20–21 Mar. 1972; NHMUK • 2♀♀; Luanda; Jun. 1957–Jul. 1958; G. Heinrich leg.; CNC • 1♀; Roçada; 30 Feb. 1972; NHMUK. Benin • 1♀; Cotonou; 3 Jun. 1914; W.A. Lamborn leg.; NHMUK • 7♂♂; Porto Novo; 7 Mar. 2018; K. Jordaens leg.; KMMA • 1♂ 1♀; Sémé; Nov. 2019; G. Goergen leg.; IITA. Botswana • 1♂; 20 km W Chonzi; 28 Aug. 1983; C. Stockmann leg.; NMSA • 1♀; Molepolole District; 3 Mar. 1963; T. Oatley leg.; NMSA • 1♀; Serowe; 14 Apr. 1983; P. Forchhammer leg.; NMSA. Burundi • 2♂♂; Bubanza, Gihanga Hill, Ruzizi; 23 Nov. 1951; F.J. François leg.; KBIN • 1♂; Bujumbura; 26 Feb. 1979; J. Decelle leg.; KMMA • 1♂ 1♀; Bujumbura; 21 Feb. 2017; G. Goergen leg.; IITA • 2♀♀; Kanyinya; Dec. 1947–Jan. 948; Dames de Marie leg.; KMMA • 1♂; Kitega; 3 Dec. 1950; F.J. François leg.; KBIN • 1♂; same collection data as for preceding; 1 May 1952 • 4♂♂ 3♀♀; same collection data as for preceding; 19 May 1955 • 1♀; same collection data as for preceding; 12 Jun. 1957 • 1♀; 11 Dec. 1962; M. Fontaine leg.; KMMA • 1♂; Murehe; 19 [?] 2013; S. Girukwishaka leg.; OBPE • 1♂; Rusizi N P; 26 [?] 2013; S. Girukwishaka leg.; OBPE • 1♀; Musuma, Bututsi; 29 Nov. 1950; F.J. François leg.; KBIN • 1♂ 9♀♀; Nyakibande, Mumirwa; 24 Apr. 2019; E. Sinzinkayo leg.; OBPE • 4♂♂ 9♀♀; same collection data as for preceding; 14 Jul. 2019 • 1♀; Nyambuye; Mumirwa; 18 Mar. 2019; E. Sinzinkayo leg.; OBPE • 1♂ 18; same collection data as for preceding; May 2019 • 6♀♀; same collection data as for preceding; 18 Sep. 2019 • 1♀; Rumonge; 20 Apr. 1948; • 1♀; same collection data as for preceding; 19–20 Jun. 1948 • 1♂; same collection data as for preceding; Jul. 1948 • 1♀; same collection data as for preceding; May 1949 • 1♀; 19 Feb. 1950; all F.J. François leg.; KBIN • 5♂♂ 20♀♀; Rwegura, Kibira NP; 31 May 2018; E. Sinzinkayo leg.; OBPE • 1♀; Rusinga NP, secteur Delta; 18 May 2018; E. Sinzinkayo leg.; OBPE • 2♂♂ 10♀♀; same collection data as for preceding; 19 May 2018 • 1♂; same collection data as for preceding; 20 May 2018 • 1♂ 4♀♀; same collection data as for preceding; 24 May 2018 • 1♀; Usumbura; Sep. 1961; G. Pierrard leg.; KMMA. Comoros • 1♂ 1♀; Le Galawa, Grande Comore; 22 Apr.–5 May 1991; K.M. Guichard leg.; NHMUK • 1♀; M’Vouni, Grande Comore; 23 Apr.–3 May 1991; K.M. Guichard leg.; NHMUK • 1♂ 1♀; Moroni, Grande Comore; Malet leg.; MNHN • 1♀; Grande Comore; 1899; MNHN. Democratic Republic Of The Congo • 3♀♀; Albertville [= Kalemie]; 14 Aug. 1953; J. Verbeke leg.; KBIN • 1♀; same collection data as for preceding; 17 Aug. 1953 • 1♀; same collection data as for preceding; 24 Aug. 1953 • 1♀; same collection data as for preceding; Jul. 1955; H.E. Bomans leg.; KBIN • 1♂; same collection data as for preceding; Dec. 1918; R. Mayné leg.; KMMA • 1♂; same collection data as for preceding; 1–20 Jan. 1919 • 1♂; same collection data as for preceding; 7 Feb. 1933; L. Burgeon leg.; KMMA • 1♀; Bendera; Oct. 1958; N. Leleup leg.; KMMA • 1♀; Baudouinville [= Kirungu]; May 1953; H. Bomans leg.; KMMA • 1♂; Bukama; 18 Apr. 1911; Bequaert leg.; KMMA • 1♂; same collection data as for preceding; 4 Jun. 1911 • 1♀; 3 Aug. 1923; Ch. Seydel leg.; KMMA • 1♀; Buta; 1949; R.F. Hutsebaut leg.; KMMA • 3♂♂; Chamunyonge; Lake Edward; 26 Sep. 1953; F.J. François leg.; KBIN • 2♂♂ 4♀♀; Costermansville [= Bukavu]; 1948; P.H. Vercammen leg.; KMMA • 1♂; Costermansville [= Bukavu]; 13 May 1949; H. Bomans leg.; KMMA • 1♀; Elisabethville [= Lubumbashi]; May 1964; E. Coussement leg.; KBIN • 2♂♂; Elisabethville [= Lubumbashi]; M. Bequaert leg.; KBIN • 1♂; Elisabethville [= Lubumbashi]; Nov. 1919; KMMA • 6♂♂; Elisabethville [= Lubumbashi]; 19 Sep. 1919; J. Ghesquière leg.; KMMA • 1♀; Elisabethville [= Lubumbashi]; 6 May 1920; M. Bequaert leg.; KMMA • 1♀; same collection data as for preceding; 3 Jun. 1920 • 2♂♂; same collection data as for preceding; 1 Jan. 1921 • 1♀; same collection data as for preceding; 5 Jan. 1921 • 1♂; same collection data as for preceding; 7 Jan. 1921 • 1♀; same collection data as for preceding; 16 Jan. 1921 • 1♂; same collection data as for preceding; 14 Feb. 1921 • 1♂; same collection data as for preceding; 1 Jul. 1923 • 1♂; same collection data as for preceding; 2 May 1923 • 1♀; same collection data as for preceding; Nov. 1929 • 1♀; same collection data as for preceding; Dec. 1939 • 1♀; same collection data as for preceding; Dec. 1928; Ch. Seydel leg. • 1♀; 1928–1929; same collection data as for preceding; P. Quarré leg. • 1♂; Elisabethville/Lubumbashi; 5 Jan. 1920; M. Bequaert leg.; NHMUK • 1♂; same collection data as for preceding; 23 Dec. 1920 • 1♀; same collection data as for preceding; 5 Jan. 1921 • 1♀; Goma; 1 May 1953; KBIN • 1♂; Goma; 18 Nov. 1952; J. Verbeke leg.; KBIN • 1♀; same collection data as for preceding; 10–15 May 1953 • 1♀; Ibembo; Uelé; Oct. 1949; R.F. Hutsebaut; KMMA • 1♀; same collection data as for preceding; 20 May 1950 • 1♀; same collection data as for preceding; Apr. 1952 • 1♀; same collection data as for preceding; 1953 • 1♀; Jadotville [= Likasi]; 18 Aug. 1965; E. Coussement leg.; KBIN • 1♀; Jampwe; 1912; Van den Heuvel leg.; KMMA • 1♀; Kabinda, Lomami; KBIN • 1♀; Mt Kabobo, Hte Kiymbi; Sep. 1958; N. Leleup leg.; KMMA • 1♀; Kakanda, Mutaka; 1955; R.P. Th de Caters leg.; KMMA • 1♀; Kamaniola; 1 Feb. 1927; J. Bequaert leg.; KMMA • 1♂; Kambove, Katanga; 11 Jun. 1907; S.A. Neave leg.; NHMUK • 1♀; Kasongo; Sep. 1959; P.L.G. Benoit leg.; KMMA • 4♀♀; Pons leg.; KMMA • 1♀; Kiambi; 27 Apr. 1931; G.F. de Witte leg.; KMMA • 1♂; Kigoma; Jul. 1918; R. Mayné leg.; KMMA • 1♀; same collection data as for preceding; Sep. 1918 • 1♂; Kindu; 15 Nov. 1911; Rodhain leg.; KMMA • 1♀; Kindu (300 km from) Russo; KMMA • 1♂; Kivu; Rodhain leg.; KMMA • 2♂♂ 1♀; Kolwezi; Lualaba; 1–6 Nov. 1952; L. Gilbert leg.; KMMA • 1♀; same collection data as for preceding; 13 Nov. 1952 • 1♀; same collection data as for preceding; 1 Feb. 1953 • 1♀; same collection data as for preceding; 5 Feb. 1953 • 1♀; Kongolo; 6 Feb. 1974; R. Baker leg.; NHMUK • 1♀; Kulu-Mwanza; Katanga; May 1927; A. Bayet leg.; KMMA • 1♀; Kunda; 23 Nov. 1910; Bequaert leg.; KMMA • 1♂; Lubongola; 1939; Hautmann leg.; KMMA • 1♀; same collection data as for preceding; May 1939 • 1♂; Lubumbashi; 5 Jan. 1921; M. Bequaert leg.; KMMA • 1♀; Lubumbashi; 2 Jan. 1968; A.B. Stam leg.; KMMA • 1♂; Lubutu; 22 Jan. 1915; J. Bequaert leg.; KMMA • 1♀; Luluabourg [= Kananga], Kabure; 1937; Soeurs du Carmel leg.; KMMA • 1♀; Luputa, Lomami; Sep. 1934; Bouvier leg.; KMMA • 1♀; same collection data as for preceding; Nov. 1934 • 1♀; same collection data as for preceding; 1935 • 1♀; Lusinga, P.N. Upemba; 10 Apr. 1947; G.F. de Witte leg.; KBIN • 1♀; same collection data as for preceding; 24 Apr. 1947; KMMA • 1♀; same collection data as for preceding; 26 Apr. 1947 • 1♂; Luvungi, North Kivu; 12 Dec. 1932; L. Burgeon leg.; KMMA • 4♂♂; Malondo, Katanga; Ch. Seydel leg.; KMMA • 1♂; Matale; Jul. 1939; Hautmann leg.; KMMA • 1♀; Moba, Tanganyika; Jun. 1931; G.F. de Witte leg.; KMMA • 1♀; Moba, Tanganyika; Jun. 1953; H. Bomans leg.; KMMA • 2♀♀; same collection data as for preceding; Aug.–Oct. 1953 • 2♀♀; Mpala, Tanganyika; H. Bomans leg.; KMMA • 1♀; Nyangwe; 15 Nov. 1910; Bequaert leg.; KMMA • 1♂ 1♀; Nyangwe; Apr.–May 1918; R. Mayné leg.; KMMA • 1♀; Nyonga, Katanga; 18 May 1925; G.F. de Witte leg.; KMMA • 1♀; Paulis; 29 Oct. 1959; M. Fontaine leg.; KMMA • 1♂ 1♀; Stanleyville [= Kisangani]; Apr. 1915; Lang and Chapin leg.; CNC • 1♀; same collection data as for preceding; Feb. 1915; KMMA • 17♂♂ 28♀♀; same collection data as for preceding; Mar. 1915 • 2♀♀; same collection data as for preceding; 1 Apr. 1915 • 1♂ 1♀; same collection data as for preceding; 2 Apr. 1915 • 1♀; same collection data as for preceding; 4 Apr. 1915 • 1♀; same collection data as for preceding; 5 Apr. 1915 • 2♂♂ 1♀; same collection data as for preceding; 6 Apr. 1915 • 2♂♂ 8♀♀; same collection data as for preceding; 7 Apr. 1915 • 4♀♀; same collection data as for preceding; 14 Apr. 1915 • 1♀; same collection data as for preceding; Apr. 1915 • 2♂♂ 3♀♀; same collection data as for preceding; 7 Jul. 1915 • 1♀; same collection data as for preceding; 1915; J. Bequaert leg.; KMMA • 1♀; same collection data as for preceding; 5 May 1926; H. Schouteden leg. • 1♀; same collection data as for preceding; 13 Aug. 1928; A. Collart leg. • 1♀; same collection data as for preceding; 6 Sep. 1928 • 1♂; same collection data as for preceding; 1929; J. Muller leg.; KBIN • 1♀; same collection data as for preceding; 28 Apr. 1932; J. Vrydagh leg.; KMMA • 1♀; Uvira; Jul. 1927; Ch. Seydel leg.; KMMA • 1♀; Uvira; Aug. 1949; G. Marlier leg.; KMMA • 1♀; Vieux-Kasongo; Dec. 1910; Pons leg.; KMMA. Ethiopia • 1♂; Abijata Shala National Park, Oromia; 1–10 Oct. 2012; A. Pauly leg.; KMMA • 1♂ 1♀; Senkele National Park, Oromia; 17 Oct. 2012; A. Pauly leg.; KMMA. Guinea Bissau • 2♀♀; Bolama; Jun.–Dec. 1899; L. Fea leg.; MCSNG. Kenya • 2♀♀; Bungoma, Western Province; 29 Oct. 1968; J. Field leg.; CDFA • 1♀; Funzi Island; 9 Jul. 2012; ICIPE • 1♂; Gede National Park, Malindi; 1 May 1973; H. Falke leg.; CNC • 1♀; Kibwezi; 6 Jan. 1926; W. Feather leg.; NHMUK • 7♀♀; same collection data as for preceding; 24 Dec. 1927 • 4♀♀; same collection data as for preceding; 24 Jan. 1928 • 1♀; same collection data as for preceding; 9 Feb. 1928 • 1♀; Kisumu; Nov. 1932; V.G.L. van Someren leg.; NHMUK • 2♀♀; Lake Mpeketoni near Kipini; 4–5 Mar. 1912; S.A. Neave leg.; NHMUK • 1♀; Loyia Turkana; 20 Aug. 1954; NHMUK • 1♀; Magadi; Jul. 1941; V.G.L. van Someren leg.; NHMUK • 1♀; Mombasa; Jul. 1904; Ch. Alluaud leg.; MNHN • 1♀; Nairobi; 14 Dec. 1951; L.C. Edwards leg.; NHMUK • 1♀; Nanyuki; Jul. 2010; M. Roberts leg.; ICIPE • 1♂; New Runda; 22 Apr. 2012; F. Haas leg.; ICIPE • 2♀♀; Rabai; May 1928; V.G.L. van Someren leg.; NHMUK • 2♂♂; same collection data as for preceding; Jul. 1928 • 1♂; Tsavo East; 6 Jan. 1989; A.u.G. Rautenstrauch leg.; CDFA • 1♀; Uchweni Forest; 1–3 Mar. 1912; S.A. Neave leg.; NHMUK • 1♂ 2♀♀; Ziwani; Aug. 1947; V.G.L. van Someren leg.; NHMUK. Malawi • 1♀; 30 km NW of Dedza; 15 Mar. 1987; J. and A. Londt leg.; NMSA • 3♀♀; Kasungu; 9 Dec. 1980; B. Stuckenberg and J. Londt leg.; NMSA • 1♂; Livingstonia; 3 May 1939; R.C. Wood leg.; NHMUK • 1♀; Mulanje Mountain Forest Reserve; 12–15 Nov. 2016; K. Jordaens leg.; KMMA • 1♂; Ruo; 4 Feb. 1916; R.C. Wood leg.; NHMUK • 1♂ 1♀; Viphya Mountains, Chikangawa; 5–8 Dec. 1980; B. Stuckenberg and J. Londt leg.; NMSA. Mozambique • 1♀; Chiramba, Zambeze; 1929; P. Lesne leg.; MNHN • 1♀; Lourenço Marques [= Maputo]; 1909; J. de O.S. de Azevedo leg.; NHMUK • 2♂♂ 2♀♀; Lourenço Marques [= Maputo]; Sep.–Dec. 1913; H.A. Junod leg.; NHMUK • 1♂; same collection data as for preceding; Jan.–Mar. 1914 • 1♀; Luabo, Lower Zambesi River; Jun.–Jul. 1957; CNC • 1♀; same collection data as for preceding; Aug. 1957 • 2♀♀; Luabo; U. Stuckenberg leg.; NMSA • 2♂♂; Manga, Beira Region; 27 Jul. 1929; P. Lesne leg.; MNHN • 1♂; Revoué Valley, Andrada envir.; Apr.–May 1905; G. Vasse leg.; MNHN • 1♀; Rikatla; H.A. Junod; NMSA • 1♀; Siluwe Hills, W. of Beira; 3 Jun. 1964; D. Cookson leg.; NMSA • 1♀; Tumbine Mountains, Milange District; Apr. 1958; B. and P. Stuckenberg leg.; NMSA. Namibia • 3♀♀; Kombat; 1–6 Apr. 1972; NHMUK • 1♀; between Omaruru and Wilhelmstad; 3 Apr. 1998; F.W. and S.K. Gess leg.; AMGS • 1♀; WNW of Omatjete; 15 Mar. 2004; F.W. and S.K. Gess leg.; AMGS • 1♂; Oranjemund, 28 km from checkpoint on road to Sendelingsdrift; 25 Sep. 1997; F.W. and S.K. Gess leg.; AMGS • 1♀; Rietfontein, 23 mi W SW Grootfontein; 3 Apr. 1972; NHMUK • 1♀; 24 km SE Stampriet, Gross Nabas; 30 Mar. 2000; F.W. and S.K. Gess leg.; AMGS • 1♀; 71 km East Stampriet; 23 Mar. 2000; F.W. and S.K. Gess leg.; AMGS • 1♂; 71 km West Witvlei; 17 Mar. 1984; J. Londt and B. Stuckenberg leg.; NMSA. Nigeria • 1♀; 23 km W Lagos; 11 May 1975; J. Riley leg.; NHMUK. Republic Of The Congo • 1♀; Brazzaville env.; 1907; E. Roubaud and A. Weiss leg.; MNHN. Rwanda • 1♀; Astrida [= Butare]; 22 Feb. 1953; P. Basilewsky leg.; KMMA • 1♀; same collection data as for preceding; 5 Mar. 1955; P. Elsen leg.; • 1♀; same collection data as for preceding; 15 Mar. 1955 • 1♀; Karissimbi; Dec. 1925; H. Schouteden leg.; KMMA. São Tomé and Príncipe • 3♂♂ 2♀♀; São Tomé, Ribeira Palma; Aug. 1900; L. Fea leg.; MCSNG • 3♀♀; Príncipe, Roca Inf. D. Henrique; Mar. 1901; L. Fea leg.; MCSNG • 1♀; 1900; A. Negreiros leg.; MNHN • 1♀; 1919–1921; H.J. Snell leg.; NHMUK • 2♂♂ 1♀; 1920; H. Navel leg.; MNHN • 1♀; 19 Sep. 1921; H.J. Snell leg.; NHMUK • 1♀; no data; H. De Saeger and Prince Leopold leg.; KMMA. Somalia • 1♀; Mogadishu; 20 Feb. 1953; NHMUK. South Africa • 1♀; Amatikulu Nature Reserve; 14 Jun. 2011; J. Londt and T. Dikow leg.; NMSA • 4♀♀; Ben Lavin Nature Reserve; 13 Feb. 2005; J. Londt and T. Dikow leg.; NMSA • 1♂; Bluff, Durban; 8 Mar. 1925; NMSA • 1♀; Blyderivierspoortdam Nature Reserve; 25–26 Oct. 1984; C.D. Eardley leg.; NMSA • 1♀; Empangeni; 7 Apr. 1989; P. Reavell leg.; NMSA • 1♀; False Bay; 24 Feb.–4 Mar. 1990; A.J. Weaving leg.; NMSA • 1♀; Gillits; 24 Feb. 1957; NMSA • 1♀; Ingwavuma; 10 Dec. 1963; B. and P. Stuckenberg leg.; NMSA • 1♀; Irene; 24 May 1971; C.K. Brain leg.; NMSA • 1♂; Letaba Camp, Kruger National Park; 14–18 Nov. 1961; Vari and Rorke leg.; NMSA • 2♀♀; 37 km N Louis Trichardt, Limpopo; Jan. 1975; B. Stuckenberg leg.; NMSA • 2♂♂ 4♀♀; Mafikeng Game Reserve, Kolobe drinking pond; 16 Mar. 2003; J.Londt leg.; NMSA • 1♀; Mafikeng Game Reserve, Noka picnic site; 17 Mar. 2003; J. Londt leg.; NMSA • 1♀; Malvern; Natal; Jun. 1897; G.A.K. Marshall leg.; NHMUK • 1♀; Mntunzini, Garland Farm, Natal; 1 Aug. 1985; P. Atkinson leg.; NMSA • 1♀; Modimolle, Limpopo; 20 Nov. 2017; A. Vujic and E. Rättel leg.; MZH • 1♂ 2♀♀; Mpenjati Nature Reserve; 22–23 Jan. 2011; J. and A. Londt leg.; NMSA • 1♀; Ndumu Game Reserve, staff house; 15–17 Feb. 2011; J. Londt leg.; NMSA • 1♀; Ndumu Game Reserve, rest camp; 23–29 Nov. 1977; D. Brothers and J. Guillarmod leg.; NMSA • 1♀; Port St John Pondoland; 1–15 Apr. 1924; B.E. Turner leg.; NHMUK • 1♀; 5 km E Sabie, Bergvliet Road; 24 Feb. 1971; B. Stuckenberg leg.; NMSA • 1♂ 1♀; San Lameer resort area; 11–12 Feb. 2012; J. and A. Londt leg.; NMSA • 1♀; Shelly Beach; 29 May 1984; D. Wheeler leg.; NMSA • 1♀; 4 km E Skukuza, Sabie River, Kruger National Park; 4 Jan. 1974; B. and P. Stuckenberg leg.; NMSA • 1♀; Tshifhire, Venda; Jul. 1983; Neluheni leg.; NMSA • 1♀; Umbilo; 21 Mar. 1915; L. Bevis leg.; NMSA • 2♂♂ 1♀; Umtamvuna Nature Reserve; 14 Jan. 1981; J. Londt leg.; NMSA • 1♂; same collection data as for preceding; 29 Oct. 1990; A. Whittington leg. • 1♀; van Reenen’s Pass, Ladysmith, Kwa-Zulu Natal; 23 Feb. 2016; G. Ståhls and E. Rättel leg.; MZH • 1♀; Vernon Crookes Nature Reserve; 16 Nov. 2003; G.B.P. Davies leg.; NMSA. Tanzania • 1♀; Amani; 1957; J.G. Halcrow leg.; NHMUK • 1♀; Chenzema, Uluguru Mountains; 2–22 Jul. 1971; L. Berger; N. Leleup and J. Debecker leg.; KMMA • 1♂; Dar es Salaam; Apr. 1961; G. Heinrich leg.; CNC • 1♀; same collection data as for preceding; 12 Sep. 1961 • 1♀; same collection data as for preceding; 14 Sep. 1961 • 11♂♂ 1♀; same collection data as for preceding; Oct. 1961 • 1♂; same collection data as for preceding; 20 Oct. 1961 • 2♂♂; Dodoma; Nov. 2008; G. Goergen leg.; IITA • 2♂♂; same collection data as for preceding; 27 Nov. 2008 • 8♂♂; Iringa-Morogoro; 23 Nov. 2008; G. Goergen leg.; IITA • 1♀; Kunduchi; Jul. 1973; CDFA • 1♀; Lake Manyara; Jun.–Aug. 1937; B. Cooper leg.; NHMUK • 1♀; Matumbi Highlands; 25 Nov. 1989; W.R.B. Hynd leg.; NHMUK • 1♀; Morogoro; 6 May 1925; A.H. Ritchie leg.; NHMUK • 1♂ 1♀; same collection data as for preceding; 20 May 1925 • 2♀♀; same collection data as for preceding; 10 Jun. 1925 • 1♀; nr Morogoro, Uluguru Mountains; Jan. 1962; CNC • 1♂; Mungula Gorge, Bunduki, Uluguru Mts; P. Basilewsky and N. Leleup leg.; KMMA • 1♂; Musoma; 29 Nov. 2008; G. Goergen leg.; IITA • 2♂♂; Mwanza; 30 Nov. 2008; G. Goergen leg.; IITA • 1♂; Njombe; 23 Oct. 1951; W. Peters leg.; NHMUK • 1♀; same collection data as for preceding; 9 Dec. 1952 • 1♂; same collection data as for preceding; 23 Oct. 1957 • 1♂; Old Shinyanga; 26 Mar. 1953; E. Burtt leg.; NHMUK • 1♂; same collection data as for preceding; 28 Mar. 1953 • 1♂; same collection data as for preceding; 10 Apr. 1953 • 1♂; same collection data as for preceding; 11 Apr. 1953 • 1♂; Rukwa Valley; 16 Sep. 1952; NHMUK • 2♀♀; Tanga; Apr.–May 1950; R.C.H. Sweeney leg.; NHMUK • 3♂♂; Ukara Island; Lake Victoria; 1953; A. Smith leg.; NHMUK • 1♀; Zanzibar; [no date]; ex coll Bigot; NHMUK • 2♀♀; Zanzibar; C. Cooke leg.; CNC • 1♂; Zanzibar; 17 May 1911; W.M. Aders leg.; NHMUK • 1♂; Zanzibar; Jan.–Feb. 1925; H.J. Snell leg.; NHMUK. Togo • 1♂; Kloto; 3 Mar. 2018; K. Jordaens leg.; KMMA. Uganda • 1♂ 1♀; Budongo Forest, nr Lake Albert; 1 Apr. 1972; E.B. Babyetagara leg.; CNC • 1♂ 1♀; Entebbe; May 1906; M. de Rothschild leg.; MNHN • 1♀; Entebbe; 20 Mar. 1911; C.C. Gowdey leg.; NHMUK • 1♀; same collection data as for preceding; 17 Aug. 1911 • 2♂♂ 10♀♀; same collection data as for preceding; 21 Aug. 1911 • 1♀; same collection data as for preceding; 1–11 Sep. 1911 • 2♂♂ 1♀; same collection data as for preceding; 7–9 May 1912 • 1♀; same collection data as for preceding; 27 May 1912 • 5♂♂ 3♀♀; same collection data as for preceding; 3 Sep. 1912 • 2♀♀; same collection data as for preceding; 16 Oct. 1912 • 1♀; same collection data as for preceding; 14 Nov. 1912 • 1♂; same collection data as for preceding; 18–20 Nov. 1912 • 2♀♀; same collection data as for preceding; 3–4 Dec. 1912 • 1♂ 1♀; same collection data as for preceding; 12–13 Dec. 1912 • 1♀; same collection data as for preceding; 31 Dec. 1912 • 1♀; same collection data as for preceding; 20 Mar. 1913 • 5♀♀; same collection data as for preceding; 1913 • 1♀; same collection data as for preceding; 6–13 May 1912; C.A. Wiggins leg. • 1♀; Entebbe; 1 Feb. 1972; H. Falke leg.; CNC • 2♂♂; same collection data as for preceding; 20 Nov. 1972–22 Jan. 1973 • 2♂♂; same collection data as for preceding; 25–28 Jan. 1973 • 1♀; 7 mi N Entebbe; 31 Mar. 1972; H. Falke leg.; CNC • 3♂♂ 3♀♀; near Entebbe; 23–31 Jan. 1973; H. Falke leg.; CNC • 1♂; Kayonza Forst, Kigezi; 1 Sep. 1972; H. Falke leg.; CNC • 1♀; Masindi; Aug. 1945; P.A. Buxton leg.; NHMUK • 1♀; Ruwenzori; 17 May 1911; C.C. Gowdey leg.; NHMUK • 1♀; Sukulu; 26 Jan. 1954; NHMUK • 1♀; Sukulu; 9 Apr. 1961; E. Burtt leg.; NHMUK • 2♂♂ 1♀; same collection data as for preceding; 26 Jan. 1962 • 1♀; same collection data as for preceding; 31 Jan. 1962 • 1♂; Tero; 14 Apr. 1911; C.C. Gowdey leg.; NHMUK • 1♂; Tororo; 30 Jan. 1954; NHMUK. Zambia • 2♂♂; Abercorn; 17 Jan. 1951; F.O. Albrecht leg.; NHMUK • 1♀; same collection data as for preceding; 12 Feb. 1951 • 1♂; same collection data as for preceding; 26 Feb. 1951 • 1♀; Jan. 1966; H. Mathes leg.; KMMA • 1♀; Broken Hill; 1 May 1912; E.A. Copeman leg.; NHMUK • 1♀; same collection data as for preceding; 29 May 1912 • 1♀; Mwinilunga; 9 Apr. 1914; F.V. Bruce Miller leg.; NHMUK • 1♀; Ndola; 31 Jan. 1980; T. Grout leg.; NMSA • 1♀; Nyamkolo; 9 May 1930; A. Loveridge leg.; CNC. Zimbabwe • 1♀; Banket; Dec. 1950; A. Carnegie; NMSA • 1♀; Bazely Bridge, Mutare; 14 Apr. 1964; D. Cookson leg.; NMSA • 1♀; Bomponi; 13 May 1963; D. Cookson leg.; NMSA • 1♂; same collection data as for preceding; 16 Jun. 1965 • 1♀; Chigudu Farm, Mvurwi; 14 Jan. 1985; A.J. Weaving leg.; NMSA • 1♂; Melsetter District; 23 Nov. 1993; E. Bruce-Miller leg.; AMGS • 1♂; Salisbury [= Harare]; 1 Jan. 1952; J.M. Brown leg.; NMSA • 1♀; same collection data as for preceding; 22 Apr. 1978; P.E. Hulley leg. • 1♀; Vumba Mt; March 1938; NHMUK • 1♀; East Vumba; 1 Jun. 1964 • 1♂; North Vumba; 27 Mar. 1964; D. Cookson leg.; NMSA • 1♀; same collection data as for preceding; 16 Nov. 1964.

**Figures 64–67.**
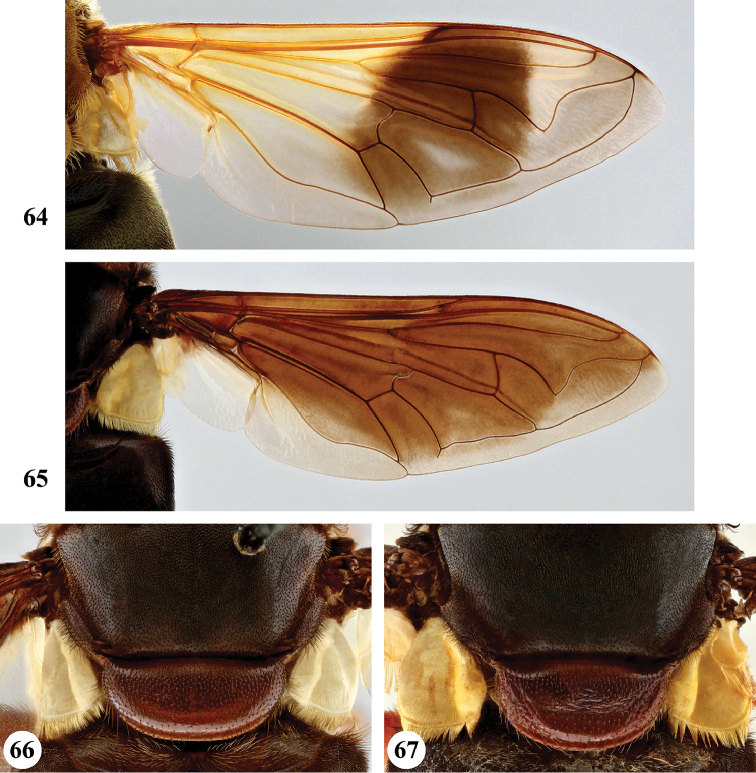
*Senaspis* species, right wing **64***S. dentipes* (Macquart) (♂) **65***S. dibapha* (Walker) (♂) **66, 67***S. dibapha* (Walker) close-up calypters.

#### Description.

Body length: 11.0–15.8 mm. Wing length: 8.0–11.0 mm.

**Male** (Fig. 5). Head (Figs 28, 29). Eye bare; holoptic, eye contiguity for distance equal to or slightly longer than length of ocellar triangle, facets dorsally slightly larger, at most twice as large in diameter, as ventral ones. Frons black to black-brown; weakly subshiny, with dense greyish pollinosity throughout; with dispersed medium long straw-yellow pile. Face black to black-brown; weakly subshiny, with dense greyish pollinosity, facial tubercle weakly shining black, posterolateral margin of mouth shining brownish; in parts with dispersed long pale pile; facial tubercle strongly pronounced. Gena colour and pollinosity as ventral lateral margin of face; with short to long pale pile. Occiput black-brown, covered with dully grey pollinosity; with dispersed pale pile, except dorsally where pile black adjacent to vertex. Antennal segments black-brown, basoflagellomere apically more brownish, arista pale yellow.

***Thorax*** (Fig. 57). Scutum mainly subshiny black; sparse grey pollinose, more densely so along margins and transverse suture; pilosity variable, usually predominantly pale yellow, sometimes more or less interspersed with short black pile. Scutellum apical margin weakly rounded, distinctly marginated, slightly more than twice as wide as long; pale yellow, anterior margin narrowly black-brown; with short pale pile. Pleura ground colour black-brown, sparsely greyish pollinose; covered with dispersed long pale pile except on meron, dorsomedial anepimeron, ventral part of katepimeron, anterior part of katepisternum and anterior anepisternum.

**Figures 68–73.**
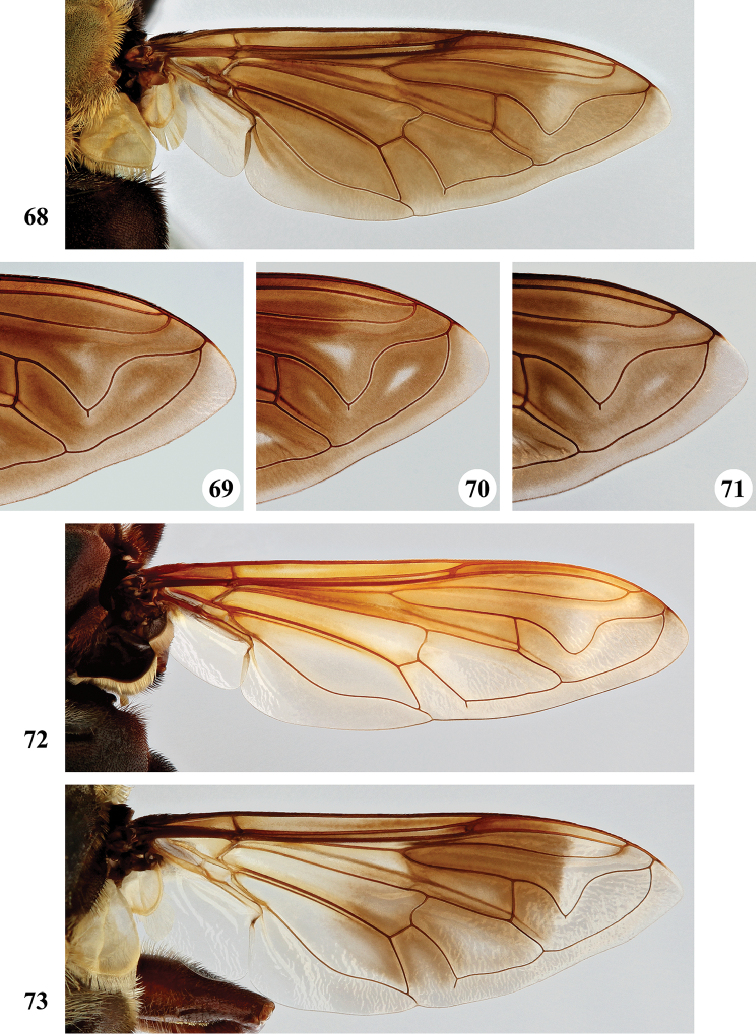
*Senaspis* species, right wing **68***S. elliotii* Austen (♂) **69–71***S. elliotii* Austen close-up apical end **72***S. flaviceps* Macquart (♂) **73***S. haemorrhoa* (Gerstaecker) (♂).

***Legs***. Dark brown to reddish brown; with short dark pile, along posterior margin of pro- and mesofemora with longer pale pile except apically, metafemur predominantly long pale pile in basal two-thirds. Metaleg (Fig. 85), femur moderately thickened, with one distinct ventral swelling in apical fifth, a second less developed thickening proximal of this; tibia thickened and slightly curved, pile along ventral margin more dense.

***Wing*** (Fig. 73). Largely hyaline, only with very faint yellowish brown tinge; with distinct dark brown macula running from anterior margin and covering most of stigma, parts of cell r_1_ and r_2+3_, distal part of cell br and basal part of r_4+5_, decreasing in intensity on anterior part of cell dm, macula well demarcated apically but decreasing gradually in colour basally. Calypters white; with fringe of white pile. Cell r_1_ closed, petiole usually approx. equal to height of base of stigma, rarely shorter. Vein R_4+5_ sinuate and short appendiculate, rarely appendix missing.

**Figures 74–76.**
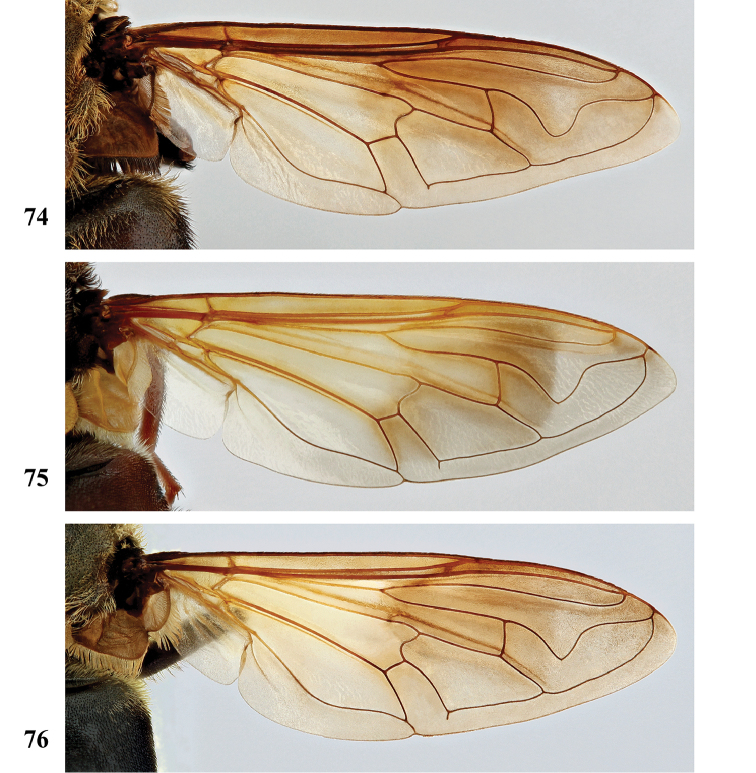
*Senaspis* species, right wing **74***S. melanthysana* (Speiser) (♂) **75***S. nigrita* (Bigot) (♂) **76**S. nr umbrifera (Walker) (♂).

***Abdomen*** (Fig. 95). Uniformly subshiny brown to reddish brown; tergum IV, postabdomen and sometimes posterior margin of tergum III conspicuously orange to orange-red; with short dark pile, except tergum I completely, anterior half of tergum II and anterior fourth of tergum III with longer silvery to pale yellow pile; lateral margins with pale pile; orange areas with pale orange pile. Male genitalia as in Fig. 106.

**Female.** As male except for the following character states: Eye dichoptic (Figs 30, 31), facets equal to subequal in size all over. Frons subshiny black to black-brown, in medial part with greyish pollinosity, pilosity shorter and more silvery pale except in front of ocellar triangle where short and black. Thorax, pilosity usually more extensively black, usually as fasciae. Abdomen pale pilosity along lateral margins missing; pale pilosity in tergum II at most in anterior third, tergum III predominantly with black pile. In general, thoracic and abdominal pale pilosity more silvery than in male.

#### Distribution.

Angola, Benin, Botswana, Burundi, Comoros, Democratic Republic of the Congo, Ethiopia, Guinea Bissau, Kenya, Malawi, Mozambique, Namibia, Nigeria, Republic of the Congo, Rwanda, São Tomé and Príncipe, Somalia, South Africa, Tanzania, Togo, Uganda, Zambia and Zimbabwe.

**Figures 77–79.**
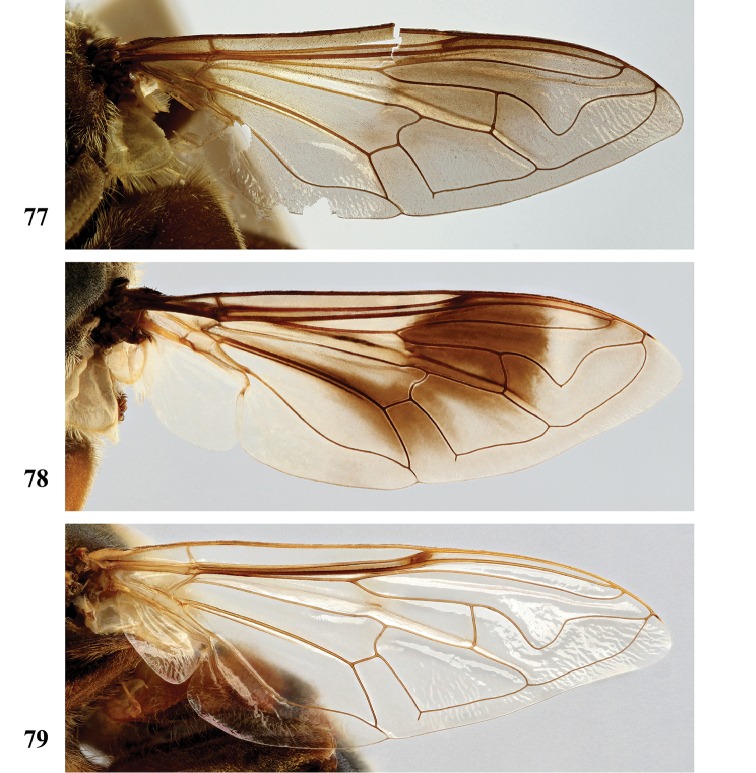
*Senaspis* species, right wing **77***S. umbrifera* (Walker) (♂ holotype) **78***S. xanthorrhoea* (Bezzi) (♂) **79***S. pennata* (Hervé-Bazin) (♀).

#### Comments.

The type material of *S. haemorrhoa* (both male and female specimens according to the original description) could not be traced.

*Senaspis griseifacies* was proposed as a species by Bezzi (1908), who provided a short description. Bezzi (1912) included *griseifacies* and *haemorrhoa* Gerstaecker in his key for the genus *Protylocera* (page 416) and provided a more detailed description of *S. griseifacies*. He differentiated the latter from *S. haemorrhoa* mainly on characters of the wing (anterior margin basally more hyaline in *S. griseifacies*, infuscated in *S. haemorrhoa*) and the abdomen (terga II and III along posterior margin yellow in *S. haemorrhoa*, dark in *S. griseifacies*). Examination of long series of *S. haemorrhoa* and of type material of *S. griseifacies* has shown these characters to be variable. In addition, Bezzi (1912) contradicted the abdominal character partially in his detailed redescription where he indicated that in female specimens of *S. griseifacies* the “è spesso rosso anche l’orlo posterior del terzo segment” (partially red along posterior margin of the third segment). Both species are considered conspecific and thus *S. griseifacies* is considered junior synonym of *S. haemorrhoa*. Hervé-Bazin (1914) already alluded to this when indicating that *S. griseifacies* should be considered perhaps as a mere variety of *S. haemorrhoa*. An identification label ‘*Senaspis haemorrhoa*’ of 1996 by C. Kassebeer on one of the types of *S. griseifacies* at the KBIN collection also alludes to the synonymy but apparently was never formally published.

**Figures 80–85.**
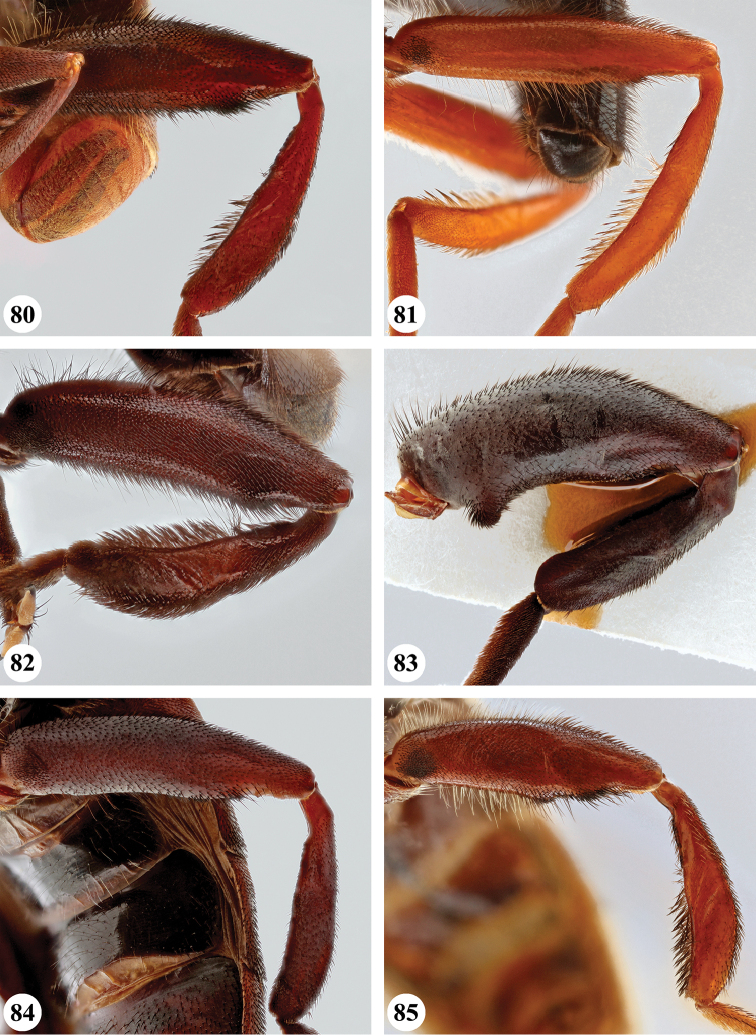
*Senaspis* species, hind leg, anterior view **80***S. dentipes* (Macquart) (♂) **81***S. dibapha* (Walker) (♂) **82***S. elliotii* Austen (♂) **83***S. flaviceps* Macquart (♂) **84***S. flaviceps* Macquart (♀) **85***S. haemorrhoa* (Gerstaecker) (♂).

### Senaspis melanthysana

Taxon classificationAnimaliaDipteraSyrphidae

(Speiser, 1913)

DEF3A16C-793B-534A-9CD9-23287183A425

Figs 6 , 32–35 , 58 , 74 , 86 , 96 , 108 , 109

Protylocera melanthysana Speiser, 1913: 122.

#### Differential diagnosis.

**Figures 86–90.**
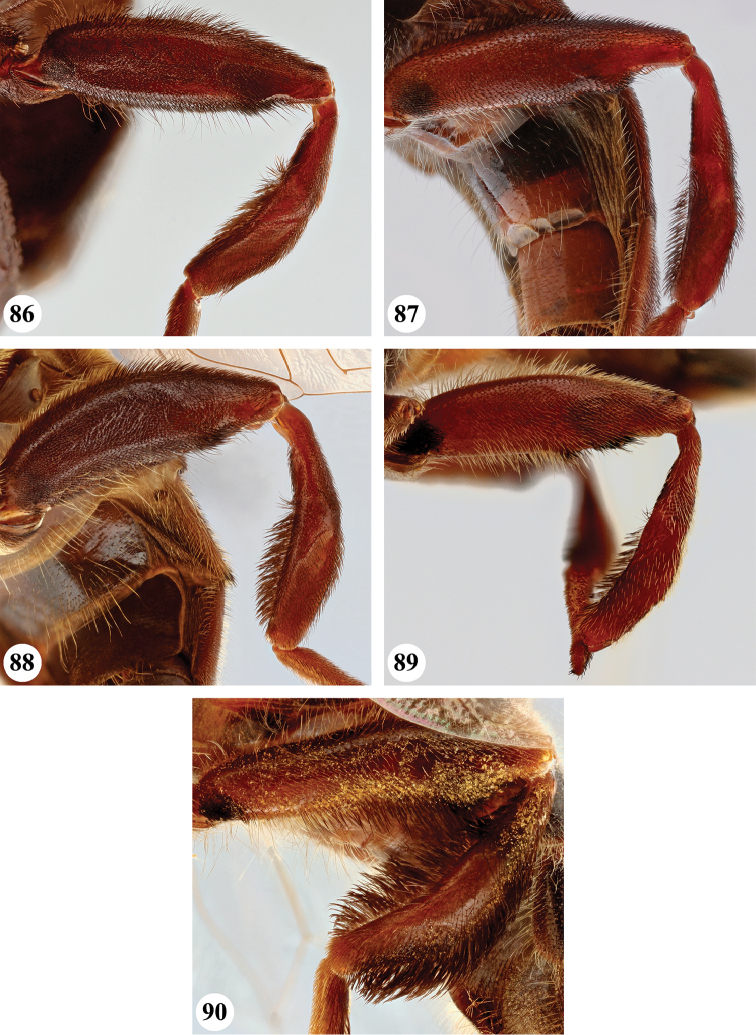
*Senaspis* species, hind leg, anterior view **86***S. melanthysana* (Speiser) (♂) **87***S. nigrita* (Bigot) (♂) **88**S. nr umbrifera (Walker) (♂) **89***S. xanthorrhoea* (Bezzi) (♂) **90***S. pennata* (Hervé-Bazin) (♀).

A dark species (Fig. 6) without distinct macula on wing but with darker medial area (Fig. 74). It can be differentiated from *S. nigrita* by the basal half of the scutellum largely concolourous with the scutum (Fig. 58) (scutellum largely yellow, contrasting with dark scutum in *S. nigrita*; Fig. 59). It resembles strongly *S. umbrifera* but can be differentiated by the following characters: metafemur moderately thickened and straight to slight convex ventrally (Fig. 86) (thicker and concave in *S. umbrifera*; Fig. 88); abdomen with long dark pile along lateral margins (Fig. 96) (mixed short pale and dark pile in *S. umbrifera*; Figs 98, 99); long dark pile on all sterna (Fig. 6) (pale pile on sterna II and III in *S. umbrifera*; Fig. 10).

#### Type.

*Protylocera melanthysana* Speiser: Holotype, female, CAMEROON, Soppo am Kamerunberge, von Rothkirch [institutional depository unknown; not examined].

#### Examined material.

Cameroon • 1♀; Yaoundé; Molez leg.; MNHN. Democratic Republic of the Congo • 1♂; Bamania; 11 May 1924; J. Béquaert leg.; KMMA • 1♀; Bayenge, Wamba; 8 Jul. 1956; R. Castelain leg.; KMMA • Eala; 3♂♂; Eala; 29 Jul. 1935; J. Ghesquière leg.; KBIN • 1♂; same collection data as for preceding; 22 Aug. 1935 • 1♂; same collection data as for preceding; Sep. 1935 • 1♂; same collection data as for preceding; 6 Feb. 1936 • 1♀; same collection data as for preceding; Mar. 1936 • 1♂; same collection data as for preceding; Aug. 1936 • 1♀; same collection data as for preceding; Oct. 1936 • 1♀; same collection data as for preceding; Jan. 1935; KMMA • 1♂; same collection data as for preceding; Aug. 1935 • 1♂; Poko; Aug. 1913; NHMUK • 5♂♂ 1♀; Stanleyville [= Kisangani]; Mar. 1915; Lang and Chapin leg.; AMNH • 1♀; same collection data as for preceding; NHMUK • 1♀; same collection data as for preceding; CNC • 6♂♂ 3♀♀; same collection data as for preceding; KMMA • 1♂; same collection data as for preceding; Apr. 1915; AMNH • 1♂; same collection data as for preceding; CNC • 1♀; same collection data as for preceding; 2 Apr. 1915; AMNH • 1♂; same collection data as for preceding; 5 Apr. 1915 • 1♂ 1♀; same collection data as for preceding; 6 Apr. 1915 • 1♀; same collection data as for preceding; 7 Jul. 1915. Kenya • 1♀; Ngong; Apr. 1944; V.G.L. van Someren leg.; NHMUK. Uganda • 1♀; Entebbe; 1–11 Sep. 1911; S.A. Neave leg.; NHMUK • 1♀; same collection data as for preceding; 12–20 Jan. 1912.

**Figures 91–96.**
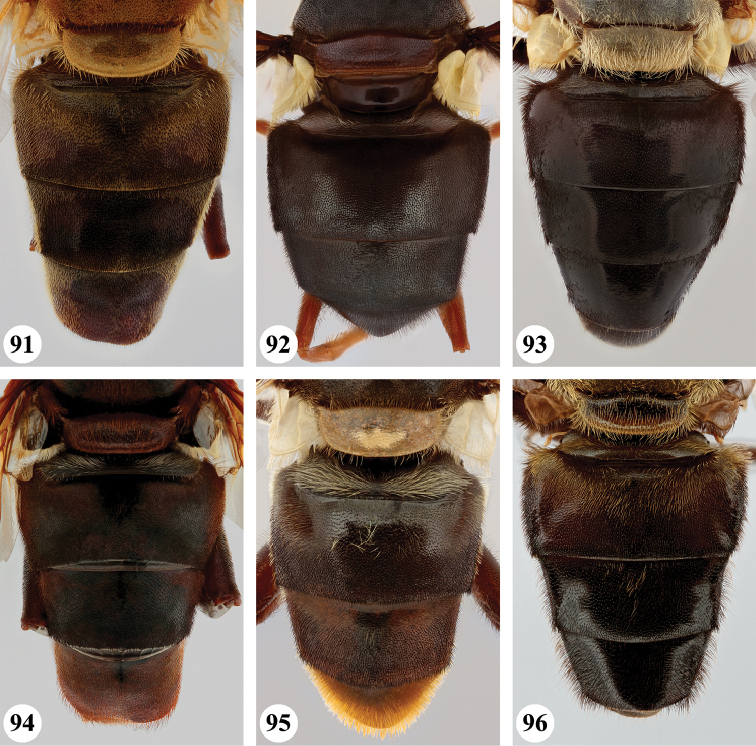
*Senaspis* species, abdomen, dorsal view **91***S. dentipes* (Macquart) (♂) **92***S. dibapha* (Walker) (♂) **93***S. elliotii* Austen (♂) **94***S. flaviceps* Macquart (♂) **95***S. haemorrhoa* (Gerstaecker) (♂) **96***S. melanthysana* (Speiser) (♂).

#### Description.

Body length: 12.6–19.0 mm. Wing length: 9.5–14.3 mm.

**Male** (Fig. 6). Head (Figs 32, 33). Eye bare; holoptic, eye contiguity for distance equal to length or 1.5 times length of ocellar triangle, facets dorsally slightly larger, at most twice as large in diameter as ventral ones. Frons black-brown; largely subshiny with black pollinosity in dorsal fifth only, along eye margins narrowly silver-grey pollinose; dispersed short dark pile, dorsally somewhat longer. Face black-brown; subshiny with pale brownish to greyish pollinosity, in parts more densely so, medial part and ventral lateral parts largely devoid of pollinosity; pollinose parts with dispersed long pale pile; facial tubercle strongly pronounced. Gena as pollinose parts of face; with short to long pale pile. Occiput black-brown, covered with dully grey pollinosity; with dispersed pale pile except dorsally where sometimes darker yellow to black. Antennal segments black-brown, arista orange-brown.

***Thorax*** (Fig. 58). Scutum subshiny black, with brownish to brownish grey pollinosity; with short pale pile. Scutellum apical margin rounded, distinctly marginate, ca. 2.5–3 times as wide as long; pale brownish, anterior margin narrowly darker brown; with short pale pile, longer along margin. Pleura ground colour black-brown, greyish pollinose, anepimeron less so; covered with dispersed long pale pile except on meron, dorsomedial anepimeron, ventral part of katepimeron, anterior part of katepisternum and anterior anepisternum, pilosity on posterior anepisternum more conspicuous.

***Legs***. Mainly black to black-brown; with short black pile, along posterior margin of pro- and mesofemora and along ventral margin of metafemur with longer dark pile, sometimes base of pro- and mesofemora narrowly with more pale brownish pile; along ventral margin of metafemur with longer dark pile, basally with anterodorsal patch of longer dark pile. Metaleg (Fig. 86), femur moderately thickened, with ventral swelling in apical fifth; tibia slightly bent at base, thickened and curved, pile along ventral margin in apical half to two-thirds longer and more dense.

***Wing*** (Fig. 74). Usually distinct fumose yellow-brown tinge, especially in medial part cell r_1_, basal part r_2+3_ and distal part R; towards posterior margin and apex more greyish, sometimes posteriorly more hyaline. Calypters dark brown, concolourous with or darker than medial part wing; with fringe of black pile. Cell r_1_ closed and petiolate, petiole at most as long as half the height of base of stigma. Vein R_4+5_ sinuate; usually not appendiculate, rarely with short appendix.

***Abdomen*** (Fig. 96). Uniformly subshiny brown to black-brown; with short dark pile, except tergum I, and anterolateral parts of tergum II where pale; pilosity along lateral margins of all terga conspicuous and dense, along tergum IV and postabdomen longer. Postabdomen small. All sterna with dispersed long dark pile. Male genitalia as in Figs 108, 109.

**Female.** As male except for the following character states: Eye dichoptic (Figs 34, 35), facets equal to subequal in size. Frons with black pollinose fascia in dorsal part for length at least equal to ocellar triangle, bordered by very narrow and less dense greyish-brown fascia. Abdomen, pilosity along lateral margins of all terga short and more dispersed.

#### Distribution.

Cameroon, Democratic Republic of the Congo, Uganda. Also recorded from Kenya (De Meyer et al. 1995) but this needs to be confirmed as the material was unavailable for this study.

**Figures 97–101.**
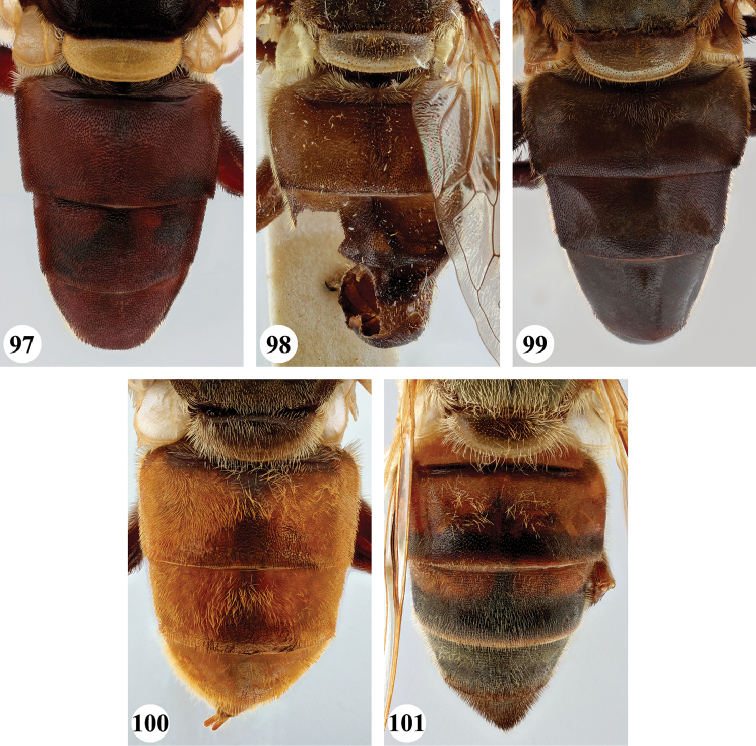
*Senaspis* species, abdomen, dorsal view **97***S. nigrita* (Bigot) (♂) **98***S. umbrifera* (Walker) (♂ holotype) **99**S. nr umbrifera (Walker) (♂) **100***S. xanthorrhoea* (Bezzi) (♀) **101***S. pennata* (Hervé-Bazin) (♀).

#### Comments.

For similarities and differences with *umbrifera*, see comments under the latter. Both species occur sympatrically, like in the Democratic Republic of the Congo (Kisangani) and Uganda (Entebbe) (see material examined), but *S. umbrifera* has a larger distribution throughout. The holotype could not be found but the original description defines the main diagnostic character states and corresponds with material studied.

**Figures 102–113.**
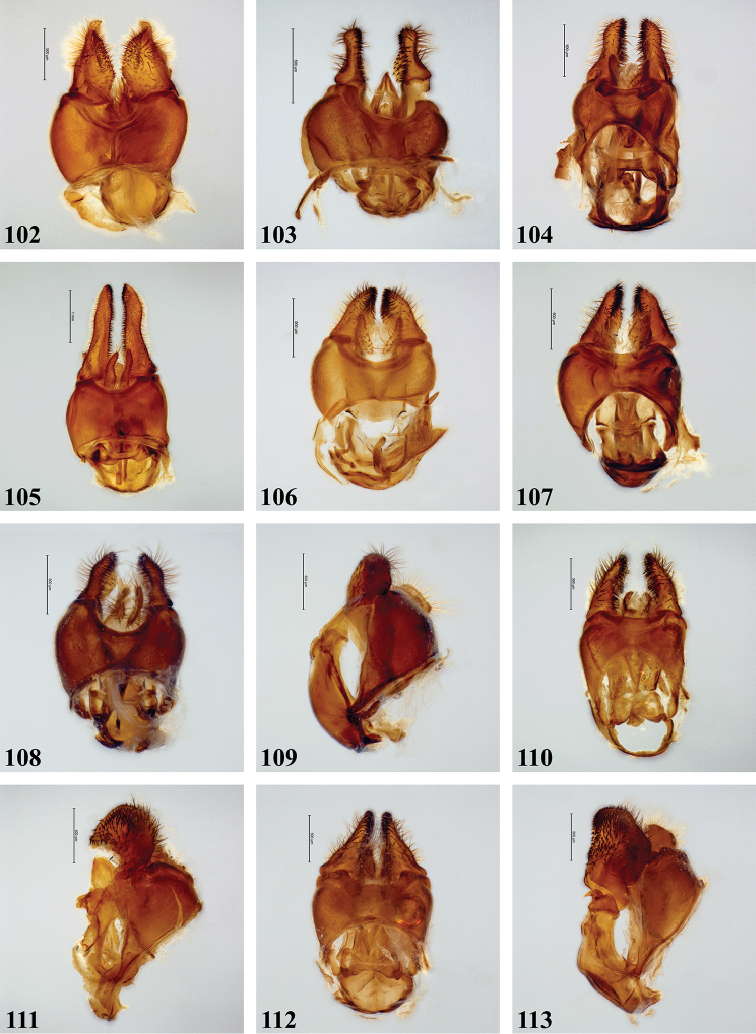
*Senaspis* species, male terminalia **102–108, 110, 112** dorsal view **109, 111, 113** lateral view **102***S. dentipes* (Macquart) **103***S. dibapha* (Walker) **104***S. elliotii* Austen **105***S. flaviceps* Macquart **106***S. haemorrhoa* (Gerstaecker) **107***S. nigrita* (Bigot) **108, 109***S. melanthysana* (Speiser) **110, 111***S. umbrifera* (Walker) (holotype) **112, 113**S. nr umbrifera (Walker).

### Senaspis nigrita

Taxon classificationAnimaliaDipteraSyrphidae

(Bigot, 1859)

66EEEDF5-8397-5B93-929D-33E91AFC394C

Figs 7 , 36–39 , 59 , 75 , 87 , 97 , 107

Dolichomerus nigritus Bigot, 1859: 431.

#### Differential diagnosis.

A brown to black-brown species (Fig. 7) without distinct macula on wing but with darker medial area (Fig. 75). It can be diferentiated from other *Senaspis* species with dark scutum and similar wing marking (*S. melanthysana*, *S. umbrifera*) by the contrasting yellow scutellum (Fig. 59) (scutellum largely concolourous with scutum, at least along basal half in other species (Figs 58, 61).

#### Examined material.

*Dolichomerus nigritus* Bigot: ***Lectotype*** (hereby designated), female, “Syn- // type” “ex. coll. Bigot. // Prs. by // G.H. Verrall. // B.M. 1901–14.” “SYNTYPE ♀ of // Dolichomerus // nigritus Bigot // MADAGASCAR” “BMNH(E) # // 230785” “NHMUK010369878” “LECTOTYPUS” [NHMUK]. ***Paralectotype***, female, “Syn- / /type” “SYNTYPE ♀ of // Dolichomerus // nigritus Bigot // MADAGASCAR” “ex. coll. Bigot. // Pres. by // G.H. Verrall. // B.M.1901–14.” “BMNH(E) # // 230786” “PARA- // LECTOTYPUS” [NHMUK].

#### Other material.

Madagascar • 2♂♂ 1♀; 5 km N Ampotaka, Beloha; 5–15 Mar. 2009; M. Irwin and R. Harin’Hala leg.; CDFA • 1♀; Ambatobevandza; 1919; R. Decary leg.; MNHN • 1♀; Ambatolampy; 1931; Lasère leg.; MNHN • 1♀; Ambatosoratra; Nov. 1960; P. Soga leg.; MNHN • 1♀; Ambodivoangy; Jan. 1967; J. Vadon leg.; KMMA • 1♀; Ambre; Oct. 1902; MNHN • 1♀; Ampassamadinika, Tamatave; 26 Jan. 1992; A. Pauly leg.; KMMA • 1♀; Analmalotra; 1 Nov. 1993; C. Kassebeer leg.; CDFA • 1♀; Andasibe National Park; 17 Dec. 2017; Schmid-Egger leg.; CDFA • 1♀; Andranobe Forest, route d’Andriamena; MNHN • 1♀; Anja Reserve, 10 km SSW Ambalavao; 24 Dec. 2017; Schmid-Egger leg.; CDFA • 1♀; Ankaratra Massif, Manjakatompo; Dec. 1951; Benoist leg.; KMMA • 1♂; Ankaratra Massif, Manjakatompo; Jan. 1958; B. Stuckenberg leg.; NMSA • 1♂; Anosibé; Breuning leg.; KMMA • 1♀; Bekily; Mar. 1930; A. Seyrig leg.; MNHN • 1♂; same collection data as for preceding; May 1933 • 1♂; same collection data as for preceding; Jun. 1933 • 1♂ 5♀♀; Belambo, 20 km NW Boriziny, Mahajanga; 4 Jan. 2007; A.H. Kirk-Spriggs leg.; AMGS • 1♀; Betafo, Ambositra and Miandrivazo regions, Central; 1905; Bouet leg.; MNHN • 1♀; Betsileo; NHMUK • 1♀; Brickaville; Jul. 1959; Sigwalt leg.; MNHN • 2♂♂ 3♀♀; Bevilany, Tulear, 12 Apr. 1968; K.M.G. and P.D. leg.; NHMUK • 1♀; same collection data as for preceding; 14–18 Apr. 1968 • 1♂; Didy; 12 Apr. 1992; A. Pauly leg.; KMMA • 1♂; same collection data as for preceding; 16 Apr. 1992 • 1♀; Fampanambo; 1962; J. Vadon leg.; KMMA • 1♀; Fianarantsoa; A. Bigneux leg.; CNC • 2♀♀; Forest Belt; S. Hutchins leg.; NHMUK • 1♀; Fort Dauphin [= Taolagnaro]; NHMUK • 1♀; Foulpointe [= Mahavelona]; 12 Nov. 1993; A. Pauly leg.; KMMA • 1♂ 2♀♀; same collection data as for preceding; May 1995 • 1♂ 5♀♀; Imerina, Andrangoloaka Forest; A. Grandidier leg.; MNHN • 1♀; Ivoloina; Mar. 1960; Sigwalt leg.; MNHN • 1♂; Lac Froid, Ambatolampy; 11–15 Dec. 1957; B. Stuckenberg leg.; NMSA • 1♀; 7 km N Joffreville; 20 Mar.–7 Apr. 2001; R. Harin’Hala leg.; CDFA • 1♂ 2♀♀; Mananjara Province; 1910; Goissaud leg.; MNHN • 1♀; Maroantsetra; J. Vadon leg.; MNHN • 1♀; same collection data as for preceding; Nov. 1959; KMMA • 1♀; Montagne d’Ambre; L. Chopard leg.; MNHN • 1♀; Ranomafana National Park; 21 Dec. 2017; Schmid-Egger leg.; CDFA • 1♀; Ranomafana National Park, Radio Tower; 16 Nov. 2001; M.E. Irwin; F.D. Parker and R. Harin’hala leg.; CDFA • 3♀♀; Ranomafana National Park, ValBio Research Centre; 15–17 Jan. 2007; A.H. Kirk-Spriggs leg.; AMGS • 4♀♀; same collection data as for preceding; 13 Jan. 2014; M. Hauser leg.; CDFA • 3♀♀; Ranomafana National Park, Talatakely; 11 June 2014; A.H. Kirk-Spriggs and R. Harin’Hala leg.; BMSA • 1♀; Sakalava Beach; 7–25 Jun. 2001; R. Harin’Hala leg.; CDFA • 1♀; Tananarive; 1897; E. Dorr leg.; MNHN • 1♂ 5♀♀; same collection data as for preceding; 1914; G. Waterlot leg.; • 1♂ 1♀; same collection data as for preceding; 1916 • 3♀♀; same collection data as for preceding; 1919 • 1♀; same collection data as for preceding; 22 Dec. 1920; R. Decary leg. • 1♂; same collection data as for preceding; 29 Dec. 1920 • 1♀; same collection data as for preceding; 6 Jan. 1921 • 1♀; same collection data as for preceding; 17 Jan. 1921 • 1♀; same collection data as for preceding; 1921 • 1♀; same collection data as for preceding; Jan. 1922 • 1♀; same collection data as for preceding; 1931; Lasère leg. • 2♂♂; Tananarive; Nov. 1952; E.S. Brown leg.; NHMUK • 1♂ 1♀; Tananarive; Jan. 1958; B. Stuckenberg leg.; NMSA • 1♀; Tananarive; 8 Jan. 1971; H May Daly leg.; NHMUK • 1♀; Tananarive; [no date]; MNHN • 1♀; same collection data as for preceding; KMMA • 1♀; Tananarive; [no date]; Le Moult leg.; MNHN • 1♀; same collection data as for preceding; Leray leg. • 1♀; Tsimbazaza, Antanan. Park; 16–22 Oct. 1993; C. Kassebeer leg.; CNC • 2♂♂; Vitambani Forest, 5 km N Ampotaka, Beloha; 12 Aug.–29 Sep. 2009; M. Irwin and Rin’ha leg.; CDFA • 1♂; [no locality]; 1894; P. Camboué leg.; MNHN• 1♀; 1898; Delcroix leg.; MNHN • 1♀; 1906; MNHN • F. Geay leg.; MNHN • 2♂♂ 13♀♀; P. Camboué leg.

#### Description.

Body length: 14.2–18.2 mm. Wing length: 8.7–13.5 mm.

**Male** (Fig. 7). Head (Figs 36, 37). Eye bare; holoptic, eye contiguity for distance equal to length of ocellar triangle, facets dorsally slightly larger, approx. three times as large in diameter as ventral ones. Frons black-brown; subshiny with brownish pollinosity; long pale yellow pile, especially dorsally and along eye margin. Face black-brown; subshiny with pale brownish to greyish pollinosity, in parts more densely so, ventral lateral margins largely devoid of pollinosity; in parts with dispersed long pale pile; facial tubercle strongly pronounced. Gena colour and pollinosity as ventral lateral margins of face; with short to long pale pile. Occiput black-brown, covered with grey pollinosity; with dispersed pale pile except dorsally where sometimes darker yellow. Antennal segments black-brown, basoflagellomere apically more brown, arista orange-brown.

***Thorax*** (Fig. 59). Scutum subshiny black, weak brownish pollinose; with short pale brown pile in anterior half, posterior part more black pile; postpronotum and notopleuron with longer mixed pale and dark pile. Scutellum apical margin weakly rounded, distinctly marginated, three times as wide as long; pale yellow to straw yellow, anterior margin narrowly black-brown; with short pale pile, along posterior margin somewhat longer. Pleura ground colour black-brown, covered with dispersed long pale or dark pile except on meron, dorsomedial anepimeron, ventral part of katepimeron, anterior part of katepisternum and anterior anepisternum.

***Legs***. Brown to black-brown, sometimes more rufous; with short black pile, along posterior margin of pro- and mesofemora with longer pale pile, along ventral margin of metafemur with longer dark pile. Metaleg (Fig. 87), femur slightly thickened, with two distinct ventral swellings in apical third, swellings with dispersed short thick setae; tibia bent at base, thickened and slightly curved, pile along ventral margin in apical half to two-thirds longer and more dense.

***Wing*** (Fig. 75). Faint yellowish tinge, near cross-veins r-m and bm-cu more yellow-brown but no distinct medial macula; distal part of cells r_2+3_ and r_4+5_ more hyaline, as well as along posterior margin, paler areas not distinctly demarcated; alula largely hyaline. Calypters dull straw yellow with fringe of silvery white pile. Cell r_1_ closed, petiole very short, at most half height of base of stigma, rarely longer. Vein R_4+5_ sinuate, appendix absent or present.

***Abdomen*** (Fig. 97). Uniformly subshiny brown to black-brown, sometimes more rufous; with short dark pile except tergum I where more densely silver-grey pile; lateral margins intermixed with short pale pile, especially near postabdomen. Terga with dispersed long pale pile. Male genitalia as in Fig. 107.

**Female.** As male except for the following character states: Eye dichoptic (Figs 38, 39), facets subequal in size. Frons and face pollinosity predominantly greyish; frons with short dark pile dorsally, greyish pile ventrally; with narrow pale fascia dorsally of antennal implant. Thorax dark pile sometimes more extensively so. Leg pilosity sometimes more brownish.

#### Distribution.

Madagascar. Records from Tanzania (Karsch 1888; Smith and Vockeroth 1980) and Uganda (Smith and Vockeroth 1980) could not be confirmed.

### Senaspis umbrifera

Taxon classificationAnimaliaDipteraSyrphidae

(Walker, 1849)

F7618E05-16F5-509B-A054-731C3FD59D5B

Figs 9 , 10 , 40–43 , 44–45 , 60 , 61 , 76 , 77 , 88 , 98 , 99 , 110–113

Merodon umbrifer Walker, 1849: 601.

#### Differential diagnosis.

A dark species (Figs 9, 10) without distinct macula on wing but with darker medial area (Figs 76, 77). It can be differentiated from *S. nigrita* by the basal half of the scutellum largely concolourous with the scutum (Figs 60, 61) (scutellum largely yellow, contrasting with dark scutum in *S. nigrita*; Fig. 59). It resembles strongly *S. melanthysana* but can be differentiated by the following characters: metafemur (Fig. 88) strongly thickened and distinctly concave ventrally (moderately thickened and straight to slight convex ventrally in *S. melanthysana*; Fig. 86); abdomen with mixed short pale and dark pile long lateral margins (Figs 98, 99) (long dark pile in *S. melanthysana*; Fig. 96); long pale pile on sterna, except on sterna IV and V where dark (Fig. 10) (long dark pile on all sterna in *S. melanthysana*; Fig. 6).

#### Examined material.

*Merodon umbrifer* Walker: ***Holotype***, male, “Type” “Holo- // type” “38 // 11. 8 // 292” “HOLOTYPE ♂ of // Merodon // umbrifer Walker // SIERA LEONE // Pres by the Rev. // D.F. Morgan” “merodon // umbrifer. // Wlk.” “NHMUK010369875” [NHMUK].

#### Other material.

(belonging to near umbrifera; see Comments). Benin • 1♀; Niaouli; 10 Dec. 2013; K. Jordaens and G. Goergen leg.; KMMA. Central African Republic • 1♀; Swane de Bébé; 7 Sep. 1970; L. Matile leg.; MNHN. Democratic Republic of the Congo • 1♀; Bamanya; 1–15 Sep. 1963; P. Hulstaert leg.; KMMA • 1♀; Banningville [= Bandundu]; Aug. 1985; A. Fain leg.; KMMA • 1♀; Basankusu; 1949; O.L.V. ten Bunderen leg.; KMMA • 1♂ 1♀; Bokuma; Jul. 1952; P. Lootens leg.; KMMA • 1♂; Eala; Jan. 1935; J. Ghesquière leg.; CNC • 1♂; same collection data as for preceding; 26 Jul. 1935; KBIN • 1♀; same collection data as for preceding; Oct. 1935 • 1♂; same collection data as for preceding; Aug. 1935; KMMA • 2♂♂; Eala, Sep. 1935; KBIN • 1♂; Eala; KMMA • 1♂; Ituri Forest; Jul. 1976; E.B. Babyetagara leg.; CNC • Kavumu to Kabuga road, Kivu; May 1951; H. Bomans leg.; KMMA • 1♀; Kapanga, Lulua; Nov. 1932; F.G. Overlaet leg.; CNC • 1♂; Leopoldville [= Kinshasa]; 1911; Mouchet leg.; KMMA • 1♂ 1♀; Medje; Ituri; Sep. 1910; Lang and Chapin leg.; AMNH • 1♀; same collection data as for preceding; 1–10 Aug. 1910; KMMA • 1♂; same collection data as for preceding; 11–24 Aug. 1910 • 1♂; same collection data as for preceding; 25–30 Aug. 1910 • 1♂; same collection data as for preceding; 26–30 Sep. 1910 • 1♀; same collection data as for preceding; Sep. 1910 • 1♂; Niangara; Nov. 1910; Lang and Chapin leg.; AMNH • 1♂; Poko; Aug. 1913; C.J. Wainwright leg.; NHMUK • 1♀; Rwankwi, N of Lake Kivu; Apr. 1948; J May Leroy leg.; KMMA • 1♀; Shabunda, Lubongola; 1939; Hautmann leg.; KMMA • 1♀; Stanleyville [= Kisangani]; Mar. 1915; Lang and Chapin leg.; AMNH • 2♀♀; same collection data as for preceding; KMMA. Equatorial Guinea • 1♂; Fernando Po [= Bioko Island], Punta Frailes; Oct.–Nov. 1901; L. Fea leg.; MCSNG. Uganda • 1♂; Budongo forest, nr Lake Albert; 1 Apr. 1972; E.B. Babyetagara leg.; CNC • 1♂; Bwindi Impenetrable Forest National Park, Kigezi; 1–10 Jun. 1972; E.B. Babyetagara leg.; CNC; 1♀; Entebbe; 17 Jun. 1972; H. Falke leg.; CNC • 1♀; Entebbe; 1–11 Sep. 1911; S.A. Neave leg.; NHMUK • 1♀; same collection data as for preceding; 12–20 Jan. 1912. • 1♀; Kayonza Forest, Kigezi; May 1972; E.B. Babyetagara leg.; CNC.

#### Description.

Body length: 12.7–17.5 mm (type specimen: 12.7 mm). Wing length: 9.0–12.8 mm (type specimen 9.0 mm).

**Male** (Figs 9, 10) (based on type of *umbrifera* and non-type material listed above as near umbrifera). Head (Figs 40, 41, 44, 45). Eye bare; holoptic, eye contiguity for distance equal to length or 1.5 times length of ocellar triangle, facets dorsally slightly larger, at most twice as large in diameter as ventral ones. Frons black-brown; largely subshiny with black pollinosity in dorsal fifth only, along eye margins narrowly silver-grey pollinose; dispersed short dark pile, dorsally somewhat longer. Face brown to black-brown; subshiny with pale brownish to greyish pollinosity, in parts more densely so, medial part and especially facial tubercle largely devoid of pollinosity; pollinose parts with dispersed long pale pile; facial tubercle strongly pronounced. Gena colour and pollinosity as ventral lateral margins of face; with short to long pale pile. Occiput black-brown, covered with dull grey pollinosity; with dispersed pale pile except dorsally where sometimes darker yellow to black. Antennal segments black-brown, arista orange-brown to brown.

***Thorax*** (Figs 60, 61). Scutum subshiny black, with brownish to brownish grey pollinosity; with short pale pile, usually intermixed with dispersed black pile. Scutellum apical margin weakly rounded, distinctly marginate, three times as wide as long; pale brownish, anterior margin narrowly darker brown; with short pale pile, usually with dark pile on disc (sometimes entirely pale pilose), pilosity along apical margin somewhat longer. Pleura ground colour black-brown, greyish pollinose, anepimeron less so; covered with dispersed long pale pile except on meron, dorsomedial anepimeron, ventral part of katepimeron, anterior part of katepisternum and anterior anepisternum; pilosity on posterior anepisternum more conspicuous.

***Legs***. Brown to black-brown, sometimes more reddish brown; with short black pile, along posterior margin of pro- and mesofemora with longer pile, sometimes more pale in basal part; along ventral margin of metafemur with longer dark pile, basally with anterodorsal patch of longer pale pile. Metaleg (Fig. 88), femur distinctly thickened and moderately curved, with one distinct ventral swelling in apical fifth, short black pile more dense where swollen; tibia slightly bent at base, thickened and curved, pile along ventral margin in apical half to two-thirds longer and more dense.

***Wing*** (Figs 76, 77). Faint yellowish brown tinge, more pronounced in medial part cell r_1_, basal part r_2+3_ and distal part br; towards posterior margin and apex more greyish. Calypters yellow-white to pale brown, with fringe of yellow-white pile. Cell r_1_ closed; if petiolate than petiole at most approx. as long as half the height of base of stigma. Vein R_4+5_ sinuate but not appendiculate.

***Abdomen*** (Figs 98, 99). Uniformly subshiny red-brown to black-brown, anterolateral parts of tergum II sometimes less dark; sometimes indistinct black-brown pollinose medial macula along anterior margin of terga II and III; with short dark pile, except tergum 1, and anterolateral parts of tergum II where pale pile; lateral margins usually dark pilose, at most tergum IV partly pale pilose and terga II and III mixed pale and dark pile. Type of *umbrifera* lateral margins completely pale pilose (Fig. 98). Postabdomen conspicuously swollen. Sterna with dispersed long pale pile, on sterna IV and V darker. Male genitalia as in Figs 110–113 (see Comments).

**Female** (based on material identified as near umbrifera). As male except for the following character states. Head (Figs 42, 43), eye dichoptic, facets equal to subequal in size. Frons with black pollinose fascia in dorsal part for length at least equal to ocellar triangle, bordered by very narrow and less dense greyish-brown fascia. Scutellum, dark pile on disc less outspoken, more predominantly paly pilose. Wing, vein R_4+5_ sometimes very short appendiculate. Abdomen with dark pile along lateral margins of all terga.

#### Distribution.

Benin, Central African Republic, Democratic Republic of the Congo, Equatorial Guinea, Sierra Leone, Uganda (but see Comments).

#### Comments.

The type specimen of *S. umbrifera* originates from Sierra Leone. It is in poor condition and the male genitalia were dissected prior to our study; thus, we cannot exclude a mixing of genitalia with another specimen. When comparing the genitalia with some non-type material, some slight differences were observed regarding the shape of the surstyli, the most explicit difference being the shape of the surstyli in lateral view (curved and pointed in the type species (Fig. 111); straight and not pointed in the other material (Fig. 113) and similar to the shape observed in *S. melanthysana* (Fig. 109). However, no major morphological differences could be observed and except for the differently shaped surstylus the type falls within the observed variability for the characters studied. The only other minor morphological difference observed is that the lateral margin of the abdominal terga of the type is covered with short pale pile while in all other specimens this is black or intermixed black and pale pile. Furthermore, the type is recognized by the entirely pale pile on the scutellum (variable in other specimens); the legs being more red-brown (brown to black-brown in others) and the calypters being yellow-white (yellow-white to pale brown in others). No additional specimens could be obtained from the same region while all non-type male specimens originate from Central Africa (Equatorial Guinea eastwards to Uganda). As the observed differences are very minor, we prefer to take a conservative position by provisionally listing all non-type material under *S. umbrifera* (listed as “Senaspis near umbrifera”). We consider the morphological differences too minor to warrant describing it as a separate species and propose to await additional material, especially from western Africa before making a formal decision. The only differentiating character (pale pile along lateral margins of abdomen) is, however, incorporated in the identification key. All major structures are also illustrated both for the type specimen and representative non-type specimens.

*Senaspis umbrifera* resembles *S. melanthysana* in most respect regarding body coloration and pilosity, and wing markings. However, a number of distinct differences could be discerned as pointed out in the key above: the lower calypter has a fringe of pale hairs (dark in *melanthysana*); the abdominal sterna I–III have long pale pile (dark in *melanthysana*), the lateral margins of the abdominal terga have short pale pile along most of their length (long dark pile in *melanthysana*), and the metafemur is strongly thickened and distinctly curved (moderately thickened and straight in *melanthysana*).

### Senaspis xanthorrhoea

Taxon classificationAnimaliaDipteraSyrphidae

(Bezzi, 1912)

D4837CDC-71AD-569C-9F1E-15E316619684

Figs 8 , 48–51 , 62 , 78 , 89 , 100

Protylocera xanthorrhoea Bezzi, 1912: 416.

#### Differential diagnosis.

A species with a distinct medial dark brown macula on the wing (Fig. 78). It can be differentiated from other *Senaspis* species with a distinct wing macula by the coloration of the abdominal terga which is uniformly dark yellow to yellow-orange (Fig. 100) (at least terga I and II completely dark brown in *S. dentipes* (Fig. 91) and *S. haemorrhoa* (Fig. 95)).

#### Examined material.

*Protylocera xanthorrhoea* Bezzi: ***Holotype***, female, “Protylocera // Type // xanthorrhoea // Bezzi.” “Holo- // type” “Voi. // 20. VI. to // 21. VII.” “B.E. Africa. // C.S. Betton. // 98–12” “Protylocera // xanthorrhoea // n. sp.” “NHMUK010369876” [NHMUK].

#### Other material.

Kenya• 1♂ 1♀; Kasigau; Nov. 1938; V.G.L. van Someren leg.; CNC • 1♂ 1♀; Nairobi; 25 Aug. 1951; L.C. Edwards leg.; NHMUK • 2♀♀; same collection data as for preceding; 14. Dec. 1951 • 1♀; same collection data as for preceding; 22 Dec. 1951.

#### Description.

Body length: 12.6–14.3 mm. Wing length: 9.5–10.4 mm. *Male* (Fig. 8). Head (Figs 48, 49). Eye bare; narrowly dichoptic, eyes separated for width equal to 1–2 facets; narrow separation for distance at most equal to length of ocellar triangle; facets dorsally slightly larger, at most twice as large in diameter as ventral ones. Frons black-brown; subshiny, with sparse greyish to greyish brown pollinosity throughout except dorsally of antennal implant; with medium long pale pile. Face black-brown, more reddish brown along buccal cavity; weakly subshiny, with dense greyish to greyish brown pollinosity, only facial tubercle weakly shining black; in parts with dispersed pilosity of long pale pile; facial tubercle strongly pronounced. Gena colour and pollinosity as ventral lateral margins of face; with short to long pale pilosity. Occiput red-brown, covered with dully grey pollinosity; with dispersed pale pile. Antennal segments black-brown, arista pale yellow.

***Thorax*** (Fig. 62). Scutum weakly subshiny black; with dark brown to black pollinosity; with short pale pile; in medial part intermixed with patches of black pile. Scutellum rounded, clearly marginated, slightly more than twice as wide as long; brown, towards posterior margin gradually more yellowish; with short pale pilosity except on disc where black pile, along apical margin longer pile. Pleura ground colour black-brown, sparsely greyish pollinose; covered with dispersed long pale pile except on meron, ventral part of katepimeron, dorsomedial anepimeron, anterior part of katepisternum and anterior anepisternum.

***Legs***. Reddish brown to orange-brown; with short pale pilosity, along posterior margin of pro- and mesofemora, and dorsal and ventral margin of metafemur with longer pale pile. Metaleg (Fig. 89), femur moderately thickened, with distinct ventral swelling in apical fifth, a second less developed thickening proximal of this, with strong dark setae where swollen; tibia thickened and slightly curved, pile along ventral margin more dense and dark.

***Wing*** (Fig. 78). Largely hyaline, with very faint yellowish brown tinge especially along veins; with distinct dark brown macula running from anterior margin and covering most of stigma, parts of cell r_1_ and r_2+3_, distal part of cell br and basal part of r_4+5_, decreasing in colour on anterior part of cell dm. Calypters yellow-white; with fringe of yellow-white pile. Cell r_1_ closed, petiole variable in length, usually shorter than height of base of stigma. Vein R_4+5_ sinuate, not appendiculate or, at most, with trace of appendix.

***Abdomen*** (as in Fig. 100). Uniformly subshiny dark yellow to yellow-orange, except tergum I and anterior margin of tergum II where darker, in medial part of tergum II dark area extending to halfway tergum; with short pale pilosity, except medially in posterior half of terga II and III where dark pile. Sterna yellow-orange; with long pale pilosity. Male genitalia not dissected.

**Female.** As male except for the following character states: Eye distinctly dichoptic (Figs 50, 51), facets equal to subequal in size. Frons subshiny black to black-brown, dorsally more red-brown; with greyish brown pollinosity dorsally, more greyish pollinose ventrally. Wing medial macula more extensive with dark streak along posterior half of cell bm and basal part of cell cua_1_. Abdomen (Fig. 100), dark area in medial part of tergum II sometimes less extensive.

#### Distribution.

Kenya.

##### Incertae sedis

### Senaspis pennata

Taxon classificationAnimaliaDipteraSyrphidae

(Hervé-Bazin, 1914)

8BF7969B-11A8-559D-9F23-0A220BDD3C7F

Figs 11 , 46 , 47 , 63 , 79 , 90 , 101

Protylocera pennata Hervé-Bazin, 1914: 288.

#### Differential diagnosis.

Different from all other *Senaspis* species by the head in lateral view without distinct protuding frons (Fig. 47); the unmarginated scutellum (Fig. 63), and the completely hyaline wing, with a distinctly open cell r_1_ (Fig. 79).

#### Examined material.

*Protylocera pennata* Hervé-Bazin: ***Holotype***, female, “HOLOTYPUS” “MUSÉE DU CONGO // Kalengwe 16.X.1911 // Dr. Bequaert leg.;” “R. DÉT. // M // 69” “Protylocera // pennata // Hervé-B. ♀ // Type” “RMCA ENT // 000016792” [KMMA].

#### Description.

Body length: 15.0 mm. Wing length: 12.2 mm.

**Female** (Fig. 11). Head (Figs 46, 47). Eye bare; dichoptic, facets equal in size. Frons largely subshiny black-brown in ventral protruding part; dorsally with black pollinosity in front of ocellar triangle for length equal to ocellar triangle, between subshiny and black pollinose part with more dispersed greyish pollinosity continued ventrally narrowly along eye margin; dispersed short dark pile, except for area with greyish pollinosity where pile is pale. Face subshiny brown; with greyish pollinosity, medial part and ventral lateral margins largely devoid of pollinosity; in parts with dispersed long pale pile; facial tubercle weakly pronounced. Gena as pollinose part of face; with short to long pale pilosity. Occiput black-brown, covered with dully grey pollinosity; with dispersed pale pile except dorsally where black. Antennal segments black-brown, arista pale yellow.

***Thorax*** (Fig. 63). Scutum subshiny black, with brownish to brownish grey pollinosity; with short pale brown pile, along lateral margins intermixed with dispersed black pile, pilosity paler posteriorly, pilosity modereately long especially along margins. Scutellum rounded and not marginated, slightly more than twice as wide as long; pale brownish; with pale pilosity except anteriorly on disc where largely orange-brown pile (pile rubbed off in medial part); pilosity moderately long especially along apical margin; Pleura ground colour black-brown, covered with dispersed long pale pile except on meron, dorsomedial anepimeron, anterior part of katepisternum and anterior anepisternum (katepimeron obscured and absence or presence of pilosity not visible).

***Legs***. Brown to black-brown, pro- and mesofemora more orange-brown posteriorly, tarsal segments yellowish except major part of metabasotarsomere; with short black pilosity, along posterior margin of pro- and mesofemora with longer pale pile, tarsal segments, except major part of metabasotarsomere, with short pale pile; pro- and mesotibiae very dense. Metaleg (Fig. 90), femur moderately thickened, with ventral swelling in apical fifth; tibia thickened and curved, pile on ventral and dorsal margins along entire length, at least as long as width of tibia, and very dense.

***Wing*** (Fig. 79). Completely hyaline. Calypters yellow-white with fringe of yellow-white pile. Cell r_1_ distinctly open; vein R_4+5_ sinuate but not appendiculate.

***Abdomen*** (Fig. 101). Subshiny brown to black-brown, weak brownish pollinose; tergum I more yellowish brown; tergum II anterior margin narrowly black, medial anterior half yellowish brown, laterally more extensively so, gradually darkening posteriorly; tergum III with pair of orange-brown fasciae in anterior two-fifths; with short pale pilosity, except tergum V and posterior margin of tergum IV where black pile.

**Male.** Unknown.

#### Distribution.

Democratic Republic of the Congo.

#### Comments.

The position of this species within the genus *Senaspis* is uncertain. While there are some similarities with other *Senaspis* species (maculate eyes, wing venation except cell r_1_ distinctly open, pilosity on metatibia), there are also some distinct differences. For instance, the shape of the head in lateral view (no strongly protruding frons, facial tubercle poorly developed, face extending more ventrally), the scutellum is unmargined, the completely hyaline wing, and the distinctly open cell r_1_. As the available material is limited to a single female specimen, we await additional material and/or further revision of other eristaline representatives from the Afrotropical region before proposing any generic assignment.

## Discussion

The revised genus *Senaspis* now contains ten valid species, i.e., *S. dentipes* (Macquart), *S. dibapha* (Walker), *S. elliotii* Austen, *S. flaviceps* Macquart, *S. haemorrhoa* (Gerstaecker), *S. nigrita* (Bigot), *S. pennata* (Hervé-Bazin), *S. melanthysana* (Speiser), *S. umbrifera* (Walker), and *S. xanthorrhoea* (Bezzi). We herewith place the following species in synonymy: *Senaspis apophysata* (Bezzi) as junior synonym of *S. flaviceps* Macquart, *S. livida* (Bezzi) as junior synonym of *S. dentipes* (Macquart), *S. nigripennis* (Macquart) as junior synonym of *S. dibapha* (Walker), and *S. griseifacies* (Bezzi) as junior synonym of *S. haemorrhoa* (Gerstaecker). The generic characters, as given above in the generic diagnosis, are representative for all species. However, the closed and petiolate cell r_1_ is a variable character with sometimes the petiole missing (see for example *S. dentipes*) or even slightly open (*S. elliotii*). The latter is also the case for *S. pennata*. This species shows a number of morphological differences (see comments listed under the species treatment) with the other species within the genus that makes its placement within this genus uncertain. *Senaspis flaviceps* also is slightly aberrant compared to the other *Senaspis* species: bare katepimeron (pilose in others), presence of a basoventral tubercle in the male metafemur (absent in all others), longer petiole (at least as long as height of base of stigma; shorter in all others) and the larger body size (more than 17 mm; shorter in all others). As *S. flaviceps* is the type species for the genus *Senaspis*, Thompson (pers. comm.) suggested that the generic concept of *Senaspis* should be restricted to *S. flaviceps* and all other taxa placed in a separate genus distinct from *Senaspis*. The available name *Triatylosus* (established as subgenus of *Senaspis* by Hull (1949) with *Xylota dibaphus* Walker as type species) has been proposed to be raised to genus rank and to include all *Senaspis* species except *S. flaviceps*. We, however, propose to wait for more taxonomic revisions for other Afrotropical eristaline groups to have a better understanding of their morphology and their phylogenetic relationships can be ascertained before making changes at generic level.

**Figure 114.**
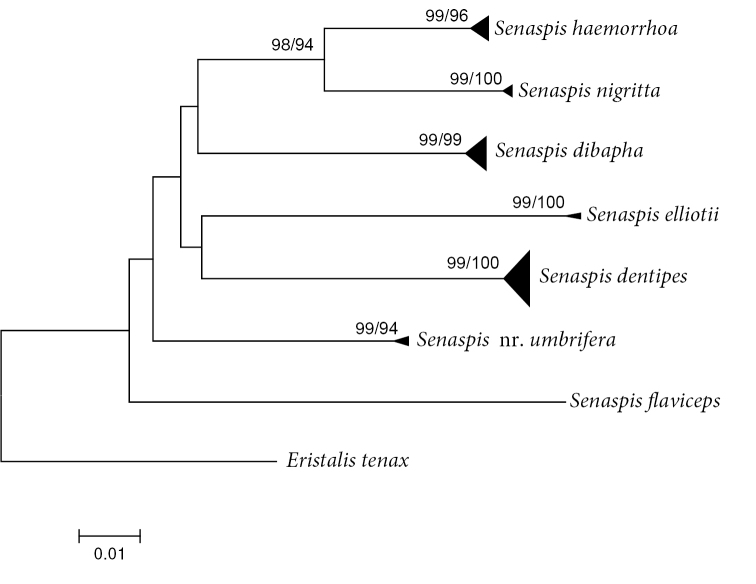
Neighbor-Joining (NJ) and Maximum Likelihood (ML) topology of seven *Senaspis* species and with *Eristalis tenax* as outgroup. Bootstrap values ≥ 70% are presented at the nodes as (NJ/ML).

Besides the position of *S. flaviceps*, no distinct morphological clusters can be recognized among the remaining species although some taxa show morphological similarities. Some *Senaspis* species have a distinct wing marking with a well demarcated dark macula in the medial part (Figs 64, 73, 78). Within this group *S. haemorrhoa* and *S. xanthorrhoea* are similar in appearance with pale pilosity on face and frons (Figs 28–31, 48–51), partial to completely reddish pilosity and coloration of the abdomen (Figs 95, 100), and relatively broadened metafemur with ventrally two subapical swellings (Figs 85, 89). *Senaspis nigrita* shares the latter characteristic with these two taxa (Fig. 87) while the wing marking is similar but with the medial wing marking less pronounced and demarcated (Fig. 75). Also, the male genitalia are similar with the surstyli, in dorsal view, bearing an inner row of stronger setae in the dorsal third (Figs 106, 107). The NJ and ML analysis (Fig. 114) tentatively suggest that *S. haemorrhoa* and *S. nigrita* are sister species (no genetic markers could be obtained for *S. xanthorrhoea*). *Senaspis dentipes* has identical wing markings to *S. haemorrhoa* and *S. xanthorrhoea* but is otherwise morphologically very distinct from both, including differences in male genital structures.

*Senaspis melanthysana* and *S. umbrifera* are also morphologically very similar to each other. Both species have a very similar appearance and a considerable number of specimens were misidentified in either way. Both have the wing markings more diffused with a general darkish tinge slightly more pronounced in the medial part (Figs 74, 76, 77). The general habitus is very dark, with the dorsal part of the thorax covered by a dense short and predominantly pale pile (Figs 6, 9, 10) continuing on the pleura. Morphological differences between both species are listed above in the taxonomic treatment of the taxa. *Senaspis elliotii* shows some similarities in general appearance (Fig. 3) but is clearly distinct by the dense white to yellow pile on the dorsal part of the thorax, contrasting with the black pile on the pleura, and by the wing coloration which is predominantly dark, except along the posterior margin and at the apex (Fig. 68). Its relationship with the other taxa is unclear.

*Senaspis dibapha* does not show any morphological similarities with any of the other taxa within *Senaspis*. The general reddish appearance, morphological character states of additional facial tubercles laterally from the medial one (Figs 16, 18), and the slender metafemur (Fig. 81) are unique. The wing (Fig. 65) is largely darkened, except for the posterior margin, and somewhat similar to *S. elliotii* (Fig. 68).

All seven *Senaspis* species for which we obtained DNA barcodes are grouped as such in the NJ topology (Suppl. material 1: Fig. S1; Fig. 114) with a mean interspecific p-distance of 0.075 (range: 0.052–0.130), largely exceeding the mean intraspecific p-distance of 0.0037 (range: 0.002–0.005). The phylogenetic affinities among the species of this genus will be treated elsewhere (Mullens et al. unpublished).

## Supplementary Material

XML Treatment for Senaspis

XML Treatment for Senaspis dentipes

XML Treatment for Senaspis dibapha

XML Treatment for Senaspis elliotii

XML Treatment for Senaspis flaviceps

XML Treatment for Senaspis haemorrhoa

XML Treatment for Senaspis melanthysana

XML Treatment for Senaspis nigrita

XML Treatment for Senaspis umbrifera

XML Treatment for Senaspis xanthorrhoea

XML Treatment for Senaspis pennata

## Supplementary materials

Supplementary material 1 Figure S1 Data type Neighbor-Joining tree Explanation note Neighbor-Joining tree (K2P) of 64 DNA barcodes of seven Afrotropical *Senaspis* species. *Eristalis tenax* was used as outgroup. https://binary.pensoft.net/file/486726This dataset is made available under the Open Database License (http://opendatacommons.org/licenses/odbl/1.0/). The Open Database License (ODbL) is a license agreement intended to allow users to freely share, modify, and use this Dataset while maintaining this same freedom for others, provided that the original source and author(s) are credited.Marc De Meyer, Georg Goergen, Kurt Jordaens

Supplementary material 2 Table S1. Syrphidae collection Data type list of specimens Explanation note List of specimens used for Neighbor-Joining tree (K2P) of Figure S1. https://binary.pensoft.net/file/486727This dataset is made available under the Open Database License (http://opendatacommons.org/licenses/odbl/1.0/). The Open Database License (ODbL) is a license agreement intended to allow users to freely share, modify, and use this Dataset while maintaining this same freedom for others, provided that the original source and author(s) are credited.Marc De Meyer, Georg Goergen, Kurt Jordaens
