# Supplementary figures and images for: Taxonomic revision of the Afrotropical hover fly genus Senaspis Macquart (Diptera, Syrphidae)
Source: Zookeys. 2020 Dec 14;1003:83–160. doi: 10.3897/zookeys.1003.56557 (PMC7752890; doi:10.3897/zookeys.1003.56557)

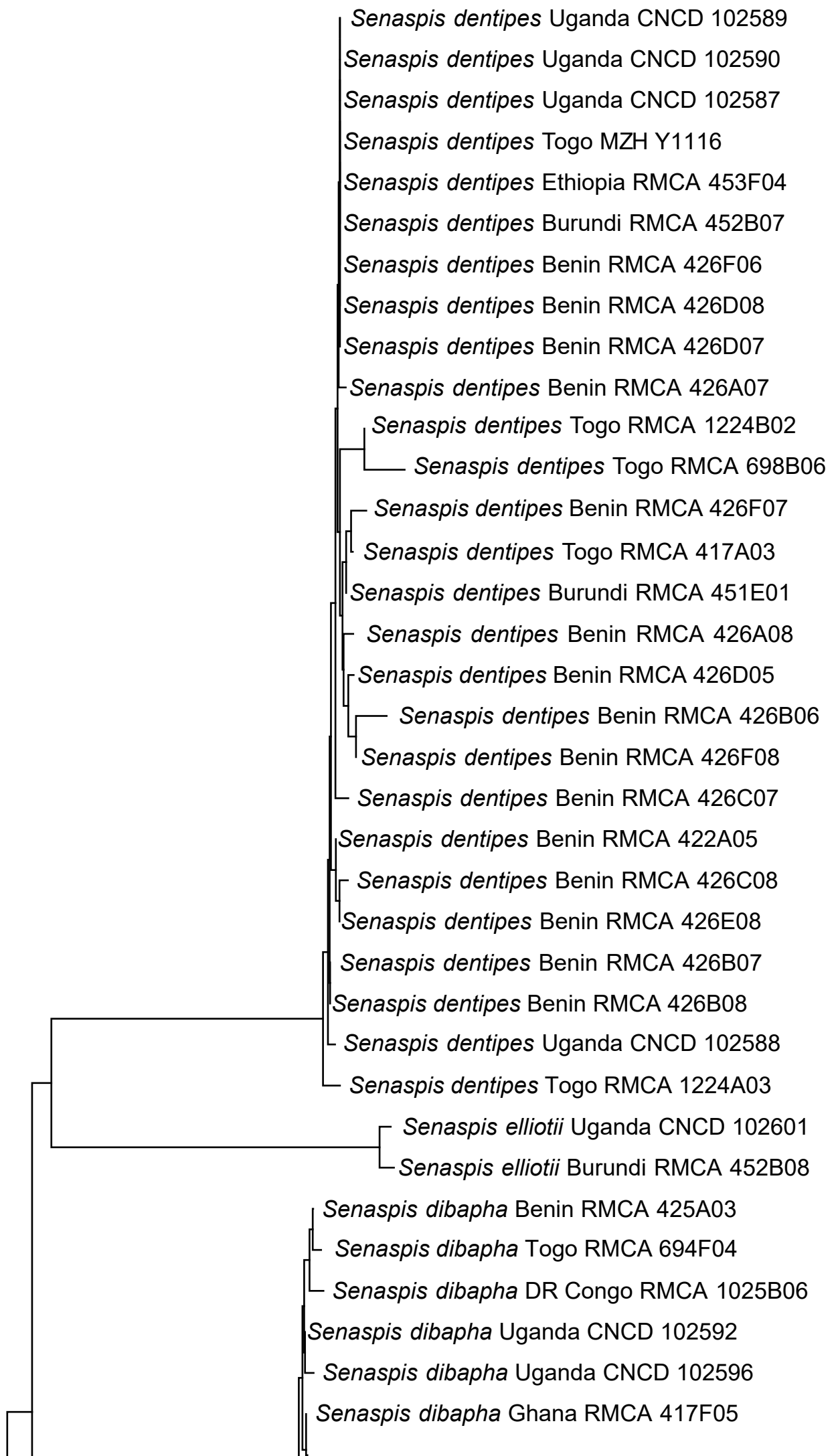

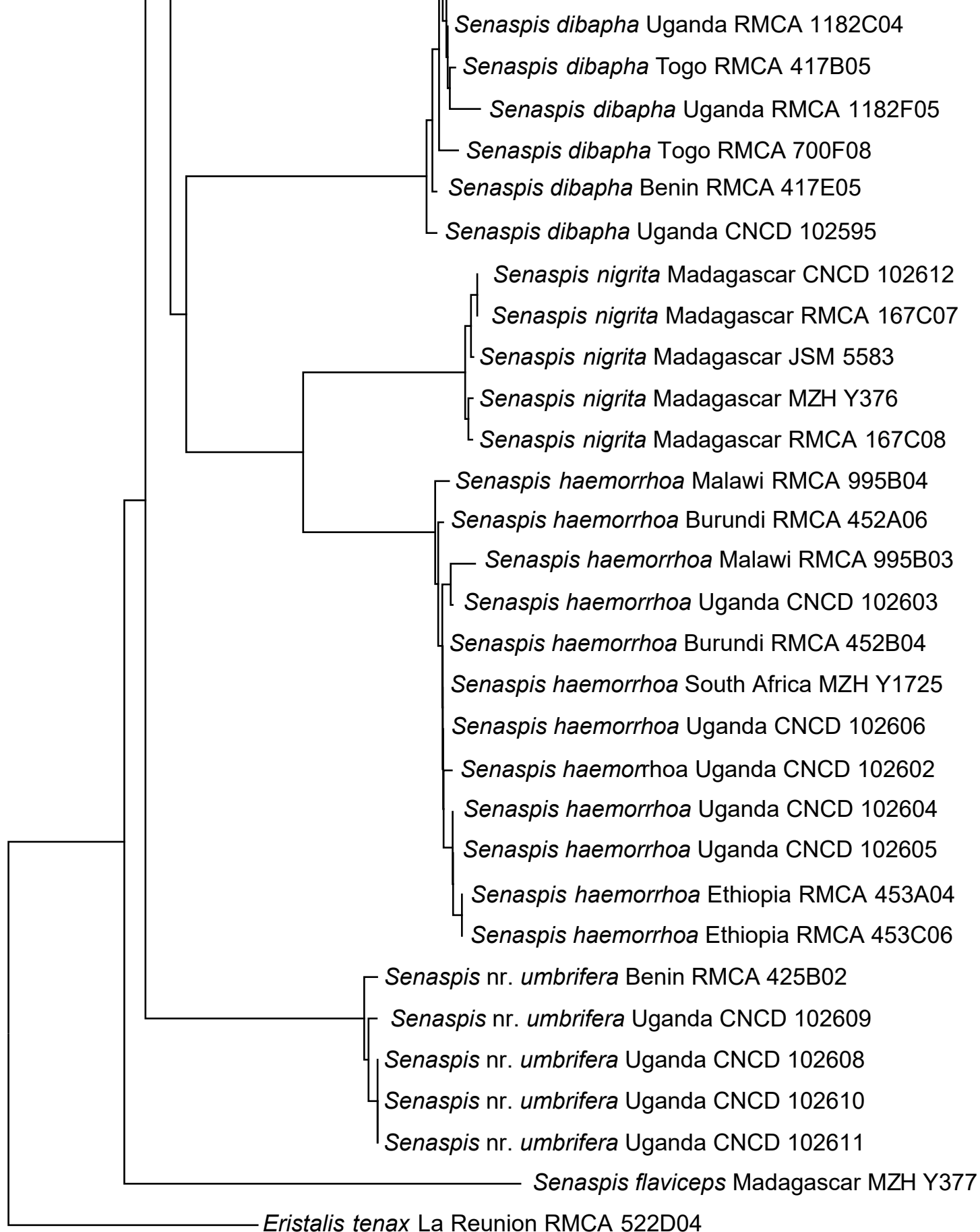

0.0100

Supplement: Supplementary material 1 — Figure S1 [file zookeys-1003-083-s001.pdf]
